# Proximity-Induced Ligation and One-Pot Macrocyclization of 1,4-Diketone-Tagged Peptides Derived from 2,5-Disubstituted Furans upon Release from the Solid Support

**DOI:** 10.1021/acs.orglett.3c02289

**Published:** 2023-09-01

**Authors:** Alex Manicardi, Atiruj Theppawong, Marleen Van Troys, Annemieke Madder

**Affiliations:** † Department of Chemistry, Life Sciences and Environmental Sustainability, University of Parma, Parco Area delle Scienze 17/A, 43124 Parma, Italy; ‡ Organic and Biomimetic Chemistry Research Group, Department of Organic and Macromolecular Chemistry, Ghent University, Krijgslaan 281-S4, 9000 Ghent, Belgium; § Department of Biomolecular Medicine, Ghent University, Technologiepark-Zwijnaarde 75, 9052 Ghent, Belgium

## Abstract

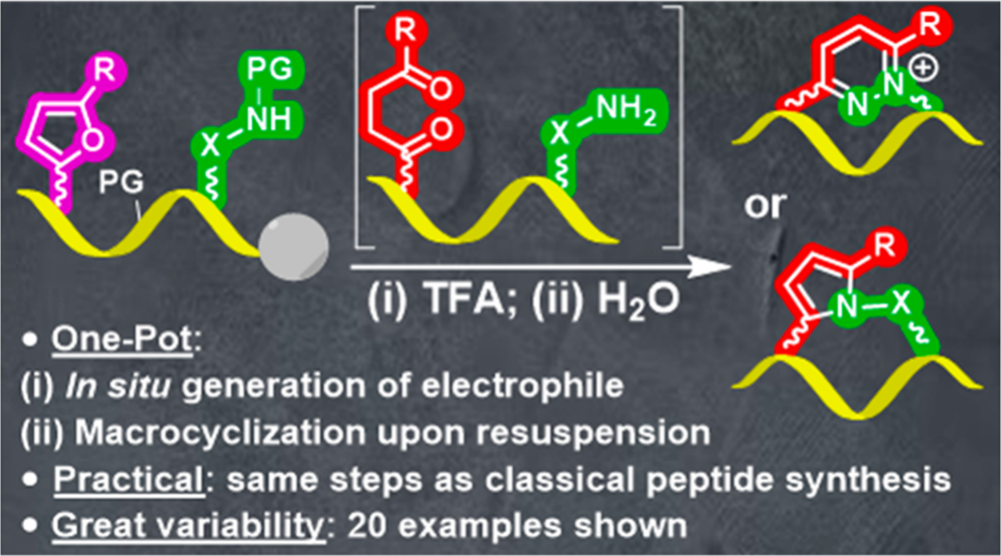

1,4-Dione-containing peptides are generated during the cleavage of 2,5-disubstituted furan-containing systems. The generated electrophilic systems then react with α-effect nucleophiles, following a Paal-Knorr-like mechanism, for the generation of macrocyclic peptides, occurring after simple resuspension of the crude peptide in water. Conveniently, the in situ generation of the electrophile from a stable furan ring avoids the complications associated with the synthesis of carbonyl-containing peptides. Detailed investigation of the reaction characteristics was first performed on supramolecular coiled-coil systems.

Protein–protein interactions (PPIs) are fundamental in various biological processes. PPIs involve short peptide fragments on both partners, and therefore, peptides are often used to interfere with these interactions. Peptide-based drugs exhibit increased efficacy as compared with small molecule inhibitors, making them attractive candidates for therapeutic applications. In this context, the interest in macrocyclic peptides has grown over the years in view of their enhanced target affinity, increased cell membrane penetration, and resistance to proteolytic degradation as compared to linear nonstabilized peptide motifs.^1−7^

Notably, over the past two decades, cutting-edge organic synthetic techniques have revolutionized peptide macrocyclization, and highly effective strategies were developed for modulating the structure and properties of peptides.^8−15^ These approaches can rely on the use of both natural and unnatural amino acids, or a combination thereof, to achieve chemoselective ligations on protected or unprotected peptides.^16^

Despite the notable progress in peptide stapling techniques, challenges persist in existing methodologies, particularly in controlling the reaction order at multiple sites and achieving precise positional selectivity.

In recent years, efforts were increasingly devoted to the realization of one-pot macrocyclization chemistries that rely on simple reaction conditions and the realization of reagent-less approaches. Among these cyclizations, we can find α-ketoacid-hydroxylamine (KAHA) ligations,^17^ acid-catalyzed transamidation of N-terminal maleimides,^18^ Diels–Alder reaction of maleimides on dienes and furans,^19^ cysteine SNAr reaction on thioethers,^20^ CyClick reactions exploiting the intramolecular quenching of an imine intermediate,^21^ or the reversible imminoborane^22^ and oxime^23^ reactions.

Recently, we reported on the possibility of exploiting a 5-methylfuran-2-yl building block as a stable handle to introduce a 2,5-dioxopentanyl (DOP) moiety in peptide nucleic acids and peptides. The DOP reactivity toward α-effect nucleophiles can be exploited in a proximity-induced bio-orthogonal ligation under biologically relevant conditions.^24^ Here, we report on the use of masked 1,4-diones and α-effect nucleophiles for a peptide macrocyclization reaction that works by just resolubilizing in water the crude peptide obtained from cleavage from the solid support, obtaining pyridazinium linkages of type **I** with hydrazine or pyrrole linkages of type **II** when other nucleophiles lacking a second nucleophilic nitrogen were used (Figure 1).

**Figure 1:**
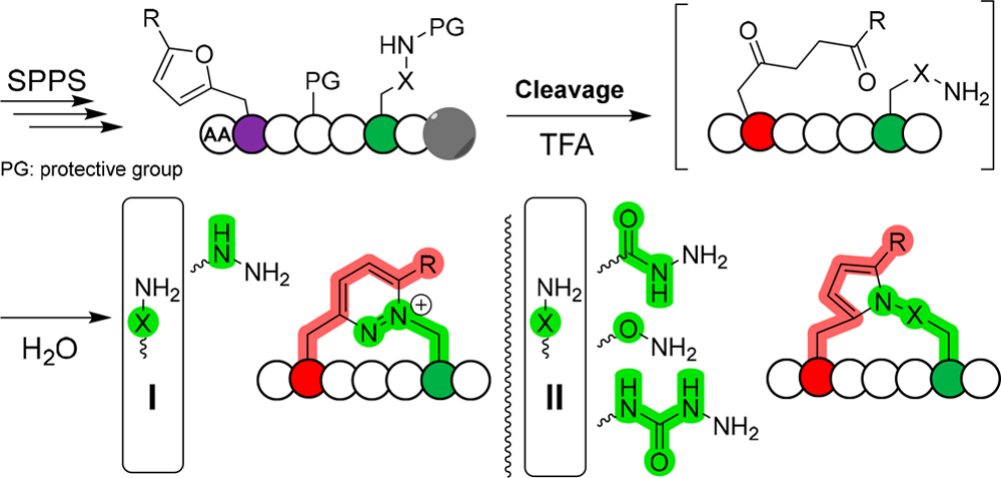
General scheme of the envisaged peptide macrocyclization.

Considering the limited data available pertaining to this reaction (only one electrophile, two nucleophiles, and one pH value were tested), we evaluated the full scope of the reaction by testing additional electrophiles, nucleophiles, and pH conditions in order to better define the conditions required for peptide macrocyclization. First, we explored the possibility of exploiting other (substituted) furans for the generation of 1,4-diketones (Figure 2a) and evaluated the influence of different parameters on the reaction outcome using the coiled-coil peptide-based architecture previously employed to provide the required proximity to the reactive groups (Figure 2b).^24^

**Figure 2:**
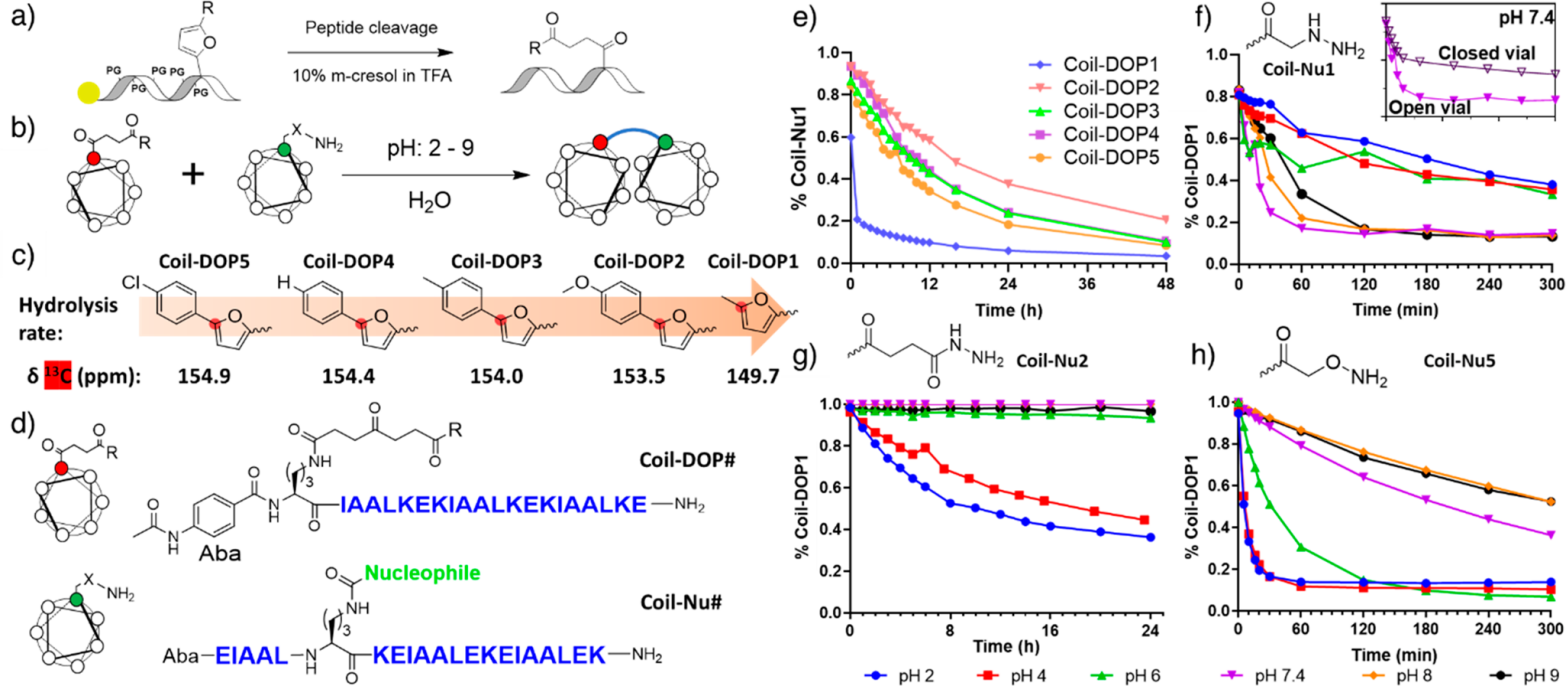
(a,b) Scheme of furan hydrolysis (a) and proximity-induced ligation of coiled peptides (b). (c) Rate order for the hydrolysis of different furan derivatives. (d) Sequences of the different coil peptides. (e) Consumption profiles of **Coil-Nu1** at pH 7.4 in the presence of 1.1 equiv of different electrophile-containing coils. Consumption profiles of **Coil-DOP1** at different pH values, in the presence of 1.1 equiv of different nucleophile-containing coils: (f) **Coil-Nu1** in open vial; (g) **Coil-Nu2**; (h) **Coil-Nu5**.

A series of commercially available 5-aryl-furans were installed on an ornithine side chain, replacing the glutamic acid residue at position 6 of the first heptad of an E-rich coil peptide. Acid-induced cleavage of the peptides from the solid support resulted in a retro-Paal-Knorr reaction of the different derivatives to the desired 1,4-diketones. Hydrolysis kinetics was proportional to the electron density of the aromatic system, as evidenced by faster hydrolysis rates for derivatives with lower chemical shift of C5 for the furan ring (i.e., higher electron density, Figures 2c, S2–S3). The obtained DOP-E-coils were then allowed to react over the weekend with K-rich coil peptides bearing a hydrazine functionality (i.e., via aminoglycine-modified ornithine, replacing a lysine residue). In all cases, the reaction was observed only when hydrazine and the 1,4-diones were located at the same side of the supramolecular coiled-coil system, essential to induce the required proximity. Analysis of the reaction revealed slower kinetics for aryl-containing systems as compared to the methyl-DOP derivative, with nucleophile-consumption rates that inversely correlate with the electron density of the aromatic substituent (Figures 2e, S10). Similar results were obtained using hydrazine-modified R-rich coil peptides (**Coil-Nu1 (R)** and **Coil-MM (R)**, see Figures S4–S8). All ligation products were characterized by HPLC-MS (Figure S9).

Later, we synthesized a series of K-coils bearing different α-effect nucleophiles and monitored their conversion to a stable product in the presence of the DOP-coil under different pH conditions. Interestingly, with hydrazine-containing probes, a faster reaction is observed under neutral conditions with a strong dependence on whether the reaction is performed in an open or closed vial. This dependence is linked to the necessary mild oxidative conditions required for the generation of the final pyridazinium linkage (Figures 2f, S11).^24^ The lack of a second nucleophilic nitrogen on the nucleophile leads to the formation of pyrrole linkages. The formation of this moiety follows a Paal-Knorr-like mechanism, and as expected, faster conversion was observed under acidic conditions (Figures 2g,h, S11). As opposed to hydrazides and semicarbazides, which showed a slow reaction, the aminooxy-containing peptide showed fast consumption, reaching 40% consumption after 5 h under basic conditions or a plateau in less than 30 min under acidic conditions. The structure of this ligation product was confirmed by using representative small molecules (Figures S16–17). These results are in line with previous observations.^24^ Single time point analysis (24 h) of the ligation between the nucleophiles and the other 1,4-diones revealed similar pH dependence (data not shown). All ligation products were confirmed by HPLC-MS (Figures S12–S15).

Once the conditions required for both the hydrolysis of the furan derivatives and a smooth formation of the desired linkages were understood, we applied the reaction to peptide macrocyclization. The required electrophile is then generated during the cleavage of the peptide from the solid support (1 h, twice) to then allow formation of the macrocycle by simply redissolving the crude (for systems bearing hydrazide, semicarbazide, or aminooxy functions, pH ≈ 2) or after adjusting the pH to values around 7.5 (for systems bearing a hydrazine). At the same time, the need for proximity between the reacting functionalities, essential to promoting the reaction, prevents the formation of oligomeric systems when solubilizing the peptides in the low mM range.

To first test the approach, two different single coil peptides bearing two hydrazine functions at different distances from the DOP modification were synthesized (Figure 3a). After cleavage from the solid support, the crude peptides were dissolved in mQ water at 5 mM concentration, and the pH of the solution adjusted to 7.5. After 3 h of reaction, MALDI analysis revealed complete conversion of the starting materials without formation of oligomeric compounds (Figure S19). No differences were observed after the overnight reaction, confirming the possibility to exploit this type of chemistry for the easy and fast production of macrocyclic peptides. In addition, as evidenced by CD and coiled-coil ligation experiments, the macrocyclization did not perturb the peptide secondary structure and occurred selectively between the DOP function and the closest hydrazine, allowing the other hydrazine function to still react subsequently in a proximity-induced ligation (Figures 3b, S20–S21).^25^

**Figure 3:**
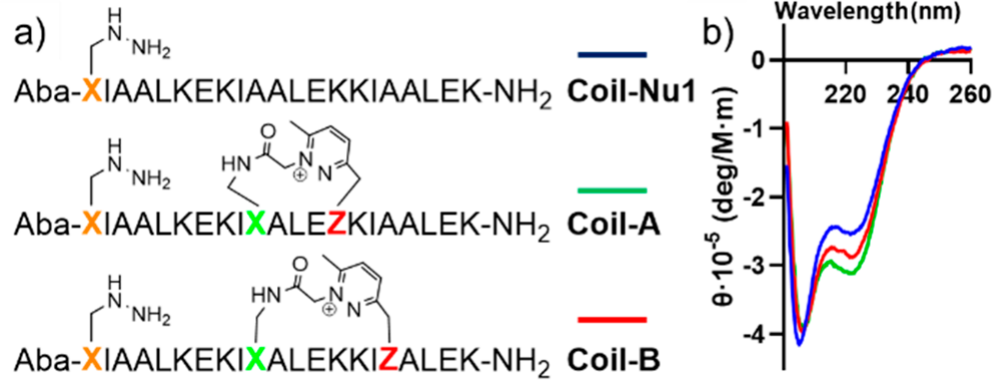
Structure of cyclized coil peptides (a) and their CD signal (b).

Next, exploiting a G4-binder peptide derived from the bovine RHAU helicase, for which it has been reported that residues can be modified for the macrocyclization within the α-coil segment,^26^ we evaluated the formation of cyclized probes using different nucleophiles (i.e., hydrazine, hydrazide, aminooxy) and electrophiles (i.e., methyl- and 4-methoxyphenyl-DOP, Figure 4a). All peptides showed similar reaction outcomes, regardless of the relative position of the two reactive functions (*i*,*i*+4 or *i*,*i*+7). As evidenced by MALDI, peptides containing a hydrazine function quickly reacted after solubilization of the crude peptide in water (pH ≈ 2.5), with complete conversion of the starting material after 1 h of reaction at pH 7.5. Peptides containing either hydrazides or aminooxy functions were fully converted after solubilization in water. As expected, when using an aryl-1,4-dione derivative, the rate of the cyclization reaction was strongly affected, and starting material was still present after 5 days of reaction at pH 7.5 (Figure S23). After purification, all cyclized peptides showed no chemical exchange within 24 h in the presence of 20% acetone (Figures S25–S29) and improved stability toward trypsin degradation (Figures 4b, S30). CD experiments showed increased helical structure for *i*,*i*+4 circular peptides, as evidenced by the increased negative band at 222 nm (Figures 4c, S24). In line with earlier reports,^26^ the introduction of a staple in the peptide decreases the stability of the complex with target MYC G4 DNA under standard G4 forming conditions (10 mM TRIS pH 7.4, 100 mM K^+^) with higher temperature hysteresis. The stability of the complex is, however, less affected under more challenging conditions (addition of 500 mM Li^+^), where **bRHAU-7** showed melting temperatures comparable to linear **bRHAU** (Figures 4d, S31).

**Figure 4:**
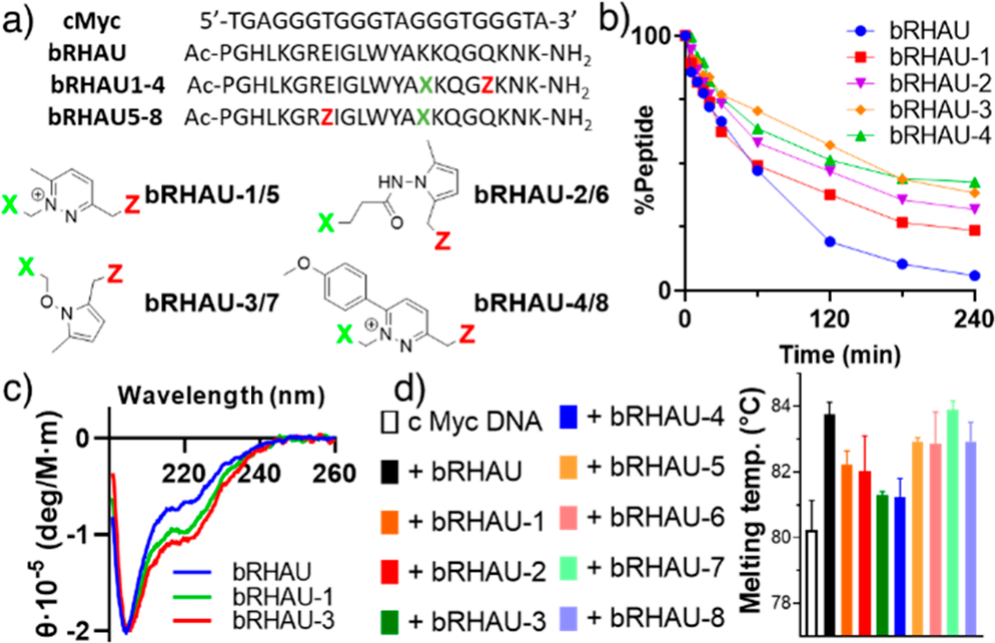
(a) Sequence and linkage structure of cyclized G4-binder peptides. (b) Peptide stability in the presence of trypsin. (c) CD signal of **bRHAU**, **bRHAU-1**, and **bRHAU-3**. (d) Thermal stability of c-Kit DNA in the presence of the different peptides.

Finally, to evaluate the possibility to extend this chemistry to the macrocyclization of unstructured peptides, we selected an RGD peptide analogue and tested the same five combinations of mutually reactive functionalities (Figure 5a). As compared to previous cases where preorganization allowed a controlled distance and orientation, the relative distance of the two reactive units is playing a role in the rate of the reaction, with *i*,*i*+4 systems showing faster conversion as compared to *i*,*i*+7 systems. In this model, hydrazine-containing probes showed faster conversion than in previous cases, using both alkyl- and aryl-1,4-dione derivatives, while hydrazide-containing systems showed slower reaction rates (Figure S33). No significant variations were observed in the CD spectra (data not shown). As opposed to the **bRHAU** case, only cyclized peptides obtained from hydrazine or aminooxy functions showed improved trypsin and serum stability (Figures 5b,c, S33–S34), and these were further investigated in cell-based functional assays.

**Figure 5:**
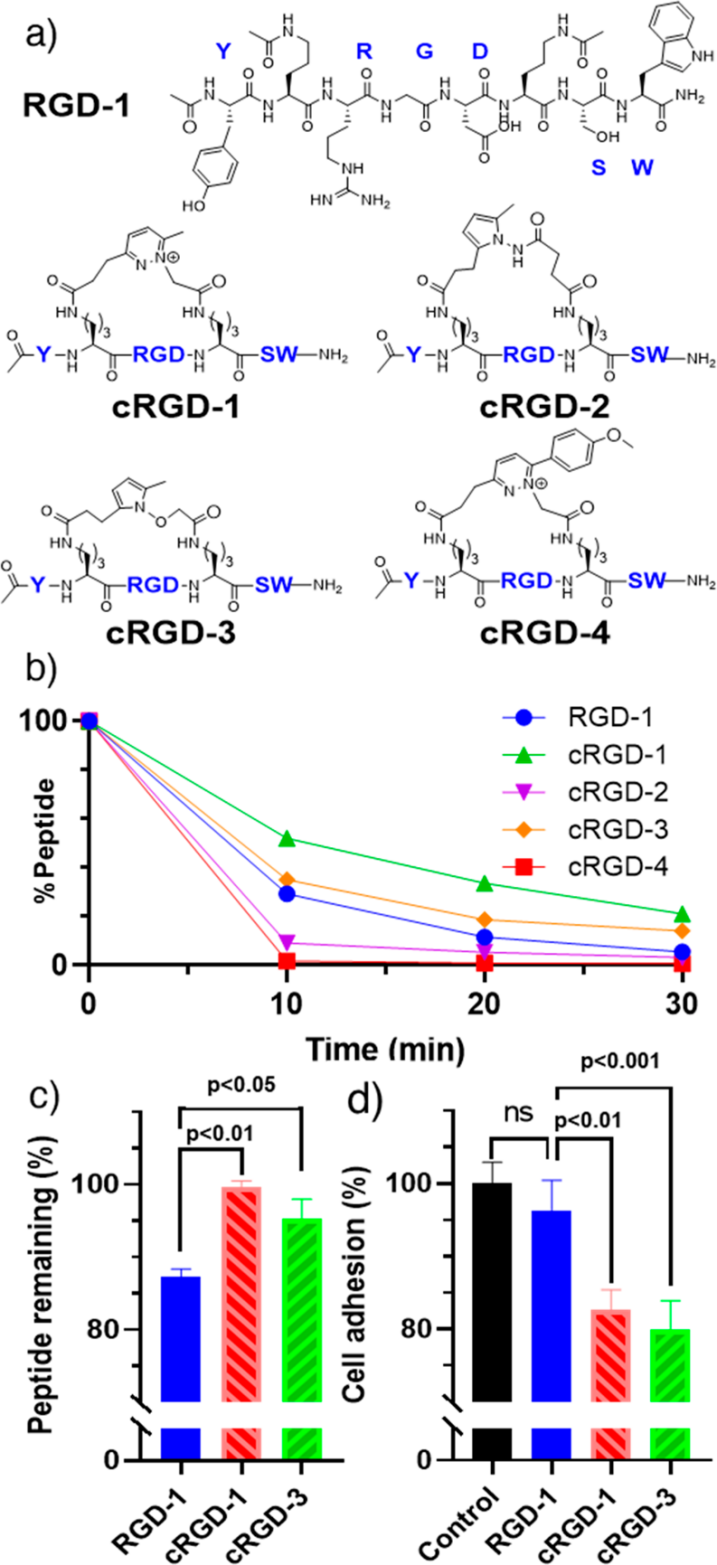
Linear and cyclic RGD peptides (a), their stability toward trypsin degradation (b) and stability in DMEM + 10% serum (FBS) (c), and adhesion assay data performed in DMEM + 10% serum (FBS) at 60 μM peptide for 45 min (d).

RGD-dependent integrin binding to, e.g., fibronectin is an important basis of cell-matrix adhesion.^27^ We compared the functionality of reference and cyclic RGD peptides by measuring the capacity of these peptides to compete with a fibronectin-coated surface and reduce the adhesion of human HeLa cells.^28^ In cell culture medium without serum, all tested peptides showed similar reduction of adhesion to fibronectin (Figure S35) indicating that the cyclization does not affect binding of the RGD peptide to its physiological target protein. However, in the presence of 10% fetal bovine serum, the biological activity is only observed for cyclic peptides, in line with their higher stability in serum (Figures 5d, S36–S39). Together, this indicates that the cyclized peptides obtained using our macrocyclization methods retain their biological activity combined with higher biological stability, opening avenues for further application.

In conclusion, we showed that 1,4-diones can react with different α-nucleophiles without additional triggers to form a stable ligation product. This proximity-induced chemistry was further applied to peptide macrocyclization directly after resolubilization of the crude peptide obtained from resin cleavage, under additive-free conditions, and without special precautions. Cyclization occurred in all peptide models tested, and products proved to have improved biological stability while maintaining the properties of their respective linear analogues. The ease of incorporation and the stability of the pro-electrophile and of the nucleophiles under standard solid-phase peptide synthesis, the possibility of unleashing the required electrophile during standard peptide cleavage conditions, the stability of the formed linkage, and the orthogonality to other macrocyclization techniques can foster the application of this approach for peptide (multi)macrocyclization. Extension of this chemistry to other nucleophiles and the possibility of decorating the furan 5-position with functional moieties (e.g., fluorophores) are currently under investigation.

## Data Availability

The data underlying this study are available in the published article and in its Supporting Information.

## Supporting Information Available

Experimental protocols, evaluation of reaction selectivity and kinetics, full peptide structures, product stability, circular dichroism experiments, melting temperatures, cellular data, and peptide characterizations (PDF)
