# Supplementary material for: Proximity-Induced Ligation and One-Pot Macrocyclization of 1,4-Diketone-Tagged Peptides Derived from 2,5-Disubstituted Furans upon Release from the Solid Support
Source: Org Lett. 2023 Sep 1;25(36):6618–22. doi: 10.1021/acs.orglett.3c02289 (PMC10510716; doi:10.1021/acs.orglett.3c02289)
Supplement: Supplementary file 1 — ol3c02289_si_001.pdf [file ol3c02289_si_001.pdf]

Proximity-Induced Ligation and One-Pot Macrocyclization of 1,4-Diketone-Tagged Peptides Derived from 2,5-Disubstituted Furans upon Release from the Solid Support

Alex Manicardi,[a,b‡*] Atiruj Theppawong,[b‡] Marleen Van Troys,[c] Annemieke Madder[b]

[a] Department of Chemistry, Life Sciences and Environmental Sustainability, University of Parma, Parco Area delle Scienze 17/A, 43124 Parma, Italy

[b] Organic and Biomimetic Chemistry Research Group, Department of Organic and Macromolecular Chemistry, Ghent University, Krijgslaan 281-S4, 9000 Ghent, Belgium.

[c] Department of Biomolecular Medicine, Ghent University, Technologiepark-Zwijnaarde 75, 9052 Ghent, Belgium

Supplemental Information

Table of contents

1. General information 2
   1.1 Peptide synthesis 3
   1.2 Peptide cyclization 3
   1.3 Peptide characterization 3
2. Effect of furan substitution on ring hydrolysis 6
3. Ligation and selectivity validation 8
4. Evaluation of reaction kinetics 12
5. Structural investigation on ligation with aminooxy moieties 16
6. Cyclization – Coiled peptide 18
7. Cyclization – Bovine RHAU (G4 binder) 20
   7.1 Peptide macrocycle chemical stability 22
   7.2 Trypsin stability 26
   7.3 Thermal denaturation experiments 27
8. Cyclization – RGD peptide 28
   8.1 Trypsin stability 30
   8.2 Serum stability 30
   8.1 Cell adhesion assay 30
9. References 33
10. HPLC-MS chromatograms of purified peptides 34

1. General information

All reagents were purchased from Sigma-Aldrich, Fluka, Merck, TCI Europe, Iris Biotech, Enamine, and used without further purification. Dry DMF was stored over 4 Å molecular sieves.
NMR spectra were recorded on a Brucker Avance 400. δ values are expressed in ppm relatively either to $CDCl_3$ (7.29 ppm for proton and 76.9 ppm for carbon) or DMSO-d^6 (2.50 ppm for proton and 39.5 ppm for carbon). The following abbreviations are used to explain the multiplicities: s=singlet, d=doublet, t=triplet, q=quartet, m=multiplet, and br=broad.
HPLC-MS data were collected on an Agilent 1100 Series instrument equipped with a Phenomenex Kinetex C18 100 Å column (150 x 4.6 mm, 5 µm at 35 °C) connected to an ESMSD type VL mass detector (quadrupole ion trap mass spectrometer) with a flow rate of 1.5 mL/min was used with the following solvent systems: (A): 0.1% HCOOH in H_2O and (B) MeCN. Gradient: 100% A for 2 min, then a gradient from 0 to 100% B over 6 min was used, followed by 2 min of flushing with 100% B (HPLC1).
HPLC-UV data were collected on an Agilent 1100 Series instrument equipped with a Waters XTERRA RP18 5µm column (250 x 2.1 mm at 40°C) connected to a DAD using a flow rate of 0.35 mL/min with the following solvent systems: (A): 0.1% TFA in H_2O and (B) 0.1% TFA in MeCN. Gradient: 100% A for 1 min, then a gradient from 0 to 100% B in 13 min, followed by 5 min of flushing with 100% B (HPLC1); 100% A for 1 min, then a gradient from 0 to 30% B in 1 min, then to 60% B in 10 min, and finally to 100% B in 1 min, followed by 3.5 min of flushing with 100% B (HPLC2). Peptides were purified using a Phenomenex Luna C18(2) (5 µm, 100 Å, 250x4.6 mm) (HPLC3, 100% A for 1 min, then a gradient from 0 to 100% B over 30 min at a flow rate of 4.0 mL/min) or using a Phenomenex Luna C18(2) (5 µm, 250 Å, 250x21.2 mm) on an Agilent 218 solvent delivery system (HPLC4, 100% A for 2 min, then a gradient to 100% in 30 min at a flow rate of 17.5 mL/min). Fast analyses were conducted on an Agilent 1260 infinity II equipped with a Chromolith® High resolution, 2 µm column (RP-18e, 50x4.6 mm at 35°C) using a flow rate of 3.0 mL/min: 100% A for 0.5 min, then a gradient from 0 to 100% B in 5 min, followed by 0.5 min of flushing with 100% B (HPLC5).
UV-VIS spectra were recorded using a Trinean DropSense96 UV/VIS droplet reader.
Buffer: unless otherwise mentioned, reactions were carried out in 10 mM phosphate, 100 mM NaCl pH7.4 buffer (PBS), or in 1/10 Borate/Citrate/Phosphate buffer[1] containing 100 mM NaCl (BCPS) for other pH values.
CD spetra were recorded using a Jasco J1500 spectropolarimeter equipped with a thermostated cell holder at 5 µM peptide concentration. Spectra were collected as an average of 5 scans recorded at 50 nm/min scan speed in continuous mode.
Synthesis of N6-Boc-(hydrazinecarbonyl)glycine, 4-(2-Boc-hydrazinecarbonyl)benzoic acid, and 4-(2-Boc-hydrazineyl)-4-oxobutanoic acid was previously described in Manicardi *et al.* 2020[2]; synthesis of **Coil-Nu1**, **Coil-Nu1(R)**, **Coil-MM**, **Coil-MM(R)**, and **Coil-DOP1** was previously described in Manicardi *et al.* 2021[3]

1.1 Peptide synthesis

All peptides were synthesized on a 5 μmol scale. The synthesis of the peptide probes was performed under standard automatic Fmoc-based solid-phase synthesis conditions using a Syro automatic peptide synthesizer, using HBTU/DIPEA as coupling mixture and Fmoc-$Orn_{(Mtt)}$-OH and Fmoc-$Orn_{(Dde)}$-OH as well as standard Fmoc-protected amino acids. Rinkamide-AM Champion resin was directly loaded in the synthesis reactor (0.69 mmol/g). Modification of the ornithine side chain was performed after azide reduction (1M PMe_3 in THF/THF/H_2O 1:2:3, 2x10 minutes, vigorous stirring),[4] Mtt (0.5% BtOH·H_2O in HFIP/DCM 1:1, 4x4 minutes; Mtt deprotection can be visually followed by adding 1 drop of TFA to the deprotection solution except when $His_{(Trt)}$ is present)[5] or Dde (250 mg hydroxylamine hydrochloride, 184 mg imidazole, 1 mL NMP, 200 μL DMF, 1x2h)[6] removal for furan-precursors or protected α-effect nucleophiles coupling. All coupling steps were performed using HBTU/DIPEA as activating mixture except for 4-(2-Boc-hydrazineyl)-4-oxobutanoic acid were a DIC/DhBtOH activating mixture was used. Cleavage was performed using a TFA/m-cresol 9:1 cleavage cocktail (2x 1h). Peptides containing aminooxy modifications were dissolved in a water solution containing 2.5-3.0% O-methylhydroxylamine hydrochloride and allowed to react for at least 3h prior to purification. After RP-HPLC purification (HPLC4), the purity and identity of the peptides were evaluated by LC-MS (HPLC1). Probe concentration was evaluated by measuring the absorbance at 270 nm (unless otherwise mentioned), using Lambert-Beer's law. All peptides appeared as amorphous white solids.

1.2 Peptide cyclization

After desired side-chain modification (*vide supra*), peptides were cleaved from the solid supports using a TFA/*m*-cresol 9:1 solution (2x 1h) and precipitated in diethyl ether. Crude was dissolved at 1 or 5 mM concentration in mQ water (pH ~2.5), pH is adjusted to ~7.4 for a system containing hydrazine, and allowed to react for 2-24h. Reaction competition was monitored via MALDI-TOF analysis.

1.3 Peptide characterization

Table S1: peptide sequences employed in this study. Capital letters indicate L-amino acids, modifications on the ornithine side chains are inserted inside brackets. ABA: 4-Acetamidobenzoyl. * indicates linked residues.

Name	Sequence	MW
Coil-Nu1	ABA-Orn(Nu1)-IAALKEKIAALKEKIAALKE-NH_2	2498.0
Coil-MM	ABA-KIAAL-Orn(Nu1)-EKIAALKEKIAALKE-NH_2	2498.0
Coil-Nu1 (R)	ABA-Orn(Nu1)-IAALRERIAALRERIAALRE-NH_2	2638.1
Coil-MM (R)	ABA-RIAAL-Orn(Nu1)-ERIAALRERIAALRE-NH_2	2638.1
Coil-Nu2	ABA-Orn(Nu2)-IAALKEKIAALKEKIAALKE-NH_2	2540.0
Coil-Nu3	ABA-Orn(Nu3)-IAALKEKIAALKEKIAALKE-NH_2	2541.0
Coil-Nu4	ABA-Orn(Nu4)-IAALKEKIAALKEKIAALKE-NH_2	2588.1
Coil-Nu5	ABA-Orn(Nu5)-IAALKEKIAALKEKIAALKE-NH_2	2499.0
Coil-DOP1	ABA-EIAAL-Orn(DOP1)-KEIAALEKEIAALEK-NH_2	2583.0
Coil-DOP2	ABA-EIAAL-Orn(DOP2)-KEIAALEKEIAALEK-NH_2	2674.1
Coil-DOP3	ABA-EIAAL-Orn(DOP3)-KEIAALEKEIAALEK-NH_2	2658.1
Coil-DOP4	ABA-EIAAL-Orn(DOP4)-KEIAALEKEIAALEK-NH2	2644.0
Coil-DOP5	ABA-EIAAL-Orn(DOP5)-KEIAALEKEIAALEK-NH2	2678.5
Coil-DOP6	ABA-EIAAL-Orn(DOP6)-KEIAALEKEIAALEK-NH_2	2596.0
Coil-A	ABA-Orn(Nu1)-IAALKEKI-Orn(Nu1*)-ALK-Orn(DOP1*)-KIAALKE-NH_2	2716.4
Coil-B	ABA-Orn(Nu1)-IAALKEKI-Orn(Nu1*)-ALKEKI-Orn(DOP1*)-ALKE-NH_2	2774.4
bRHAU	Ac-PGHLKGREIGLWYAKKQGQKNK-NH_2	2578.0
bRHAU-1	Ac-PGHLKGREIGLWYA-Orn(Nu1*)-KQG-Orn(DOP1*)-KNK-NH_2	2739.2
bRHAU-2	Ac-PGHLKGREIGLWYA-Orn(Nu2*)-KQG-Orn(DOP1*)-KNK-NH_2	2782.3
bRHAU-3	Ac-PGHLKGREIGLWYA-Orn(Nu5*)-KQG-Orn(DOP1*)-KNK-NH_2	2741.2
bRHAU-4	Ac-PGHLKGREIGLWYA-Orn(Nu1*)-KQG-Orn(DOP2*)-KNK-NH_2	2830.3
bRHAU-5	Ac-PGHLKGR-Orn(DOP1*)-IGLWYA-Orn(Nu1*)-KQGQKNK-NH_2	2738.2
bRHAU-6	Ac-PGHLKGR-Orn(DOP1*)-IGLWYA-Orn(Nu2*)-KQGQKNK-NH_2	2781.3
bRHAU-7	Ac-PGHLKGR-Orn(DOP1*)-IGLWYA-Orn(Nu5*)-KQGQKNK-NH_2	2740.2
bRHAU-8	Ac-PGHLKGR-Orn(DOP2*)-IGLWYA-Orn(Nu1*)-KQGQKNK-NH_2	2829.4

RGD-1	Ac-Y-Orn$_{(Ac)}$-RGD-Orn$_{(Ac)}$-SW-NH_2	1135.5
RGD-2	Ac-Orn$_{(Ac)}$-WRGDYS-Orn$_{(Ac)}$-NH_2	1135.5
cRGD-1	Ac-Y-Orn(DOP1*)-RGD-Orn(Nu1*)-SW-NH_2	1241.3
cRGD-2	Ac-Y-Orn(DOP1*)-RGD-Orn(Nu2*)-SW-NH_2	1284.4
cRGD-3	Ac-Y-Orn(DOP1*)-RGD-Orn(Nu5*)-SW-NH_2	1243.3
cRGD-4	Ac-Y-Orn(DOP2*)-RGD-Orn(Nu1*)-SW-NH_2	1333.4
cRGD-5	Ac-Orn(DOP1*)-WRGDYS-Orn(Nu1*)-NH_2	1241.3
cRGD-6	Ac-Orn(DOP1*)-WRGDYS-Orn(Nu2*)-NH_2	1284.4
cRGD-7	Ac-Orn(DOP1*)-WRGDYS-Orn(Nu5*)-NH_2	1243.3
cRGD-8	Ac-Orn(DOP2*)-WRGDYS-Orn(Nu1*)-NH_2	1333.4

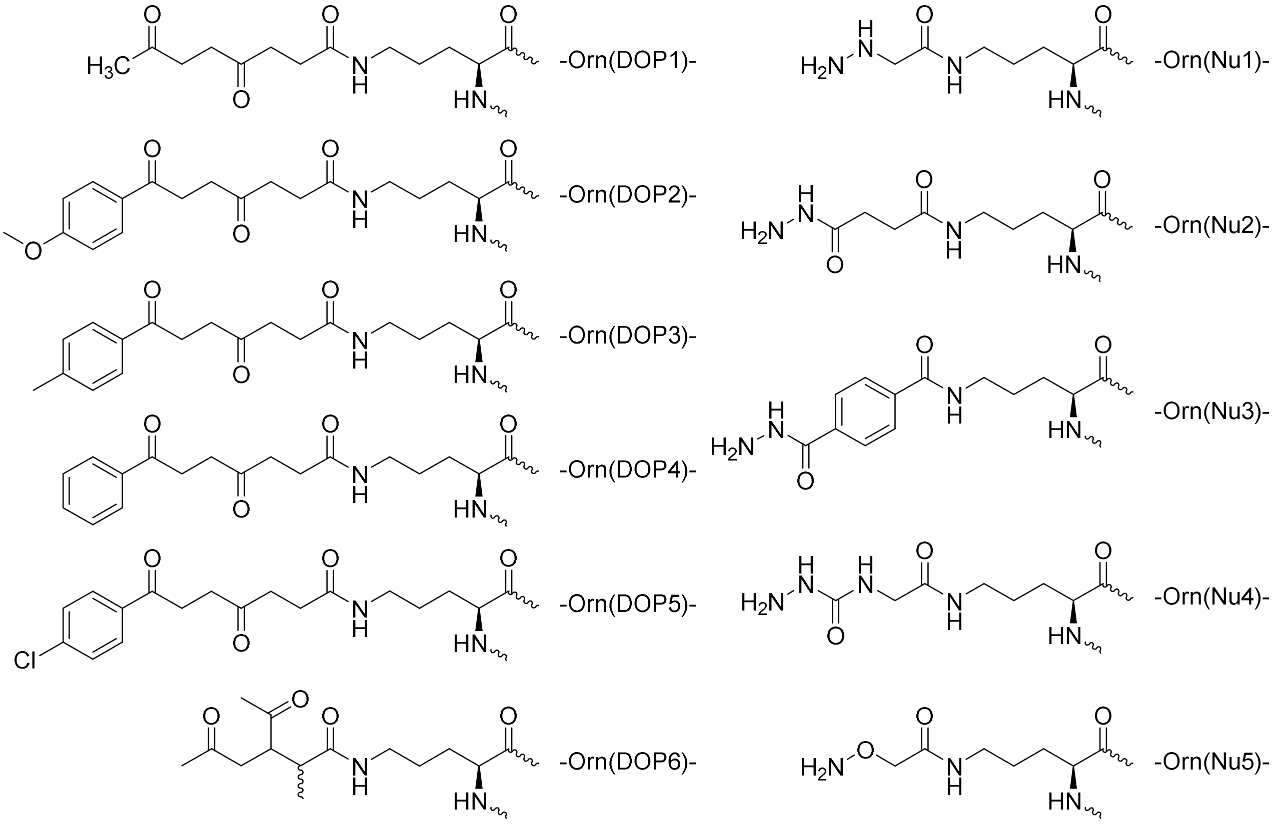

Fig. S1: structures of modified amino acids.

Coil-Nu2: 19.4%; t_r: 3.95 min (HPLC1); ε = 17989 $M^{-1}cm^{-1}$; ESI-MS: *m/z* calcd 2540.0 [M]: 1270.5 $[M+2H]^{2+}$, 847.3 $[M+3H]^{3+}$, 635.8 $[M+4H]^{4+}$, 509.0 $[M+5H]^{5+}$, 424.2 $[M+6H]^{6+}$; **Coil-Nu3:** 12.4%; t_r: 3.95 min (HPLC1); ε = 17989 M-1cm-1; ESI-MS: m/z calcd 2541.0 [M]: 1270.9 [M+2H]2+, 847.7 [M+3H]3+, 636.0 $[M+4H]^{4+}$, 509.1 $[M+5H]^{5+}$, 424.4 $[M+6H]^{6+}$; **Coil-Nu4:** 17.6%; t_r: 3.99 min (HPLC1); ε = 24744 $M^{-1}cm^{-1}$; ESI-MS: *m/z* calcd 2588.1 [M]: 1294.5 $[M+2H]^{2+}$, 863.4 $[M+3H]^{3+}$, 647.8 $[M+4H]^{4+}$, 518.5 $[M+5H]^{5+}$, 432.3 $[M+6H]^{6+}$; **Coil-Nu5:** 12.8%; t_r: 3.98 min (HPLC1); ε = 17989 $M^{-1}cm^{-1}$; ESI-MS: *m/z* calcd 2499.0 [M]: 1249.9 $[M+2H]^{2+}$, 833.7 $[M+3H]^{3+}$, 625.5 $[M+4H]^{4+}$, 500.7 $[M+5H]^{5+}$; **Coil-DOP2:** 22.6%; t_r: 4.72 min (HPLC1); ε = 17989 $M^{-1}cm^{-1}$; ESI-MS: *m/z* calcd 2644.0 [M]: 1337.7 $[M+2H]^{2+}$, 892.0 $[M+3H]^{3+}$, 665.0 $[M+4H]^{4+}$; **Coil-DOP3:** 6.55%; t_r: 4.75 min (HPLC1); ε = 17989 $M^{-1}cm^{-1}$; ESI-MS: *m/z* calcd 2674.1 [M]: 1359.6 $[M+2H]^{2+}$, 886.8 $[M+3H]^{3+}$, 660.9 $[M+4H]^{4+}$; **Coil-DOP4:** 20.8%; t_r: 4.70 min (HPLC1); ε = 17989 $M^{-1}cm^{-1}$; ESI-MS: *m/z* calcd 2596.0 [M]: 1322.7 $[M+2H]^{2+}$, 882.2 $[M+3H]^{3+}$, 657.3 $[M+4H]^{4+}$; **Coil-DOP5:** 26.4%; t_r: 4.77 min (HPLC1); ε = 17989 $M^{-1}cm^{-1}$; ESI-MS: *m/z* calcd 2678.5 [M]: 1340.1 $[M+2H]^{2+}$, 893.5 $[M+3H]^{3+}$, 666.2 $[M+4H]^{4+}$; **Coil-DOP6:** 8.31% as mixture of epimers; t_r: 4.61-4.66 min (HPLC1); ε = 17989 $M^{-1}cm^{-1}$; ESI-MS: *m/z* calcd 2596.01 [M]: 1298.7 $[M+2H]^{2+}$, 866.1 $[M+3H]^{3+}$, 645.3 $[M+4H]^{4+}$; **Coil-A:** 6.2%; t_r: 3.80 min (HPLC1); ε = 17989 $M^{-1}cm^{-1}$; ESI-MS: *m/z* calcd 2715.39 $[M]^{+}$: 1358.3 $[M+H]^{2+}$, 905.8 $[M+2H]^{3+}$, 679.7 $[M+3H]^{4+}$, 544.0 $[M+4H]^{5+}$, 453.5 $[M+5H]^{6+}$; **Coil-B:** 6.9%; t_r: 3.85 min (HPLC1); ε = 17989 $M^{-1}cm^{-1}$; ESI-MS: *m/z* calcd 2773.43 $[M]^{+}$: 1387.5 $[M+H]^{2+}$, 925.3 $[M+2H]^{3+}$, 694.2 $[M+3H]^{4+}$, 555.6 $[M+4H]^{5+}$, 463.2 $[M+5H]^{6+}$; **bRHAU:** 25.0%; t_r: 3.25 min (HPLC1); ε = 6970 $M^{-1}cm^{-1}$; ESI-MS: *m/z* calcd 2577.98 [M]: 1289.4 $[M+2H]^{2+}$,

860.0 [M+3H]$^{3+}$, 645.3 [M+4H]$^{4+}$, 516.5 [M+5H]$^{5+}$, 430.5 [M+6H]$^{6+}$, 396.1 [M+7H]$^{7+}$; **bRHAU-1:** 18.2%; t_r: 3.40 min (HPLC1); ε = 6970 $M^{-1}cm^{-1}$; ESI-MS: *m/z* calcd 2739.3 [M]$^{+}$: 1370.4 [M+H]$^{2+}$, 914.0 [M+2H]$^{3+}$, 685.8 [M+3H]$^{4+}$, 548.8 [M+4H]$^{5+}$, 457.5 [M+5H]$^{6+}$; **bRHAU-2:** 8.24%; t_r: 3.45 min (HPLC1); ε = 6970 $M^{-1}cm^{-1}$; ESI-MS: *m/z* calcd 2782.3 [M]: 1391.4 [M+2H]$^{2+}$, 927.9 [M+3H]$^{3+}$, 696.2 [M+4H]$^{4+}$, 557.3 [M+5H]$^{5+}$, 464.5 [M+6H]$^{6+}$; **bRHAU-3:** 3.48%; t_r: 3.56 min (HPLC1); ε = 6970 $M^{-1}cm^{-1}$; ESI-MS: *m/z* calcd 2741.2 [M]: 1371.0 [M+2H]$^{2+}$, 914.3 [M+3H]$^{3+}$, 686.0 [M+4H]$^{4+}$, 549.0 [M+5H]$^{5+}$, 457.6 [M+6H]$^{6+}$; **bRHAU-4:** 10.9%; t_r: 3.42 min (HPLC1); ε = 6970 $M^{-1}cm^{-1}$; ESI-MS: *m/z* calcd 2830.3 [M]$^{+}$: 1415.6 [M+H]$^{2+}$, 950.8 [M+2H]$^{3+}$, 708.3 [M+3H]$^{4+}$, 566.8 [M+4H]$^{5+}$, 472.6 [M+5H]$^{6+}$, 405.1 [M+6H]$^{7+}$; **bRHAU-5:** 16.8%; t_r: 3.42 min (HPLC1); ε = 6970 $M^{-1}cm^{-1}$; ESI-MS: *m/z* calcd 2738.2 [M]$^{+}$: 1376.9 [M+H]$^{2+}$, 913.7 [M+2H]$^{3+}$, 685.5 [M+3H]$^{4+}$, 548.7 [M+4H]$^{5+}$, 457.4 [M+5H]$^{6+}$; **bRHAU-6:** 12.6%; t_r: 3.44 min (HPLC1); ε = 6970 $M^{-1}cm^{-1}$; ESI-MS: *m/z* calcd 2781.3 [M]: 1391.1 [M+2H]$^{2+}$, 914.7 [M+3H]$^{3+}$, 695.8 [M+4H]$^{4+}$, 557.0 [M+5H]$^{5+}$, 464.3 [M+6H]$^{6+}$; **bRHAU-7:** 13.9%; t_r: 3.41 min (HPLC1); ε = 6970 $M^{-1}cm^{-1}$; ESI-MS: *m/z* calcd 2740.2 [M]: 1379.6 [M+H_2O+2H]$^{2+}$, 914.1 [M+H_2O+3H]$^{3+}$, 690.3 [M+H_2O+4H]$^{4+}$, 552.5 [M+H_2O+5H]$^{5+}$, 460.5 [M+H_2O+6H]$^{6+}$; **bRHAU-8:** 17.1%; t_r: 3.64 min (HPLC1); ε = 6970 $M^{-1}cm^{-1}$; ESI-MS: *m/z* calcd 2829.4 [M]$^{+}$: 708.2 [M+3H]$^{4+}$, 567.0 [M+4H]$^{5+}$, 472.5 [M+5H]^{6}; **RGD-1:** 17.5%; t_r: 3.67 min (HPLC1); ε = 6970 $M^{-1}cm^{-1}$; ESI-MS: *m/z* calcd 1135.5 [M]: 1136.3 [M+H]$^{+}$, 568.7 [M+2H]$^{2+}$, 379.5 [M+3H]$^{3+}$; **RGD-2:** 33.5%; t_r: 3.62 min (HPLC1); ε = 6970 $M^{-1}cm^{-1}$; ESI-MS: *m/z* calcd 1135.5 [M]: 1136.3 [M+H]$^{+}$, 568.7 [M+2H]$^{2+}$, 379.5 [M+3H]$^{3+}$; **cRGD-1**: 16.8%; tr: 3.81 min (HPLC1); ε = 6970 $M^{-1}cm^{-1}$; ESI-MS: m/z calcd 1241.3 [M]$^{+}$: 1242.3 [M]$^{+}$, 621.8 [M+H]$^{2+}$; **cRGD-2**: 2.3%; tr: 3.94 min (HPLC1); ε = 6970 $M^{-1}cm^{-1}$; ESI-MS: m/z calcd 1284.4 [M]: 1285.2 [M+H]$^{+}$, 642.8 [M+2H]$^{2+}$; **cRGD-3**: 8.1%; tr: 3.90 min (HPLC1); ε = 6970 $M^{-1}cm^{-1}$; ESI-MS: m/z calcd 1243.3 [M]: 1261.2 [M+H_2O+H]$^{+}$, 631.2 [M+H_2O+2H]$^{2+}$; **cRGD-4**: 13.5%; tr: 3.65 min (HPLC1); ε = 6970 $M^{-1}cm^{-1}$; ESI-MS: m/z calcd 1333.4 [M]$^{+}$: 1334.3 [M+H]$^{+}$, 666.8 [M+2H]$^{2+}$, 444.9 [M+2H]$^{3+}$; **cRGD-5**: 16.7%; tr: 3.78 min (HPLC1); ε = 6970 $M^{-1}cm^{-1}$; ESI-MS: m/z calcd 1241.3 [M]$^{+}$: 1242.3 [M]$^{+}$, 621.8 [M+H]$^{2+}$;

cRGD-6: 4.3%; tr: 3.85 min (HPLC1); ε = 6970 $M^{-1}cm^{-1}$; ESI-MS: m/z calcd 1284.4 [M]: 1285.2 [M+H]$^{+}$, 642.8 [M+2H]$^{2+}$; **cRGD-7**: 5.2%; tr: 3. 3.49 min (HPLC1); ε = 6970 $M^{-1}cm^{-1}$; ESI-MS: m/z calcd 1243.3 [M]: 1262.3 [M+H_2O+H]$^{+}$, 622.2 [M+2H]$^{2+}$; **cRGD-8**: 13.9%; tr: 3.59 min (HPLC1); ε = 6970 $M^{-1}cm^{-1}$; ESI-MS: m/z calcd 1333.4 [M]$^{+}$: 1334.3 [M+H]$^{+}$, 666.8 [M+2H]$^{2+}$, 444.9 [M+2H]$^{3+}$.

2. Effect of furan substitution on ring hydrolysis

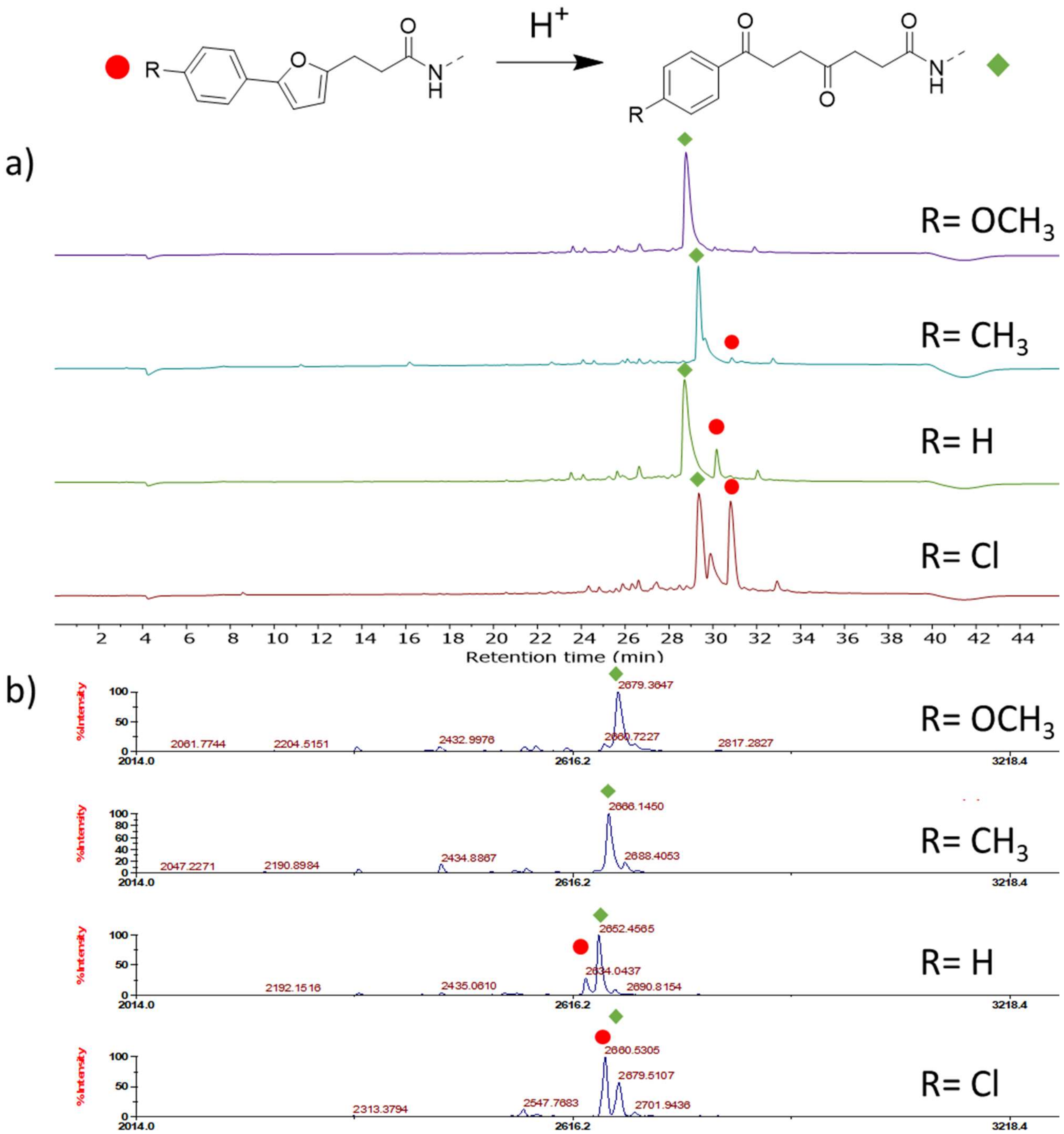

Fig. S2: crude peptides obtained after 1h cleavage in presence of 10% m-cresol in TFA. (a) HPLC-UV traces; (b) MALDI-TOF spectra. For clarity, peaks corresponding to closed furan system are marked with red circles and peaks corresponding to 1,4-diketones are marked with green diamonds. For chromatographic conditions, please refer to the general information section.

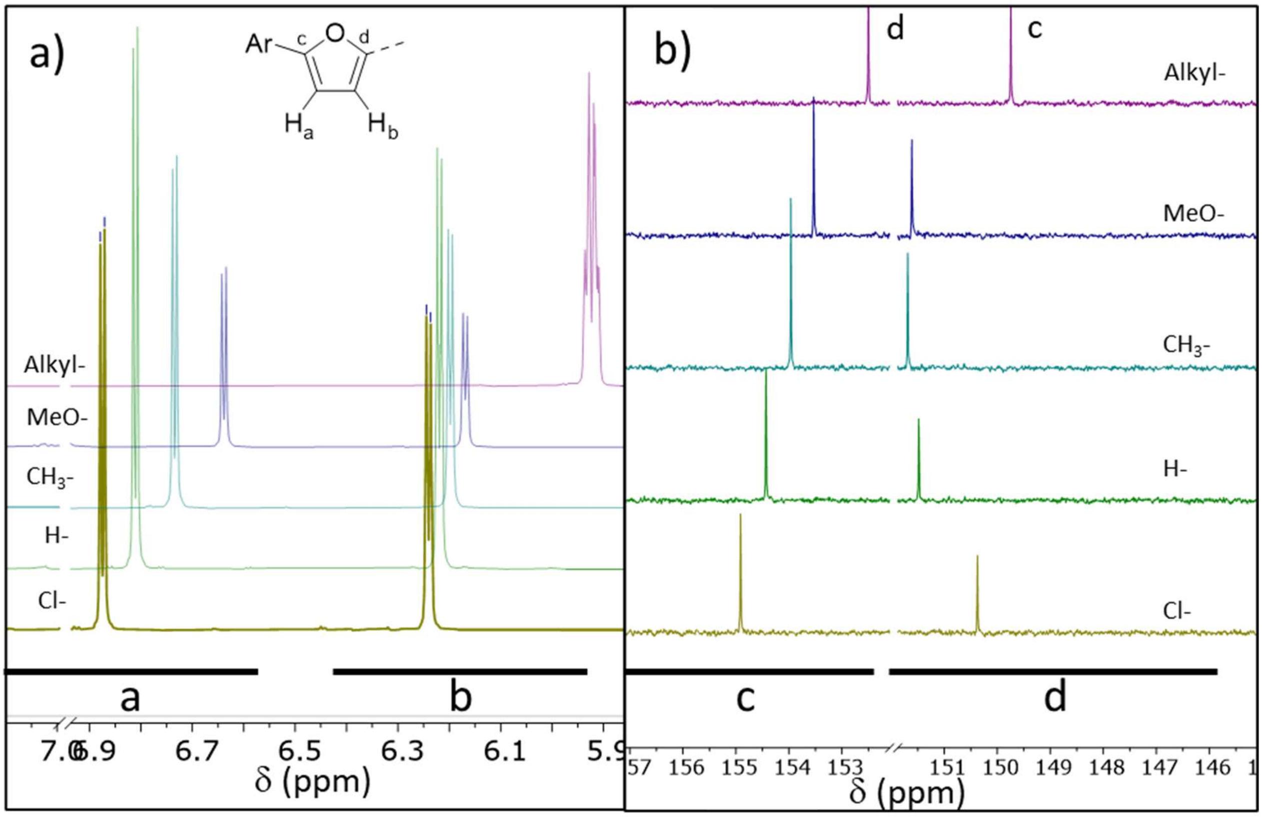

Fig. S3: ^{1}H-NMR (a) and ^{13}C-NMR (b) chemical shifts of the 2,5-dimodified furans employed in this study. As it can be noted, the increased deshielding of H_a, H_b, and C_c correlate with a lower efficiency of furan ring hydrolysis as reported in Fig. S2. 'Alkyl' denotes 3-(5-methylfuran-2-yl)propionic acid

3. Ligation and selectivity validation

In a typical experiment, 100 µL of buffered solution (PBS pH 7.4) containing probes at 5 µM concentration (from a 200 µM stock solution), were prepared in a 1.5 mL Eppendorf and allowed to react over the weekend at 25°C. The solutions were then collected, quenched with acetone, and analyzed via HPLC-UV and HPLC-MS.

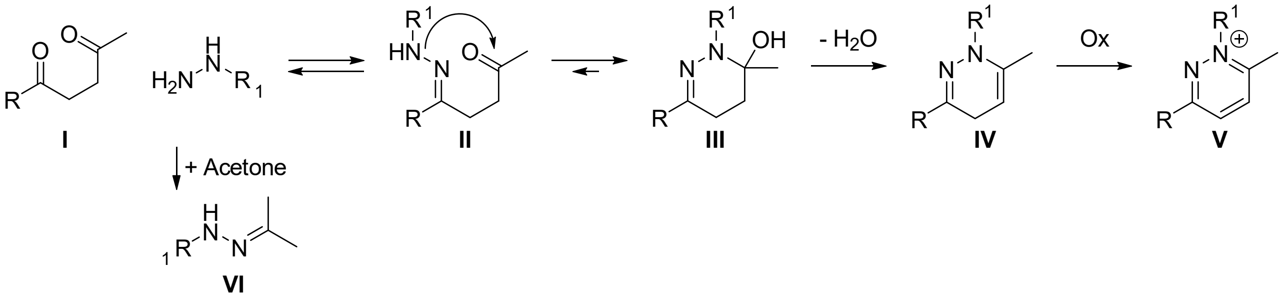

Scheme S1: proposed mechanism of formation of ligation/macrocyclization product with hydrazine. After the attachment of the hydrazine on the DOP derivative (**I**), a hydrazone intermediate is generated (**II**), which then can react with the second nucleophilic nitrogen to form a cyclic intermediate (**III**), which could undergo dehydration. Finally, intermediate (**IV**) can be oxidized by dissolved oxygen to the final ligation/macrocyclization product (**V**). Addition of excess acetone to the reaction mixture will irreversibly trap the hydrazine-probe engaging the formation of an unreactive hydrazone product (**VI**), inducing the dissociation of all intermediates that are in equilibrium with the starting material. In ligation experiments, the formation of different peaks could be observed in HPLC experiments. This is attributed to the formation of stable intermediates that do not revert to starting material after acetone quenching.

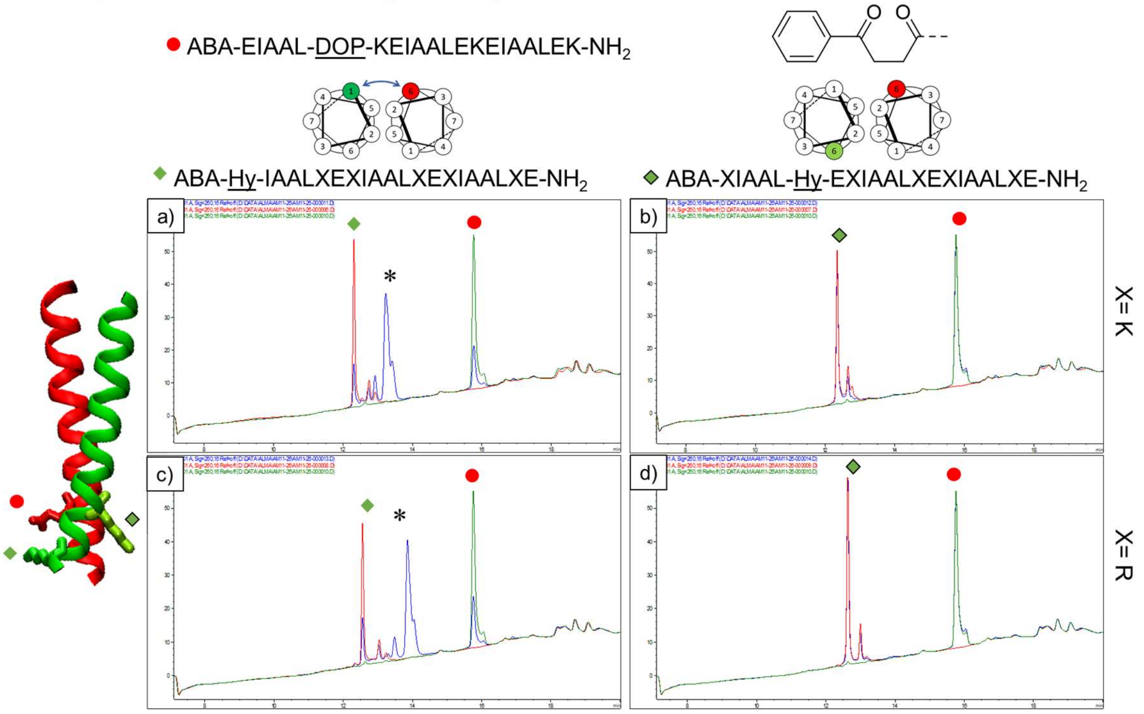

Fig. S4: HPLC1 traces of the ligation experiments performed in presence of **Coil-DOP3** (red dot): (a) with **Coil-Nu1**, (b) with **Coil-Nu1(R)**, (c) with **Coil-MM**, (d) with **Coil-MM(R)**. DOP: DOP-modified ornithine; Hy: aminoglycine-modified ornithine; For clarity, the peaks corresponding to the different hydrazine-containing probes are marked with a green diamond; *: ligation products. For chromatographic conditions, please refer to the general information section.

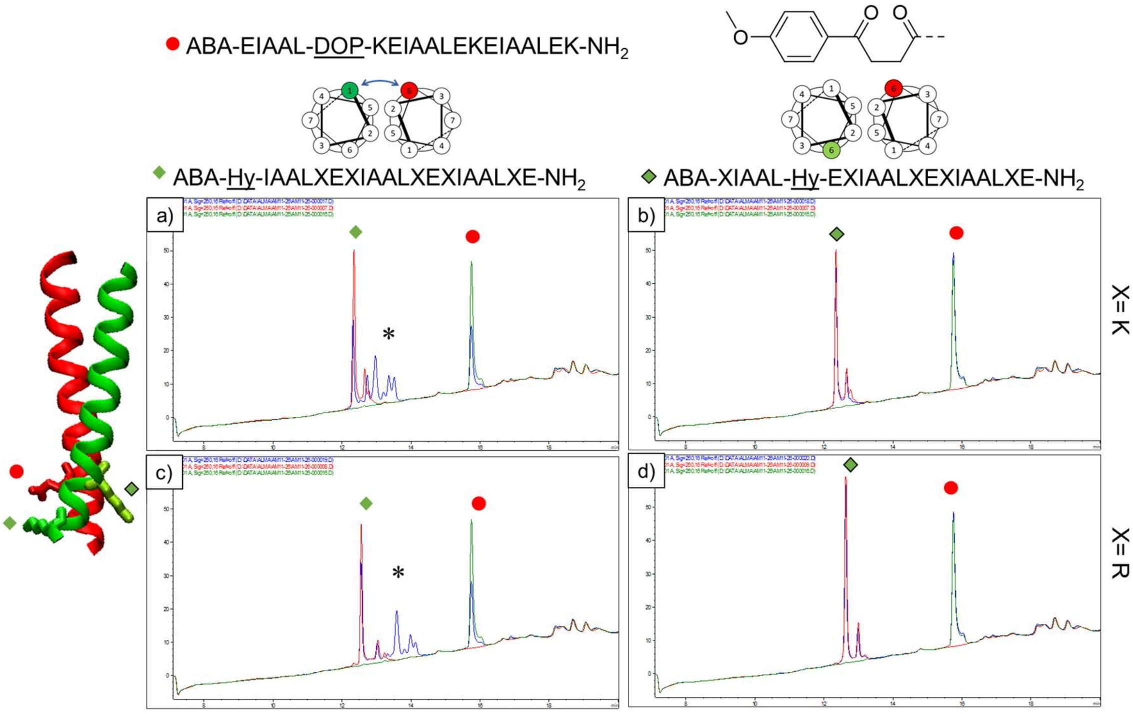

Fig. S5: HPLC1 traces of the ligation experiments performed in presence of **Coil-DOP2** (red dot): (a) with **Coil-Nu1**, (b) with **Coil-Nu1(R)**, (c) with **Coil-MM**, (d) with **Coil-MM(R)**. DOP: DOP-modified ornithine; Hy: aminoglycine-modified ornithine; For clarity, the peaks corresponding to the different hydrazine-containing probes are marked with a green diamond; *: ligation products. For chromatographic conditions, please refer to the general information section.

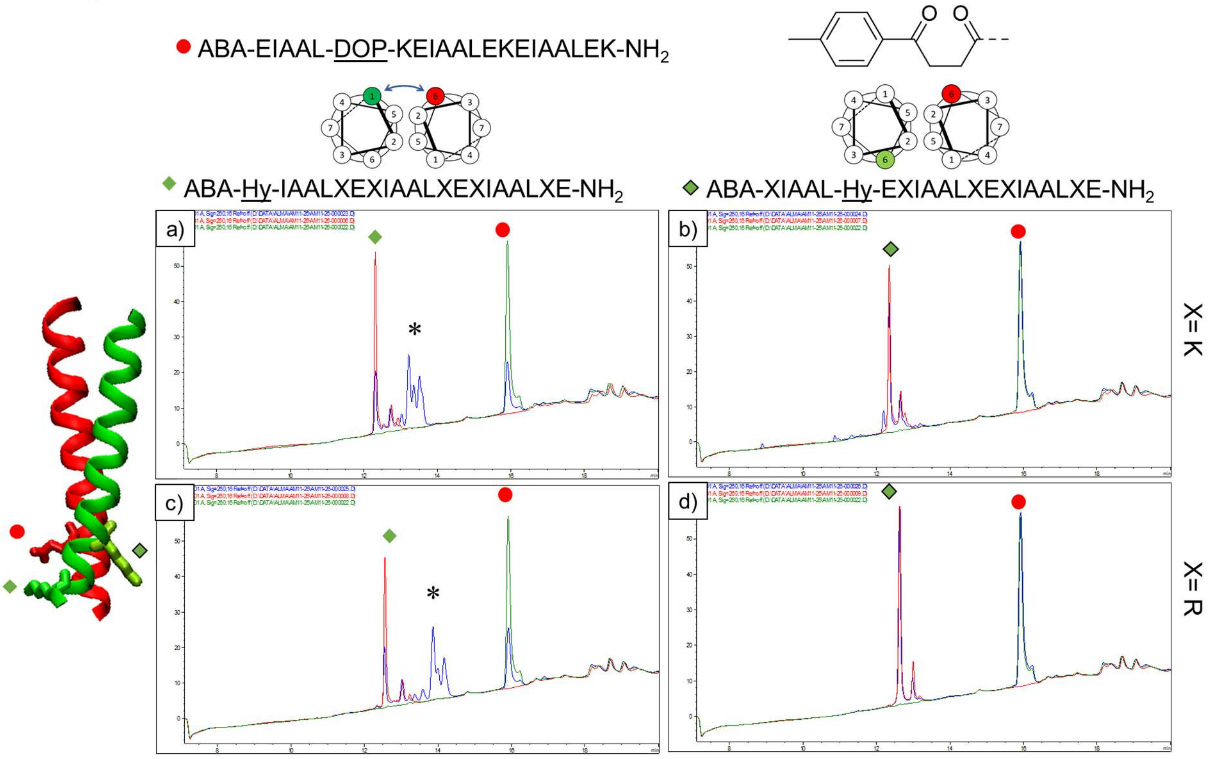

Fig. S6: HPLC1 traces of the ligation experiments performed in presence of **Coil-DOP4** (red dot): (a) with **Coil-Nu1**, (b) with **Coil-Nu1(R)**, (c) with **Coil-MM**, (d) with **Coil-MM(R)**. DOP: DOP-modified ornithine; Hy: aminoglycine-modified ornithine; For clarity, the peaks corresponding to the different hydrazine-containing probes are marked with a green diamond; *: ligation products. For chromatographic conditions, please refer to the general information section.

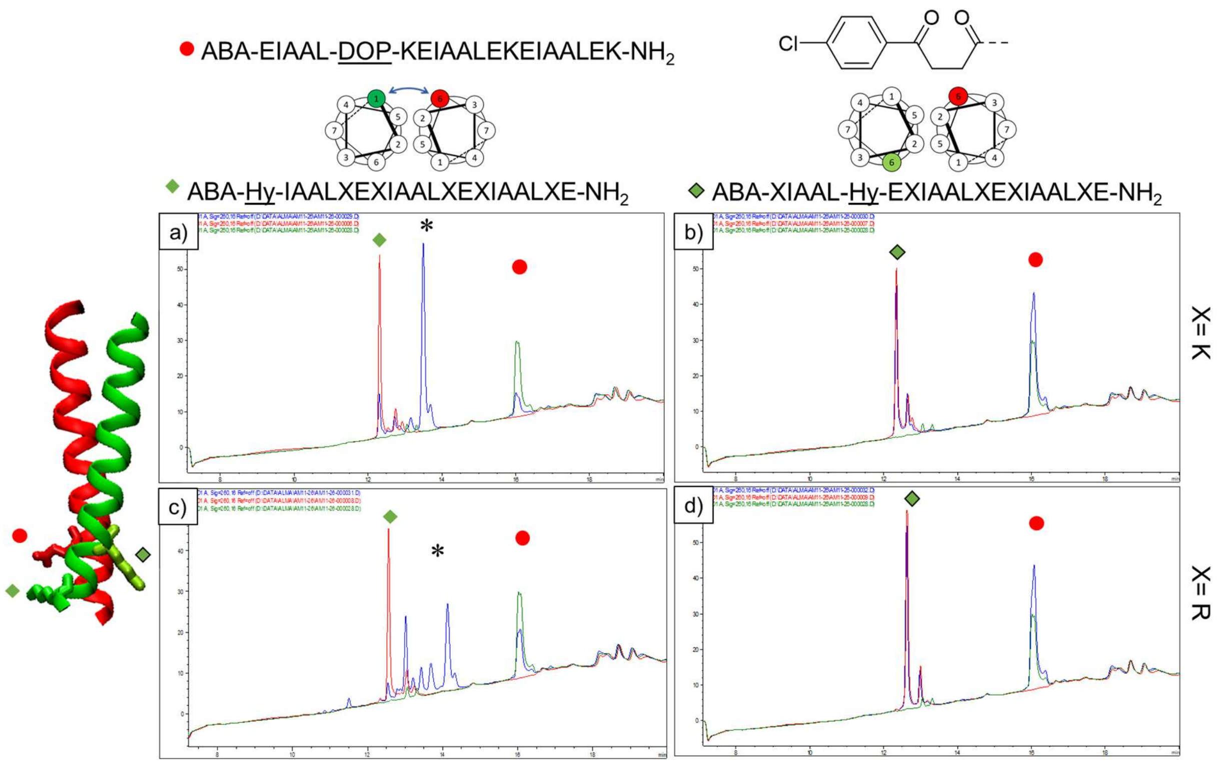

Fig. S7: HPLC1 traces of the ligation experiments performed in presence of **Coil-DOP5** (red dot): (a) with **Coil-Nu1**, (b) with **Coil-Nu1(R)**, (c) with **Coil-MM**, (d) with **Coil-MM(R)**. DOP: DOP-modified ornithine; Hy: aminoglycine-modified ornithine; For clarity, the peaks corresponding to the different hydrazine-containing probes are marked with a green diamond; *: ligation products. For chromatographic conditions, please refer to the general information section.

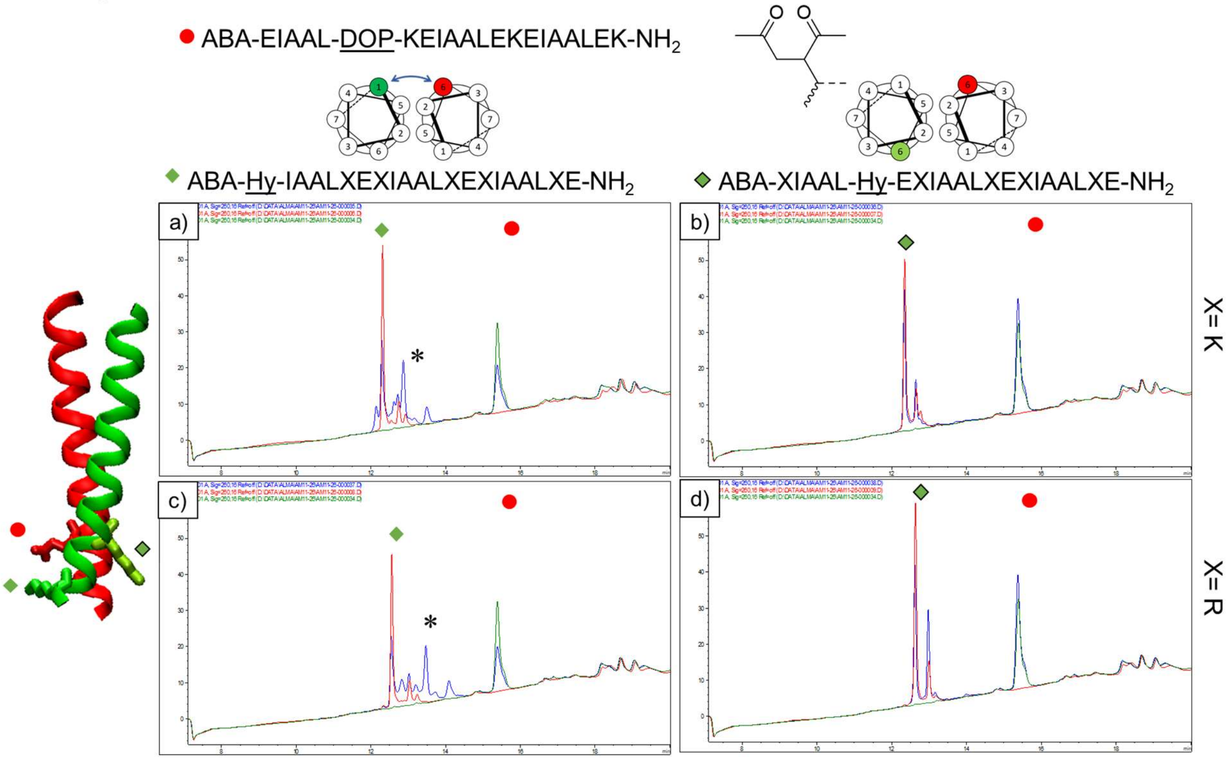

Fig. S8: HPLC1 traces of the ligation experiments performed in presence of **Coil-DOP6** (red dot): (a) with **Coil-Nu1**, (b) with **Coil-Nu1(R)**, (c) with **Coil-MM**, (d) with **Coil-MM(R)**. DOP: DOP-modified ornithine; Hy: aminoglycine-modified ornithine; For clarity, the peaks corresponding to the different hydrazine-containing probes are marked with a green diamond; *: ligation products. For chromatographic conditions, please refer to the general information section.

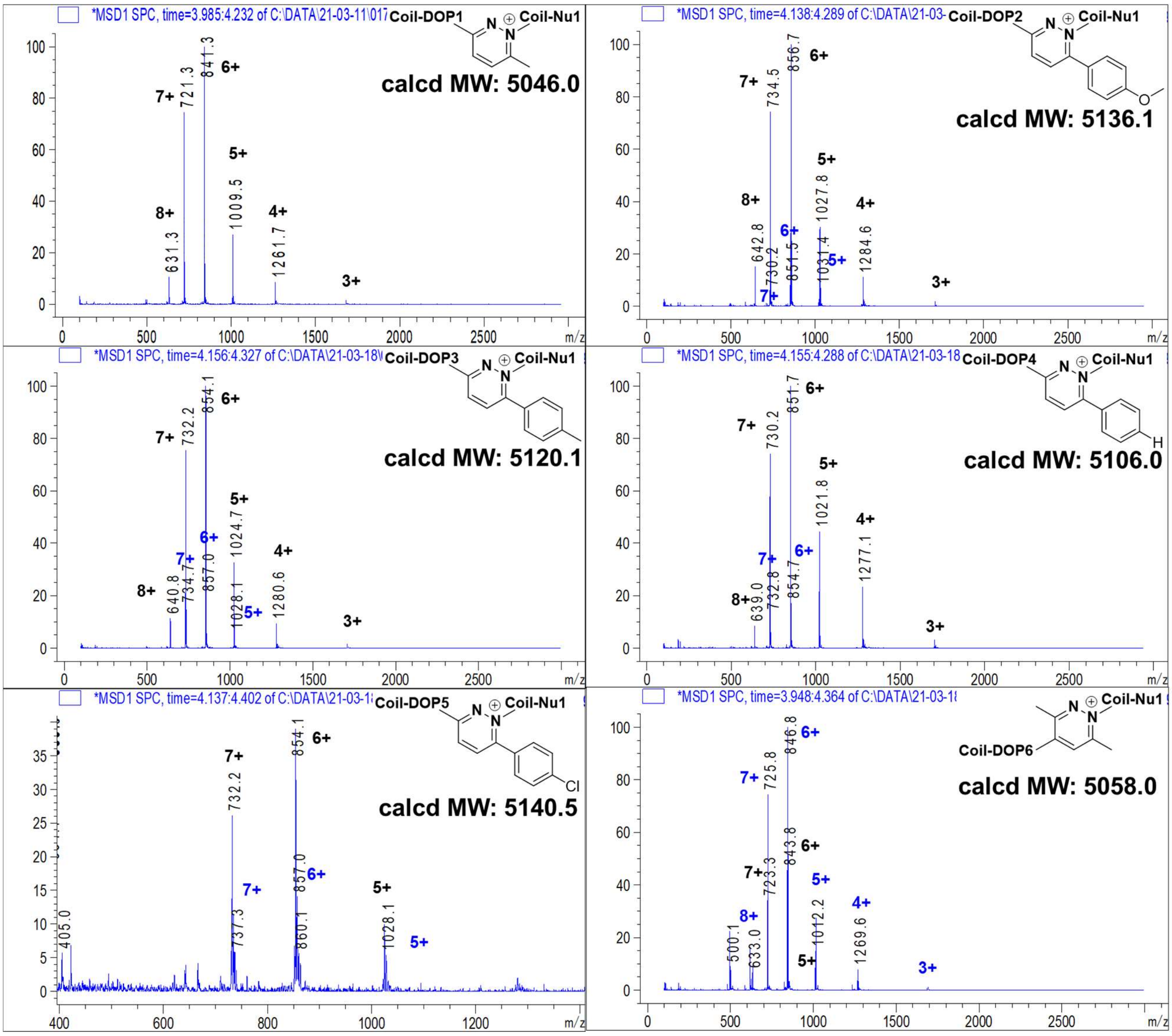

Fig. S9: ESI-MS characterization of the ligation product formed at pH 7.4 between **Coil-Nu1** and the different **Coil-DOPs.**

4. Evaluation of reaction kinetics

For short kinetic measurement times (5 h), 500 µL of PBS or BCPS buffered solution containing the first probe (5.5 µM concentration after final dilution) were prepared in a 1.5 mL Eppendorf and incubated at 25°C for 5 minutes before the addition of the second probe at 5 µM final concentration. 30 µL of the reaction mixture were collected at different time points and quenched by the addition of 6 µL of acetone. The resulting quenched solution were analyzed via HPLC-UV.

For long kinetic measurement times (24 h), 500 µL of PBS or BCPS buffered solution containing first (5.5 µM concentration after final dilution) were prepared in a HPLC vial incubated at 25°C for 5 minutes before the addition of the second probe at 5 µM final concentration. The vial was kept in a thermostated sample holder, and the reaction mixture was injected directly in HPLC-UV at different time points. Due to technical reasons, the injection of samples at 0h was delayed of 2 minutes (therefore t_0 is 0.03h).

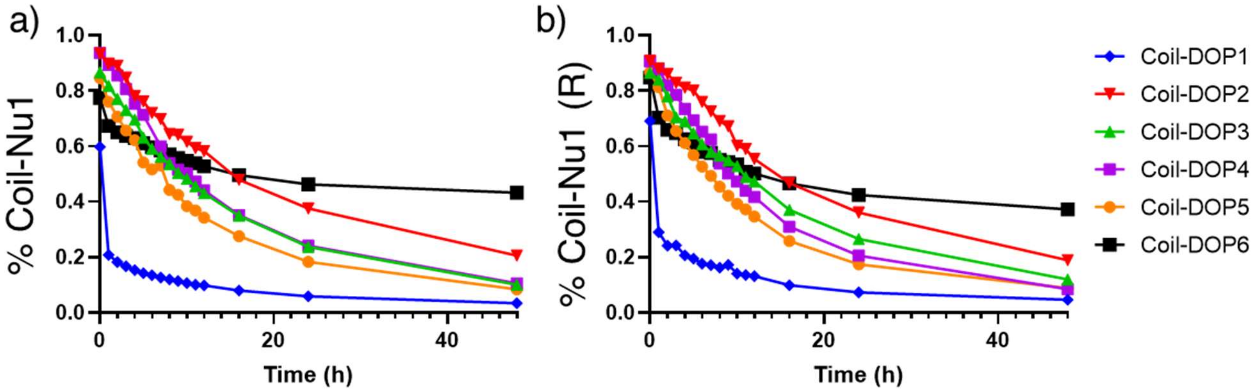

Fig. S10: Consumption profile of the nucleophile-containing coil **Coil-Nu1** (a) or **Coil-Nu1(R)** (b) in presence of 1.1 eq of different DOP-containing coils. All experiments were conducted at 5 µM coil concentration, pH 7.4, 25°C.

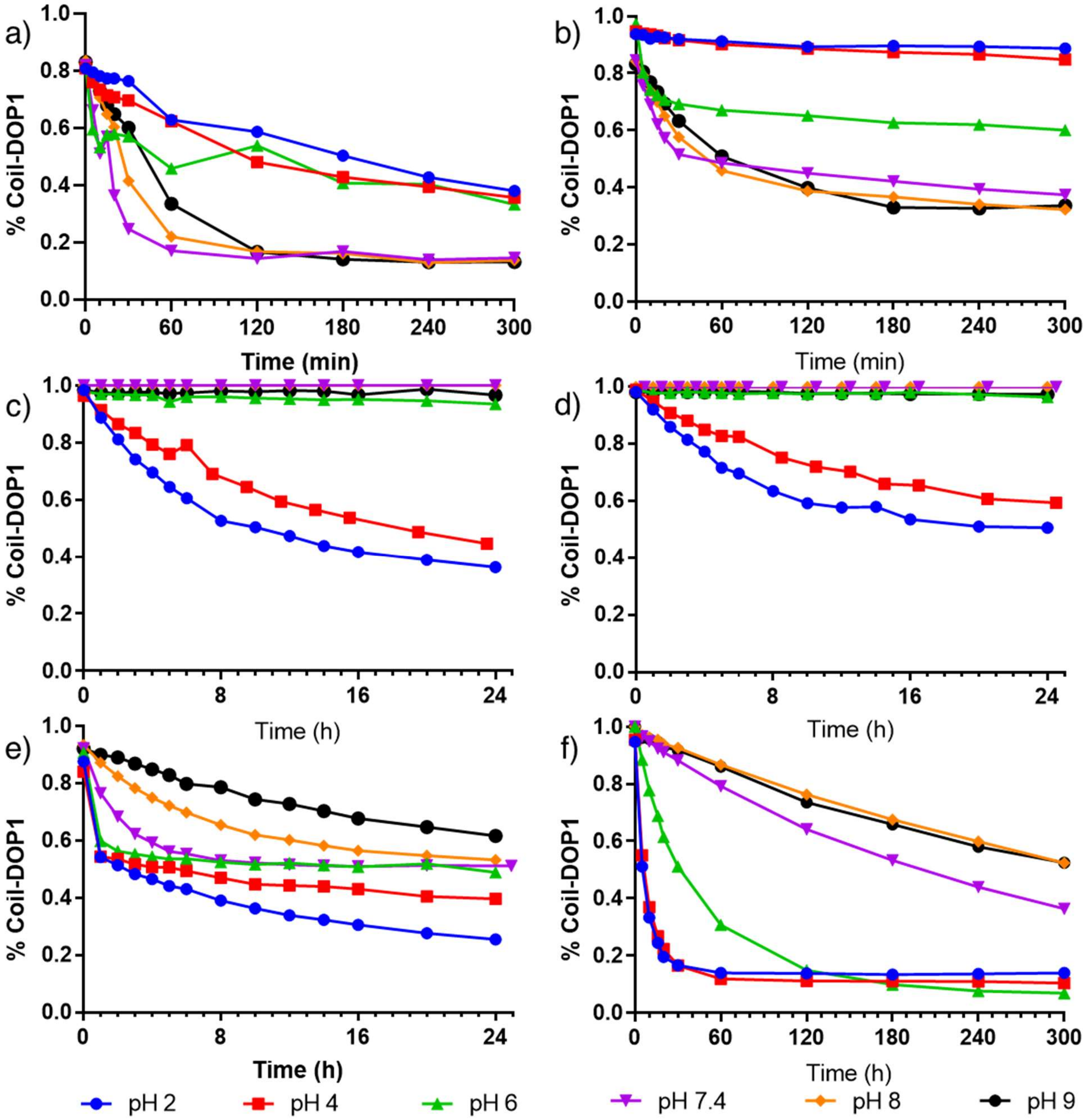

Fig. S11: Consumption profile of the nucleophile-containing coil in presence of 1.1 eq of **Coil-DOP1**. (a) **Coil-Nu1** in open vial; (b) **Coil-Nu1** in closed vial; (c) **Coil-Nu2**; (d) **Coil-Nu3**; (e) **Coil-Nu4**; (f) **Coil-Nu5**. All experiments were conducted at 5 μM coil concentration at 25°C.

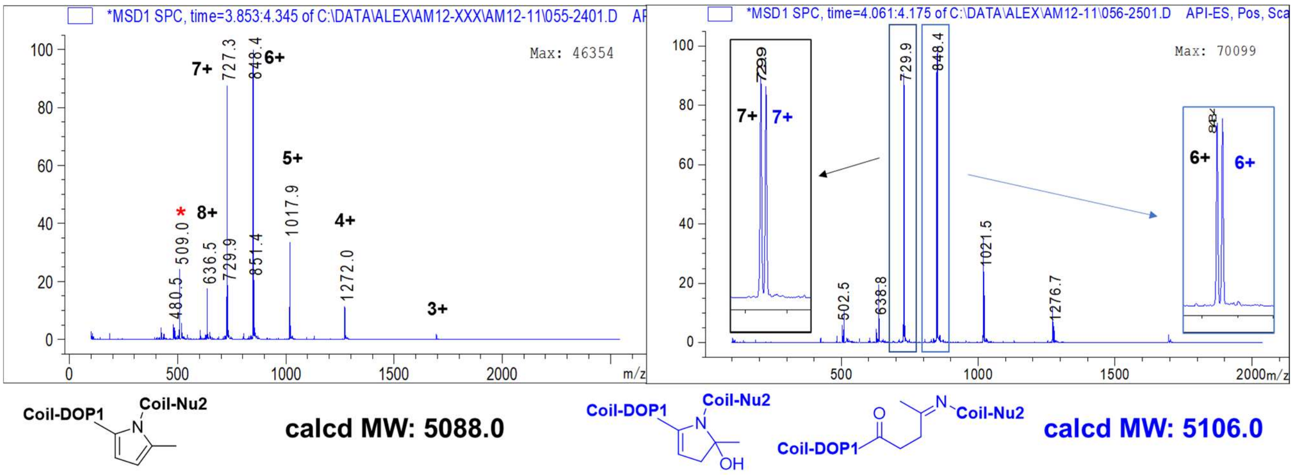

Fig. S12: ESI-MS characterization of the ligation product formed between Coil-DOP1 and Coil-Nu2 under acid (left) and neutral (right) conditions; (*) indicates starting material signals.

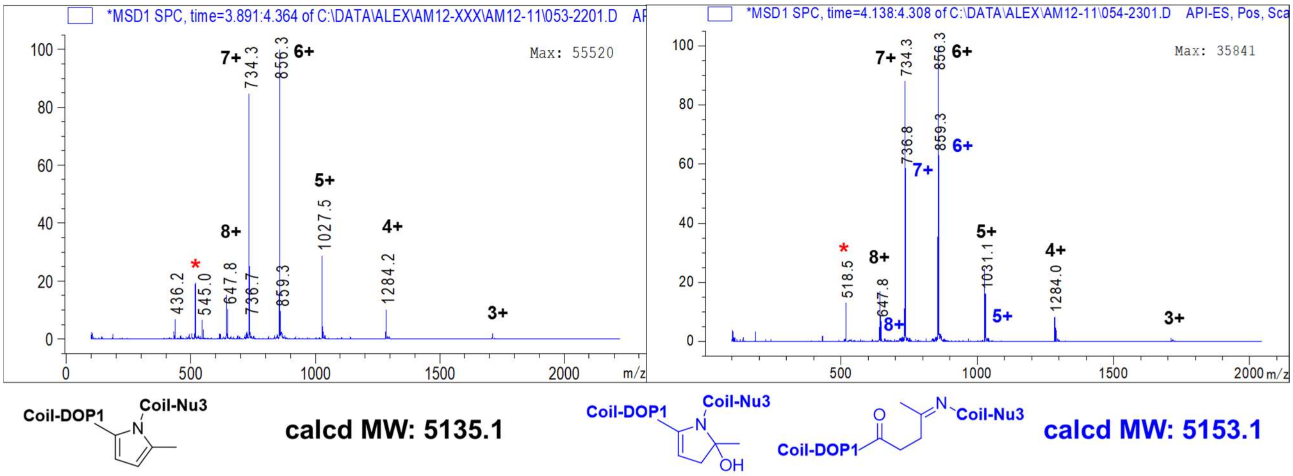

Fig. S13: ESI-MS characterization of the ligation product formed between **Coil-DOP1** and **Coil-Nu3** under acid (left) and neutral (right) conditions; (*) indicates starting material signals.

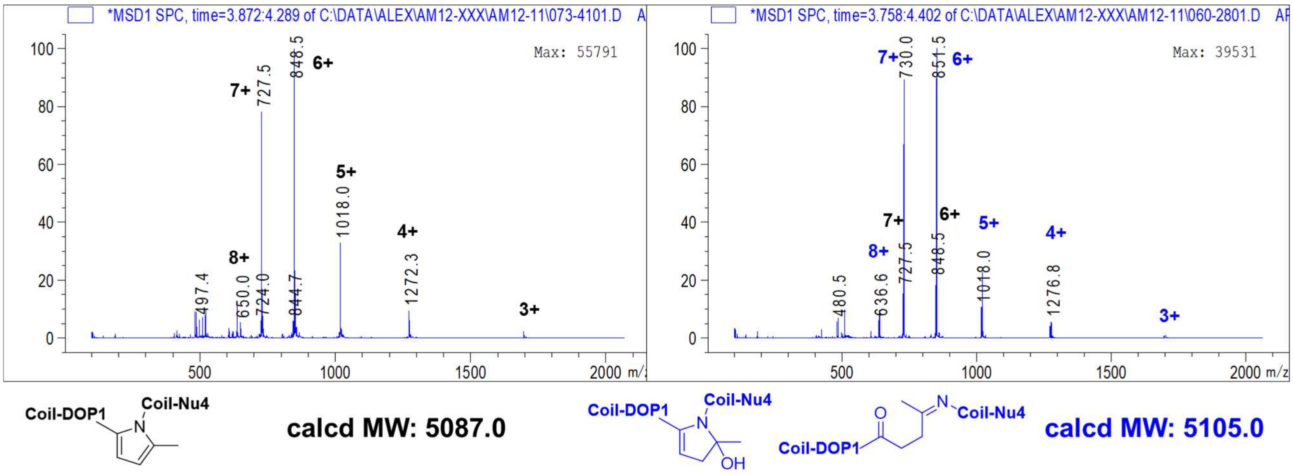

Fig. S14: ESI-MS characterization of the ligation product formed between **Coil-DOP1** and **Coil-Nu4** under acid (left) and neutral (right) conditions; (*) indicates starting material signals.

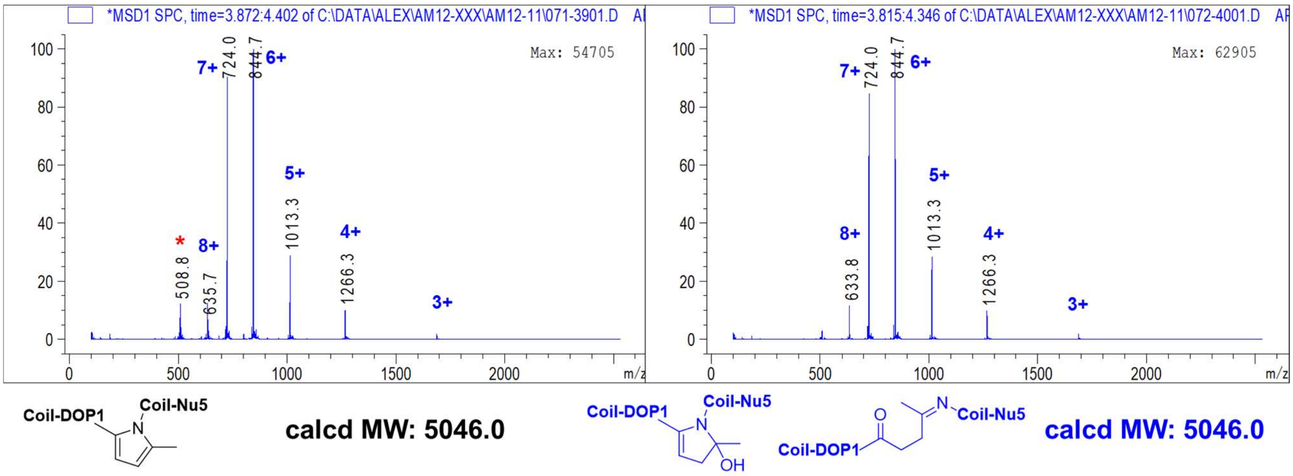

Fig. S15: ESI-MS characterization of the ligation product formed between **Coil-DOP1** and **Coil-Nu5** under acid (left) and neutral (right) conditions; (*) indicates starting material signals.

5. Structural investigation on ligation with aminooxy moieties

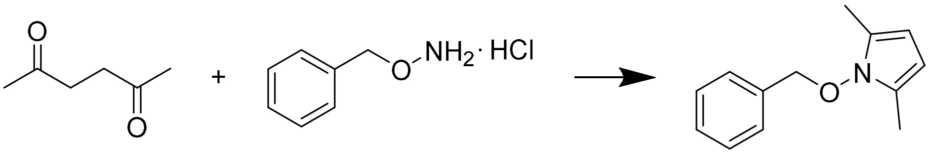

To a solution of 100 mg hexan-2,5-dione (876 µmol) in 1 mL DMF, a dispersion of 140 mg of O-benzylhydroxyamine hydrochloride (876 µmol, 1 eq) in 1 mL DMF was added dropwise. The reaction was monitored over 2h with analytical HPLC5 and the product was purified via Preparative RP-LC flash chromatography (Gilson® PLC 2250 equipped with PrepPak® cartridge, Waters 1000, Delta-pak C18 100Å, 0-100% gradient of MeCN in water containing 0.1% TFA, using a flow rate of 65 mL/min. Collected fractions were freeze-dried and the resulting amorphous white solid was analyzed via ^{1}H-NMR and re-injected in HPLC to exclude influences of the drying process to the structure of the molecule.

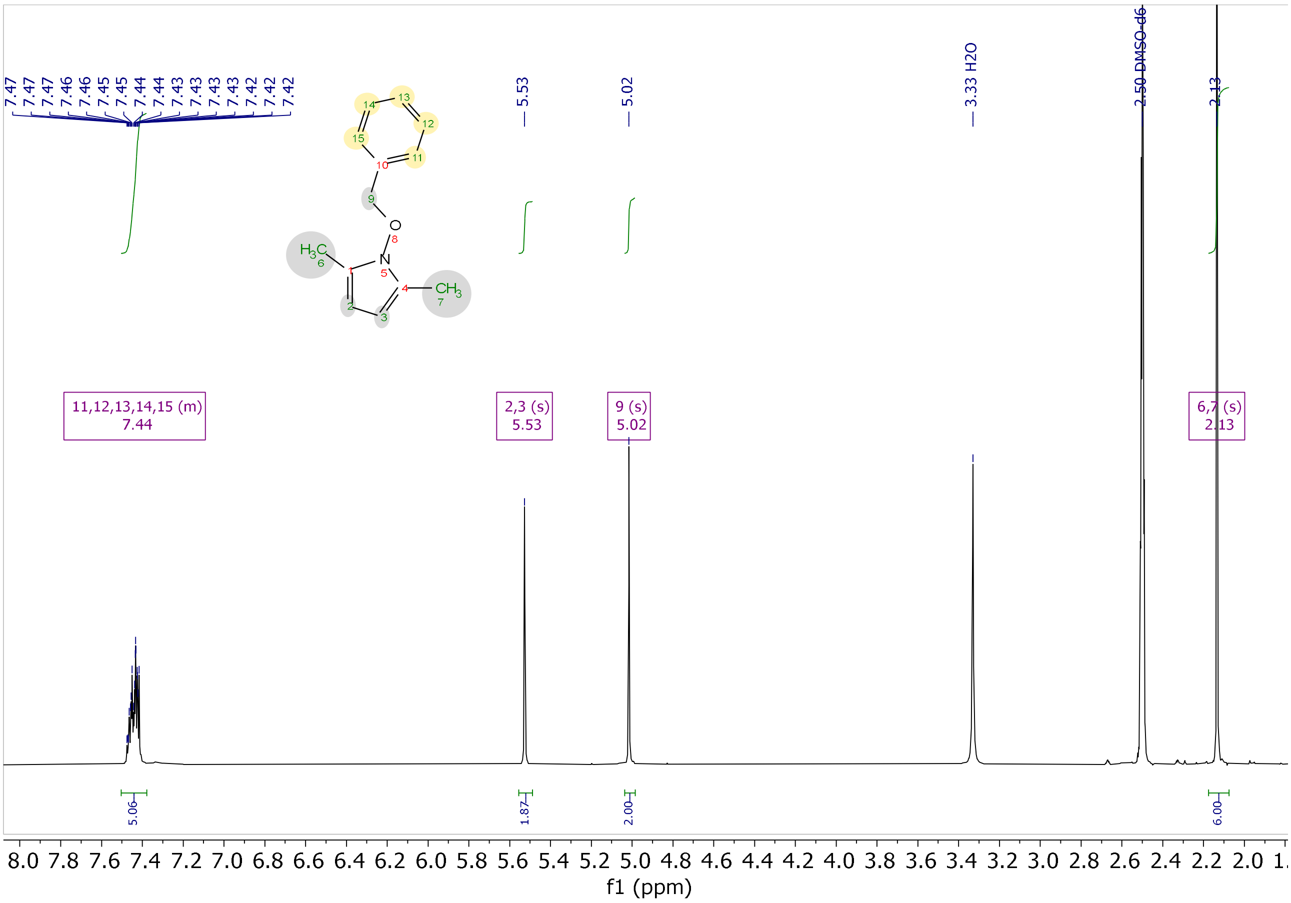

Fig. S16: ^{1}H-NMR (400 MHz, DMSO-d^{6}) of isolated compound.

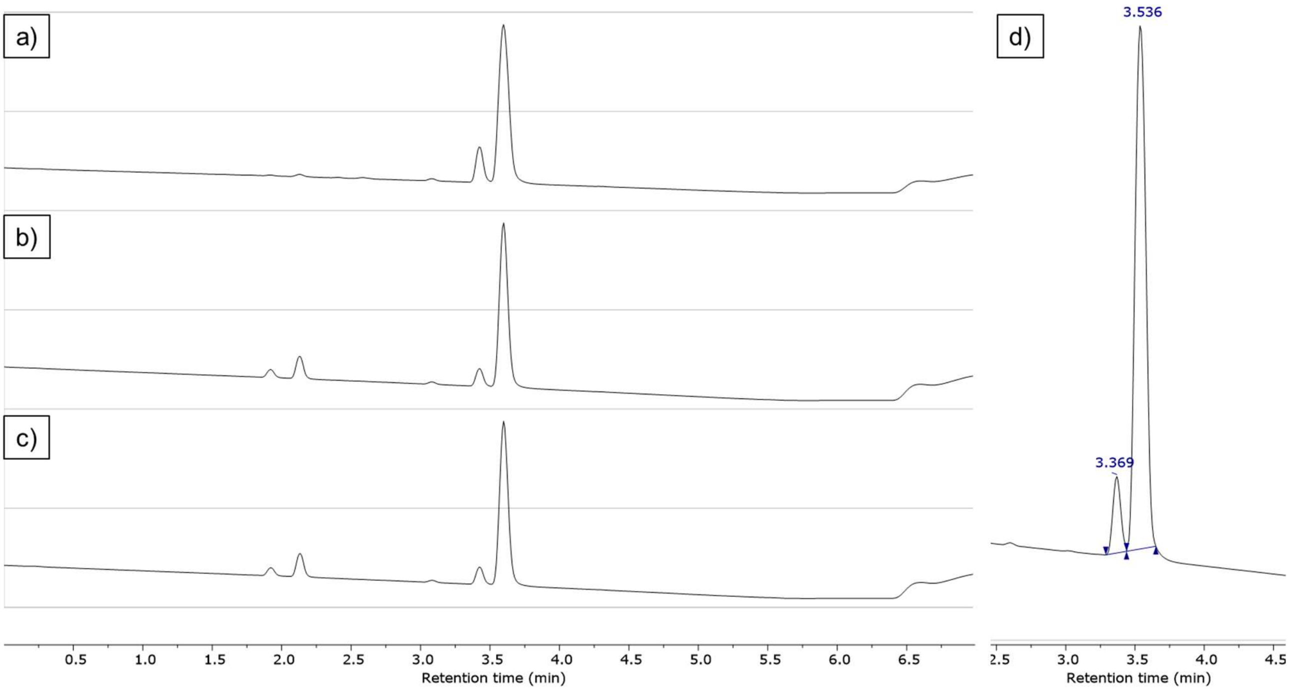

Fig. S17: HPLC5 traces of the reaction mixture after 5 minutes (a), 1 h (b), 2 h (c) and of the purified compound (d). For chromatographic conditions, please refer to the general information section.

6. Cyclization – Coiled peptide

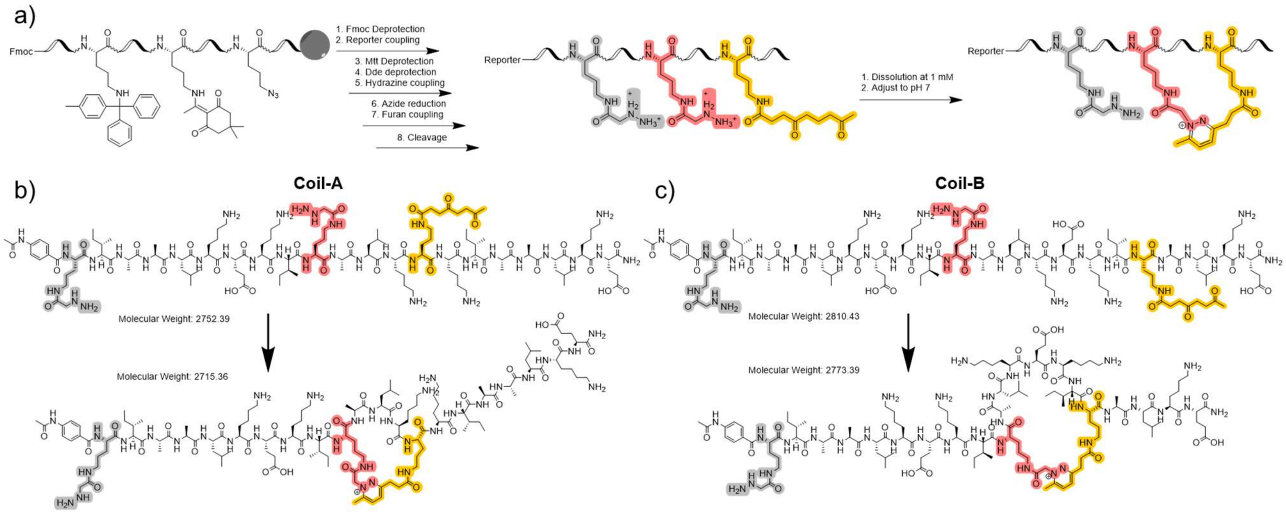

Fig. S18: a) Scheme of peptide functionalization and formation of cyclized product; b) Structure of linear and cyclized **Coil-A**; c) Structure of linear and cyclized **Coil-B**.

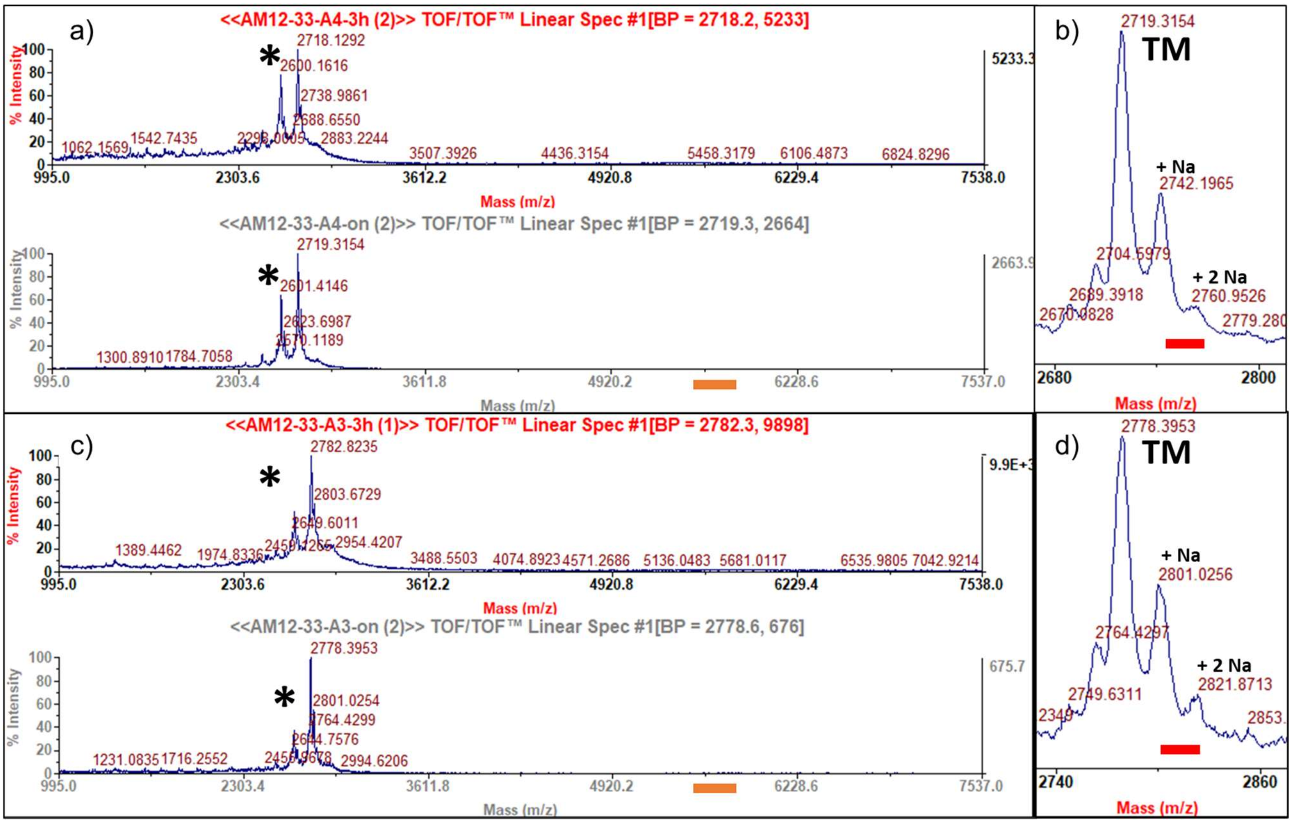

Fig. S19: MALDI analysis of the crude peptides after 3h and overnight reaction (a, c) and focus on the region of the target molecule (b, d). a,b) **Coil-A**, c,d) **Coil-B**. * indicate deletion products missing a Ile/Leu residue; orange bars indicate the m/z region of dimeric compounds; red bars indicate the m/z region of the starting material.

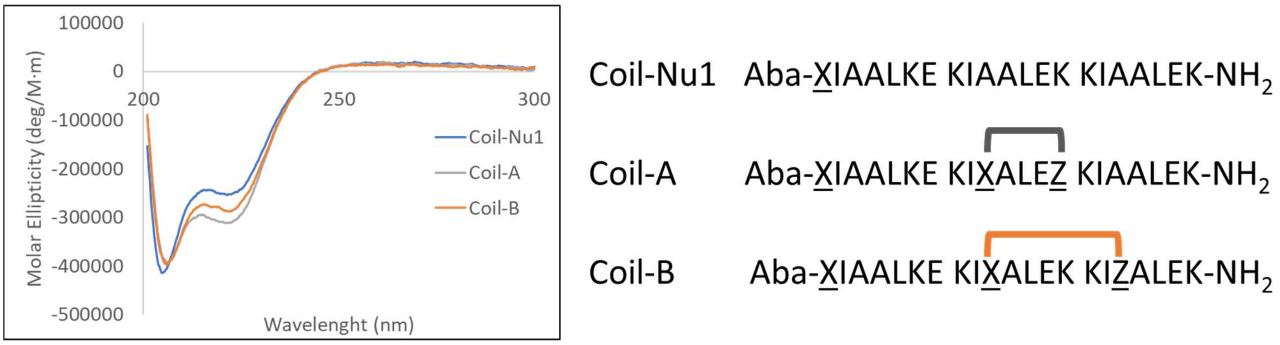

Fig. S20: CD spectra of cyclized **Coil-A** and **Coil-B** and reference peptide **Coil-Nu1**. Experiment performed at 25°C in PBS pH 7.4 at 5 µM probe concentration. X: hydrazine-bearing ornithine, Z: DOP-bearing ornithine.

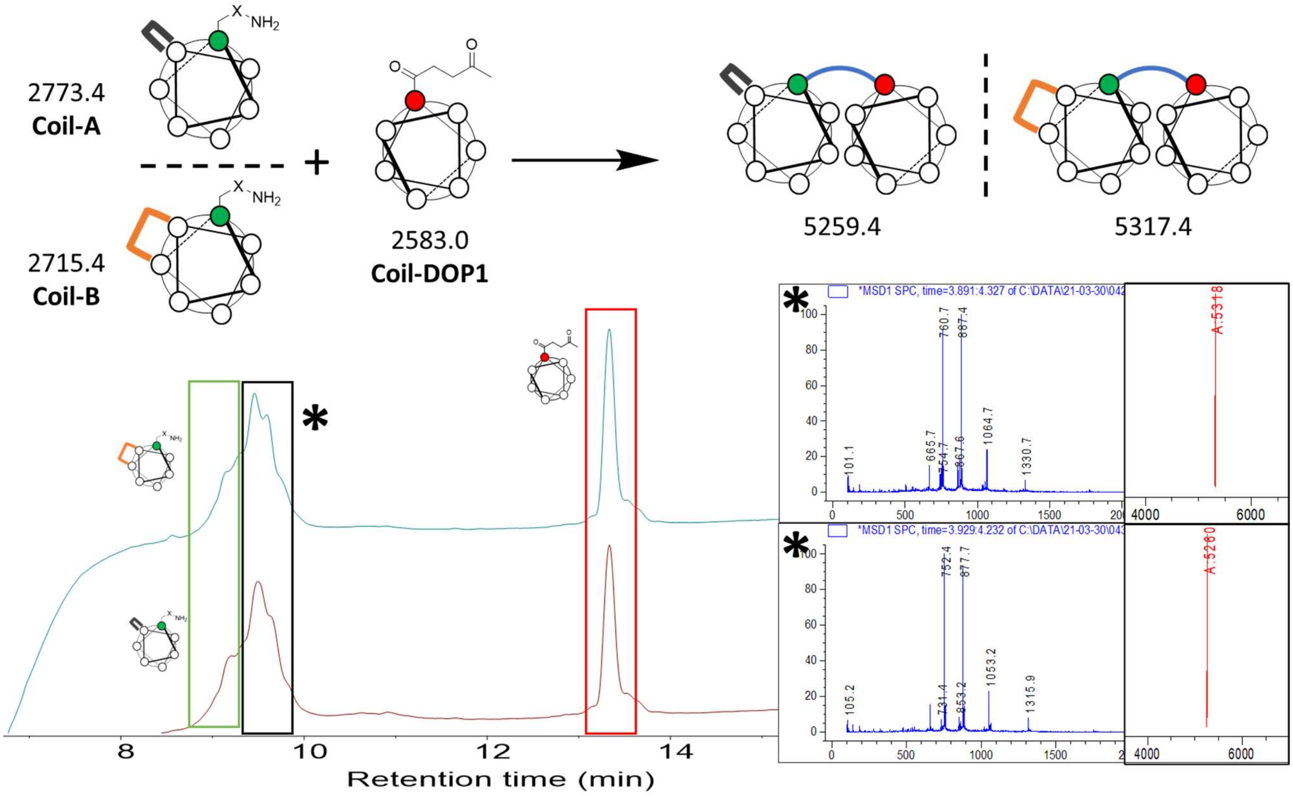

Fig. S21: Ligation experiments performed between **Coil-DOP1** and cyclized **Coil-A** or **Coil-B**. Experiments were performed at 5 µM probes concentration in PBS 7.4. For chromatographic conditions, please refer to the general information section.

7. Cyclization – Bovine RHAU (G4 binder)

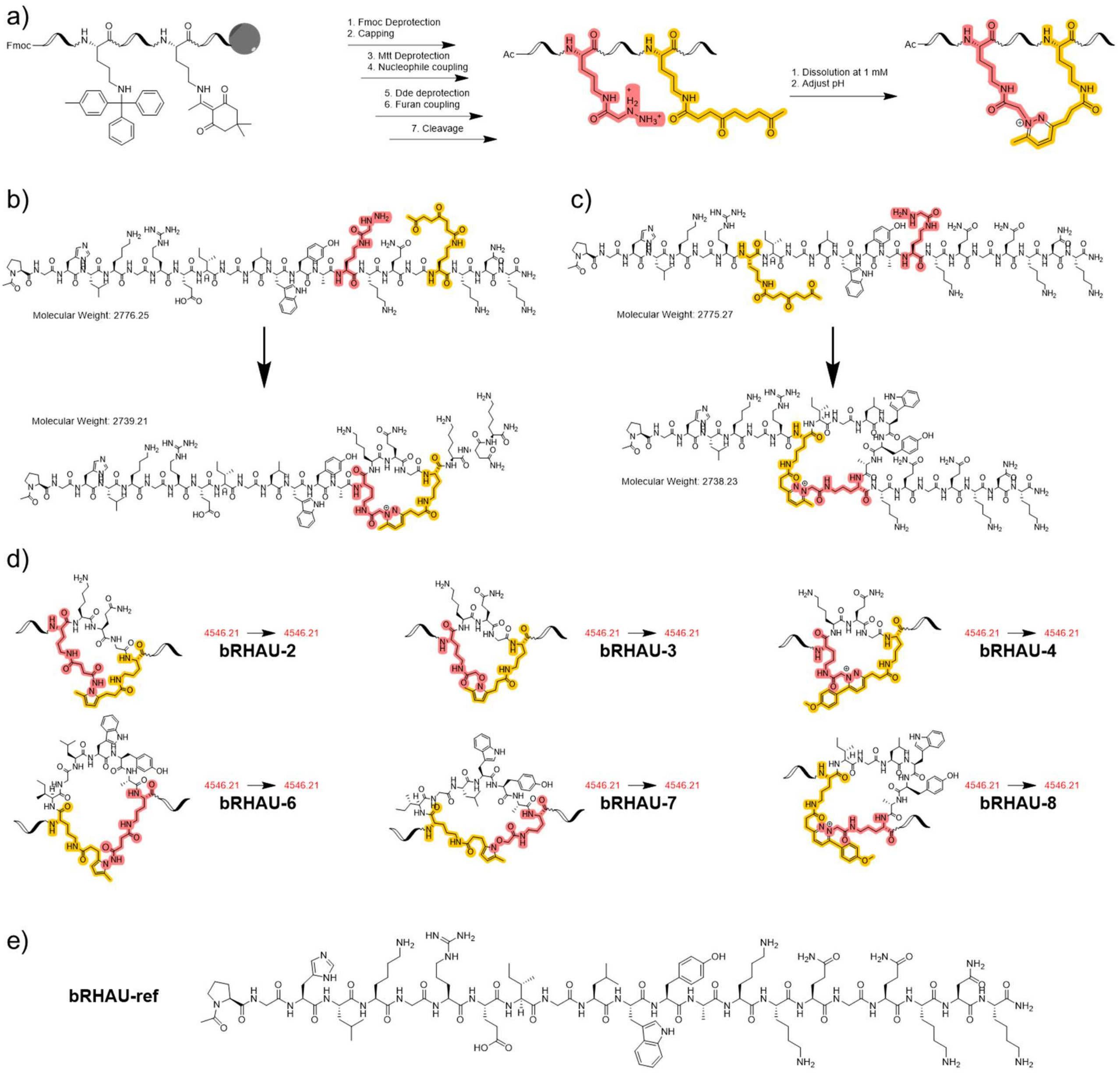

Fig. S22: a) general scheme of peptide functionalization and formation of cyclized products; b) structure of linear and cyclized **bRHAU-1**; c) structure of linear and cyclized **bRHAU-5**; d) structure of the macrocycles present in **bRHAU-2**, **bRHAU-3**, **bRHAU-4**, **bRHAU-6**, **bRHAU-7**, and **bRHAU-8**; e) structure of **bRHAU-ref**.

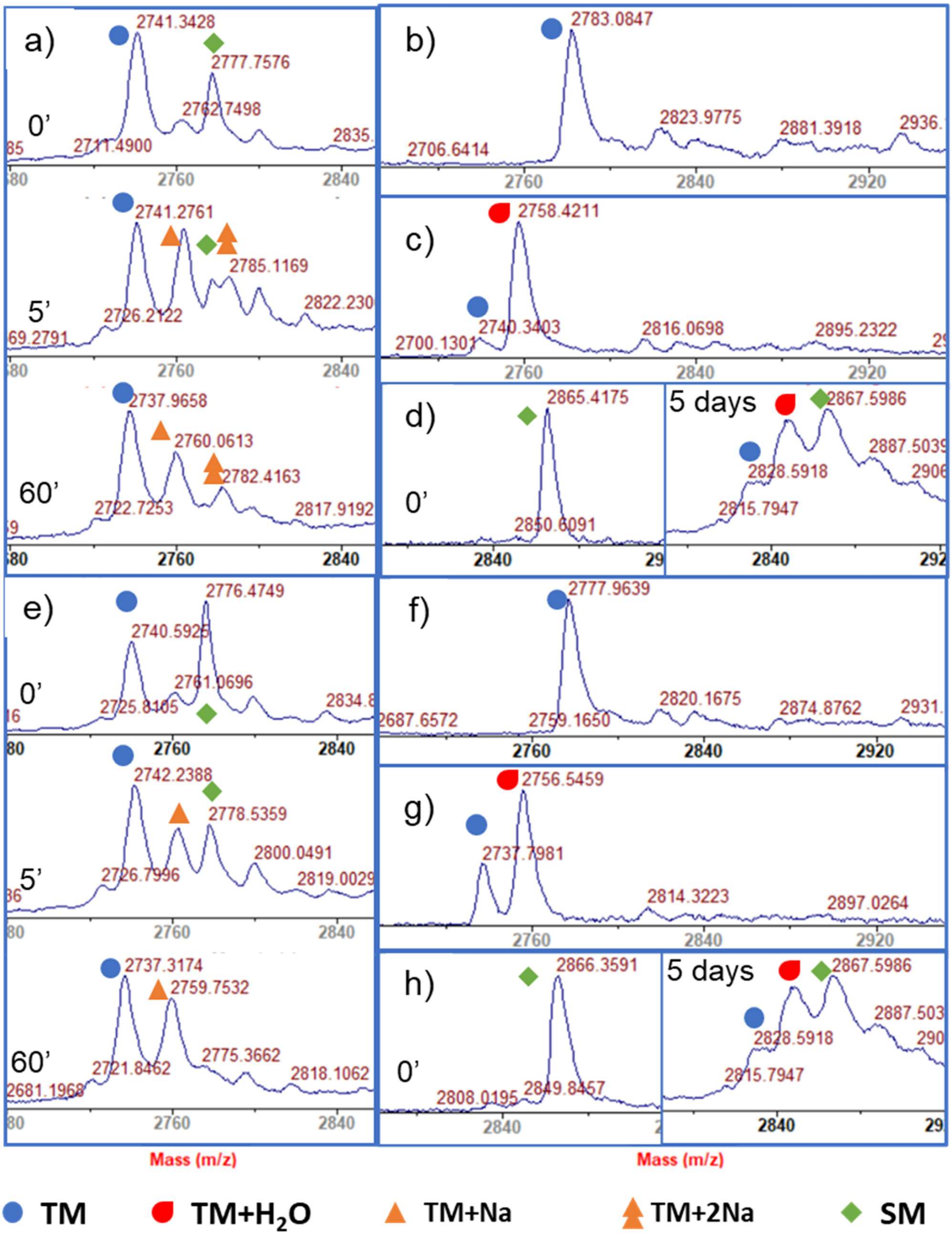

Fig. S23: MALDI analysis of the crude peptides after dissolution in mQ water at 5 mM concentration (0’) and after specified time in pH adjusted solutions. a) **bRHAU-1**, pH 7.5; b) **bRHAU-2**, pH 2.5; c) **bRHAU-3**, pH 2.5; d) **bRHAU-4**, pH 7.5; e) **bRHAU-5**, pH 7.5; f) **bRHAU-6**, pH 2.5; g) **bRHAU-7**, pH 2.5; g) **bRHAU-8**, pH 7.5.

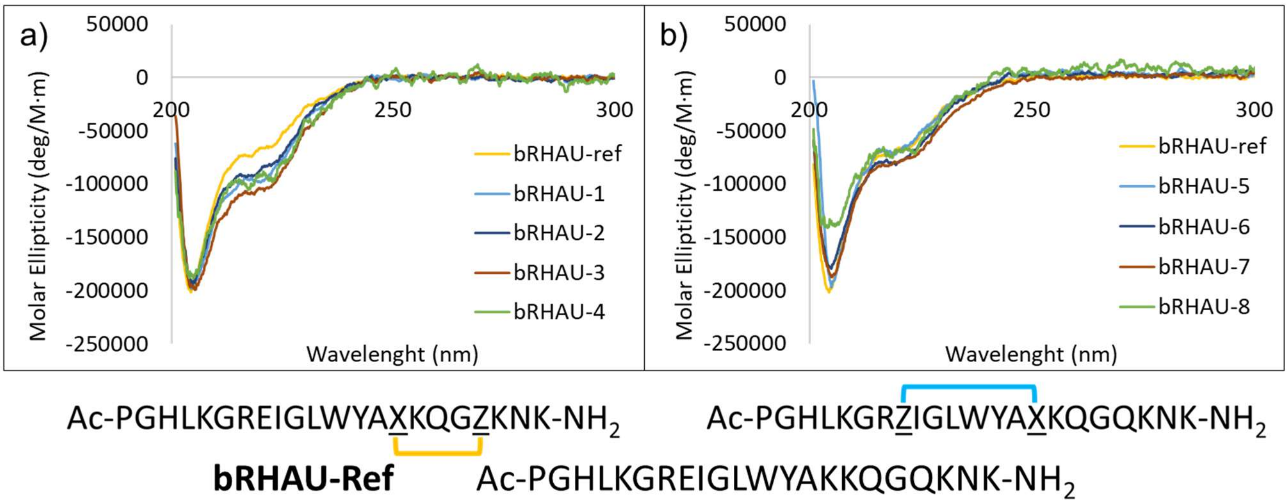

Fig. S24: CD spectra of cyclized bRHAU peptides compared to **bRHAU-ref**. a) **bRHAU-1**, **bRHAU-2**, **bRHAU-3**, and **bRHAU-4; b) bRHAU-5**, **bRHAU-6**, **bRHAU-7**, and **bRHAU-8**. General structure of the peptides is reported below each graph. Experiments were performed at 25°C in PBS pH 7.4 at 5 µM probe concentration. X: hydrazine-bearing ornithine, Z: DOP-bearing ornithine.

7.1 Peptide macrocycle chemical stability

100 µL of buffered solution (PBS pH 7.4 or BCPS pH 2.0) containing peptides at 50 µM concentration (from a 200 µM stock solution), were prepared in a 0.2 mL Eppendorf in the presence of 20 % acetone. Mixtures were allowed to react up to 24h at 37°C. At different time points, solutions were sampled and analyzed via MALDI-TOF to evaluate the eventual opening of the macrocycle and the reaction of the α-nucleophile with acetone.

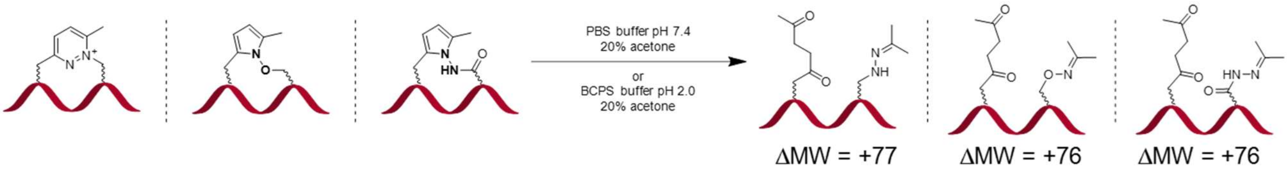

	+ H			+ Na			+ K		
	Linear	Macrocycle	+Acetone	Linear	Macrocycle	+Acetone	Linear	Macrocycle	+Acetone
**bRHAU-ref**	2578.03	-	2618.10	2600.02	-	2640.09	2616.13	-	2656.20
**bRHAU-5**	2775.27	2738.24	2815.34	2797.26	2760.23	2837.33	2813.37	2776.34	2853.44
**bRHAU-6**	2817.30	2781.27	2857.36	2839.29	2803.26	2879.35	2855.40	2819.37	2895.46
**bRHAU-7**	2776.25	2740.22	2816.31	2798.24	2762.21	2838.30	2814.35	2778.32	2854.41

Fig. S25: general scheme of reaction of cyclized peptides in presence of acetone and relative molecular weights of proton, sodium, and potassium adducts.

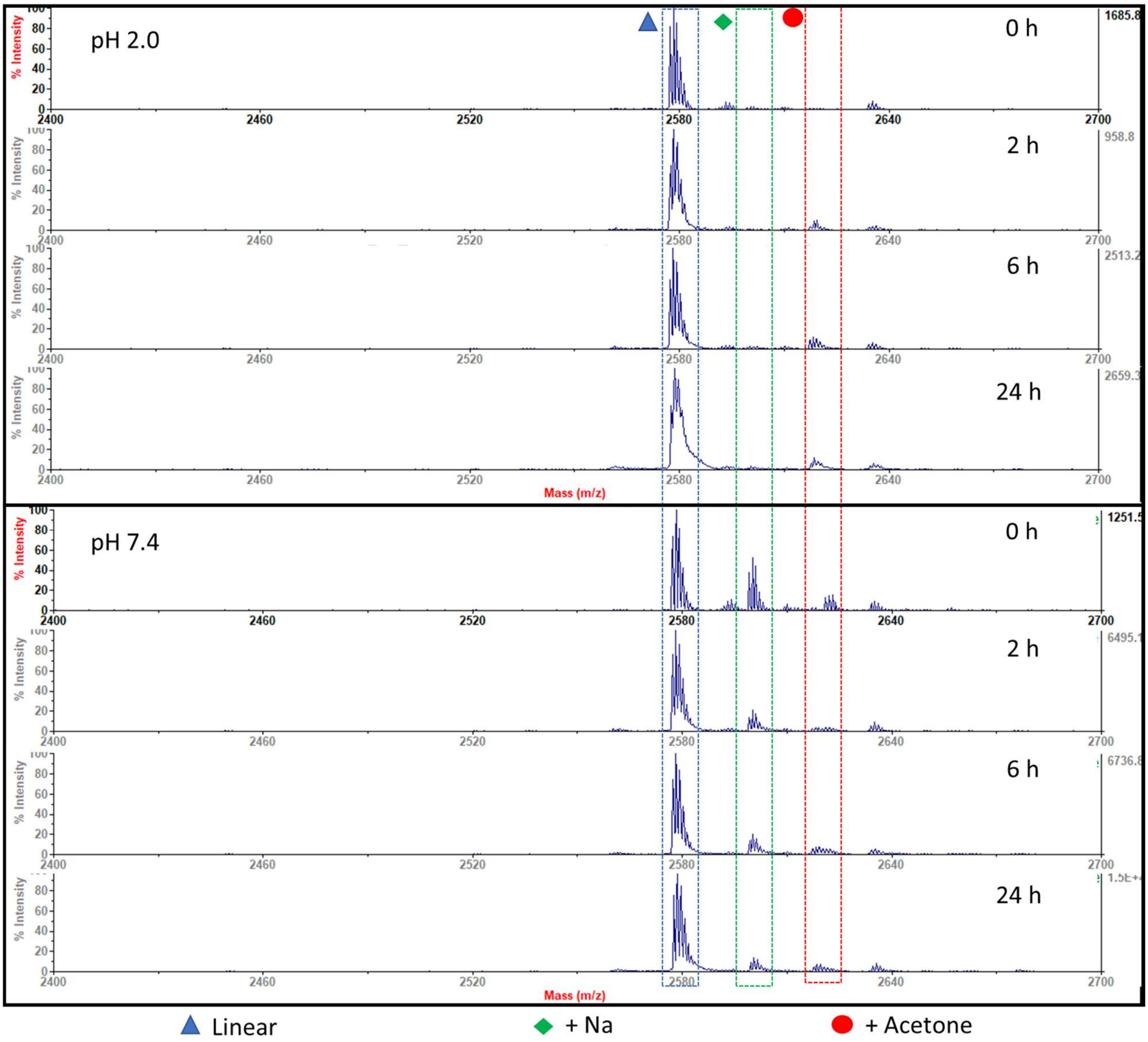

Fig. S26: MALDI analysis (reflectron mode) of the **bRHAU-ref** after solubilization in a buffered solution (pH 2.0 top, pH 7.4 bottom) containing 20% acetone. Experiments were performed at 50 µM peptide concentration.

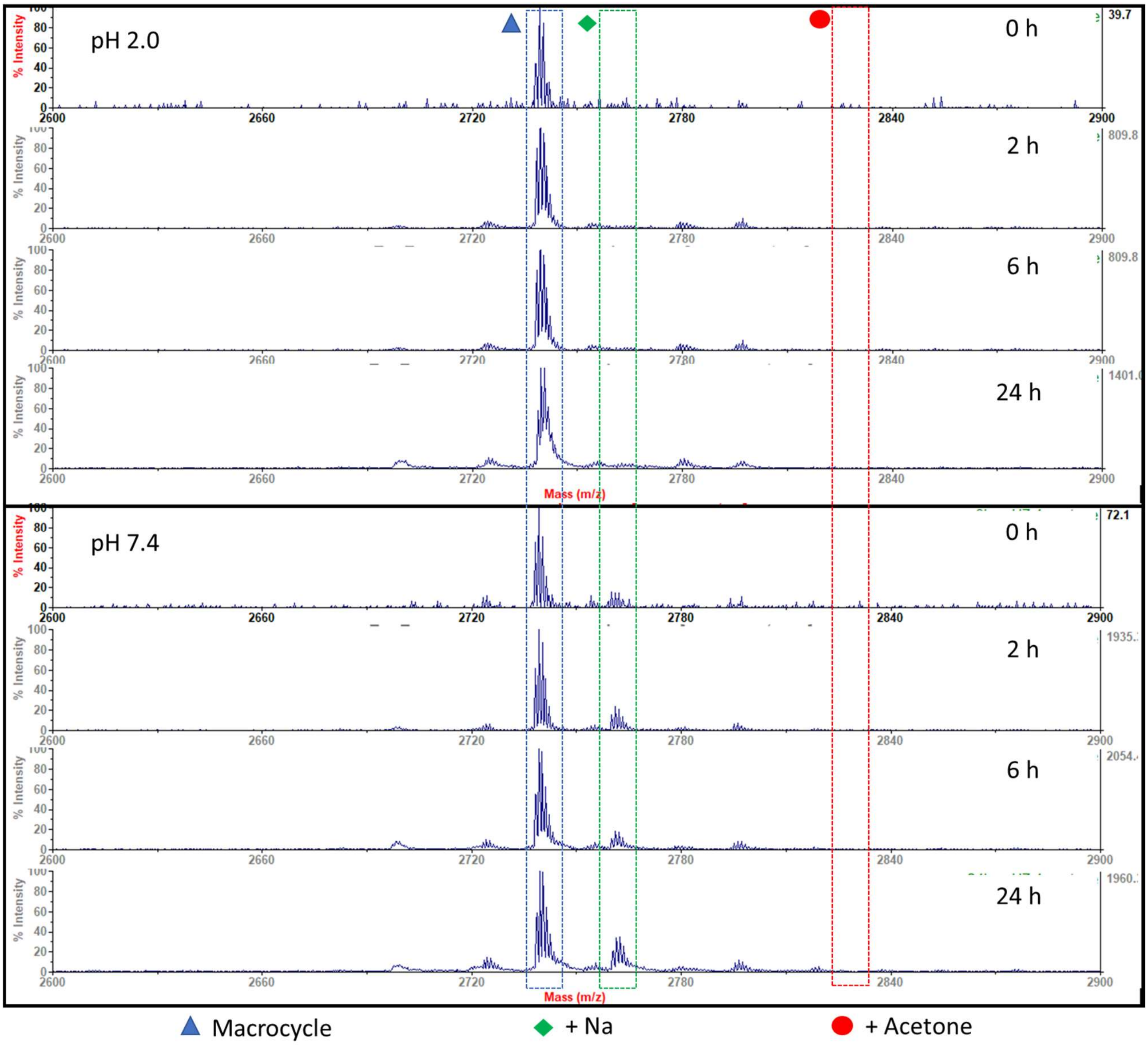

Fig. S27: MALDI analysis (reflectron mode) of the **bRHAU-5** after solubilization in a buffered solution (pH 2.0 top, pH 7.4 bottom) containing 20% acetone. Experiments were performed at 50 µM peptide concentration.

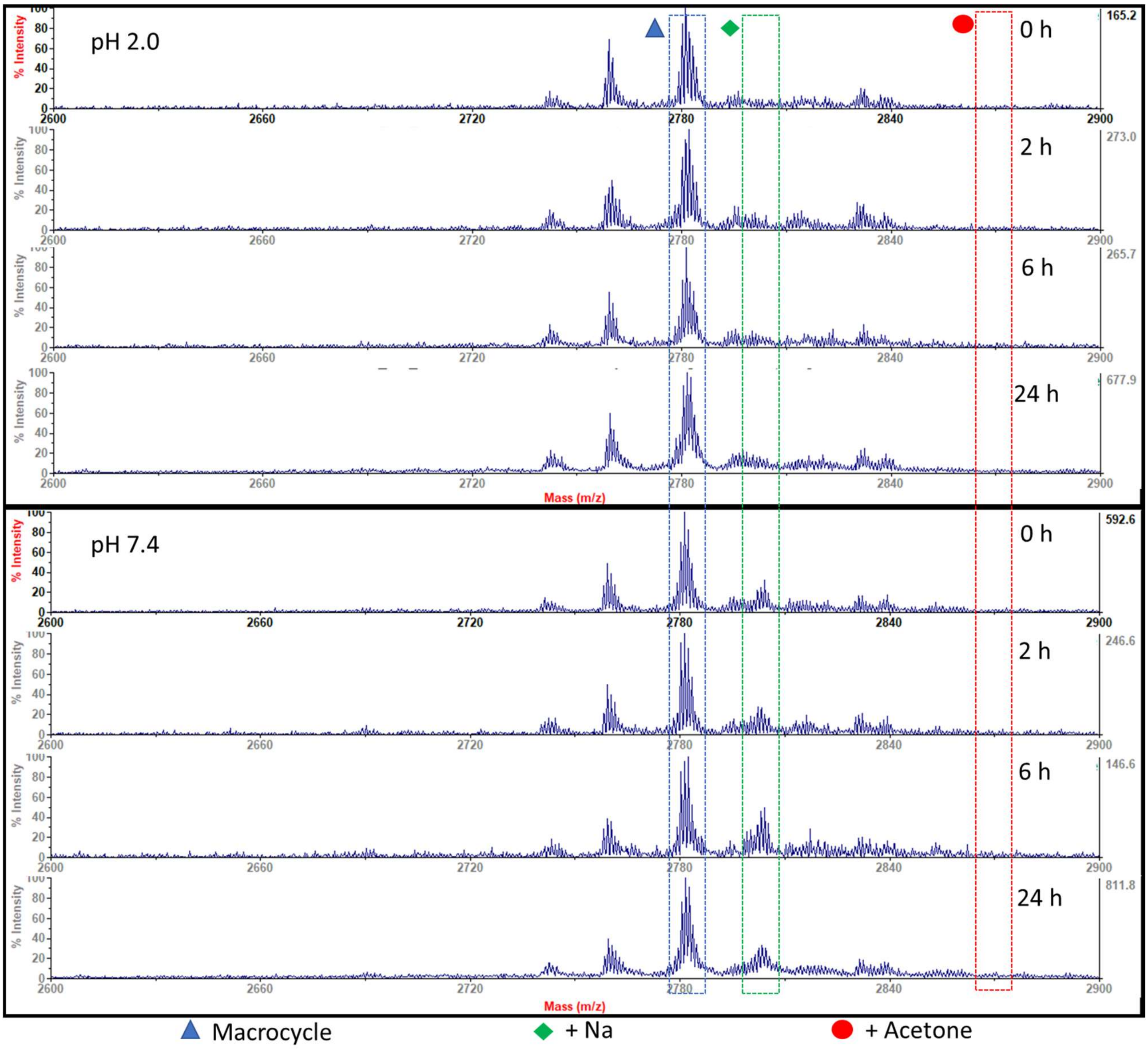

Fig. S28: MALDI analysis (reflectron mode) of the **bRHAU-6** after solubilization in a buffered solution (pH 2.0 top, pH 7.4 bottom) containing 20% acetone. Experiments were performed at 50 µM peptide concentration.

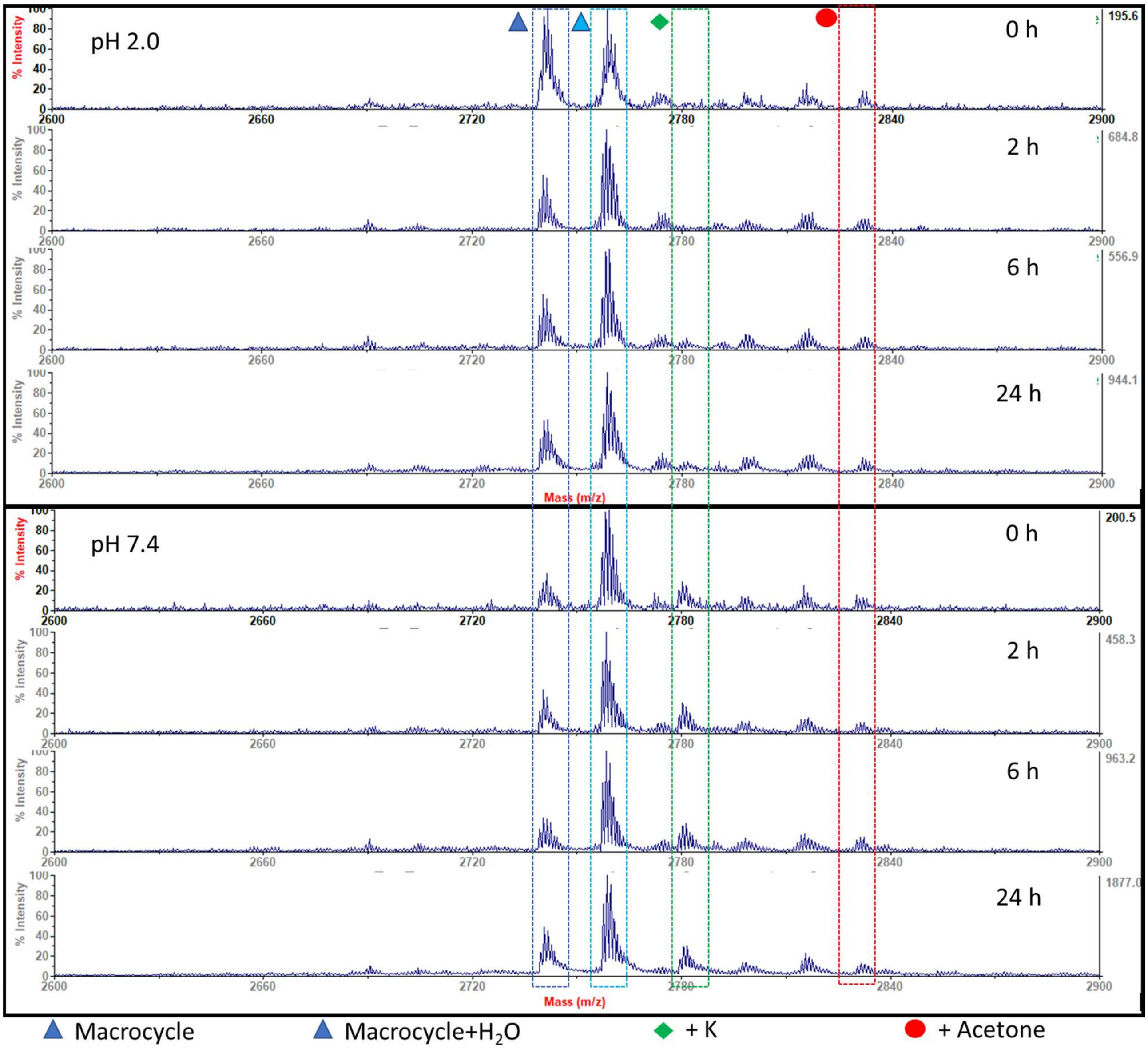

Fig. S29: MALDI analysis (reflectron mode) of the **bRHAU-7** after solubilization in a buffered solution (pH 2.0 top, pH 7.4 bottom) containing 20% acetone. Experiments were performed at 50 µM peptide concentration.

7.2 Trypsin stability

200 µL of buffered solution (PBS pH 7.4) containing peptides at 50 µM final concentration (from a 200 µM stock solution), were prepared in a 1.5 mL Eppendorf and allowed to equilibrate to 37°C. In a separate Eppendorf tube, Trypsin was equilibrated to 37°C at 10X concentration. After 5 minutes of equilibration, Trypsin was added to the peptides' solutions to 0.1 µg/mL final concentration. At different time points, 20 µL of sample were mixed with 20 µL 1% TFA in mQ to inactivate the enzyme and analyzed via HPLC-UV.

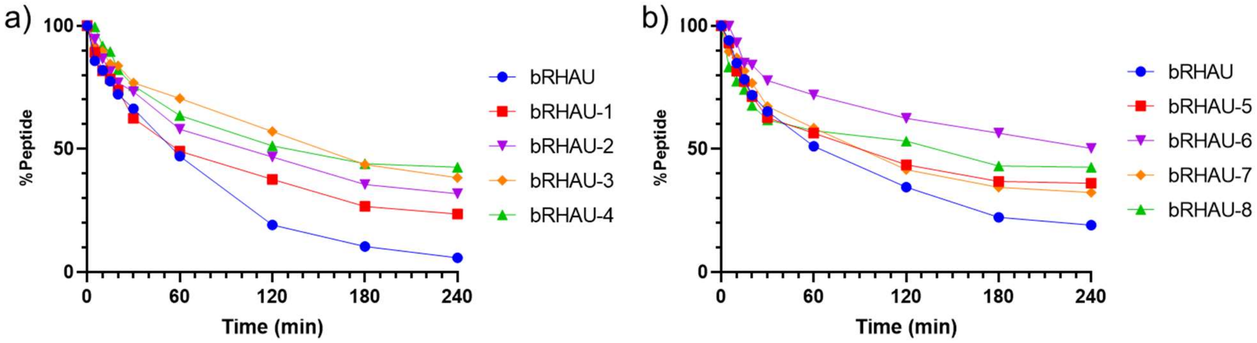

Fig. S30: degradation profile of the peptides in presence of trypsin. (a) relative degradation of (*i,i+4*) peptides compared to linear bRHAU; (b) relative degradation of (*i,i+7*) peptides compared to linear **bRHAU**. All experiments were conducted at 50 µM peptide concentration at 37°C.

7.3 Thermal denaturation experiments

Thermal denaturation profiles were measured by monitoring the absorbance at 295 nm from 15°C to 90°C and from 90°C to 15°C with a heating/cooling rate of 1°C/min and recording every 0.1°C. cMYC DNA (5'-TGA GGG TGG GTA GGG TGG GTA-3') 20 µM in TRIS buffer (10 mM TRIS, 100 mM KCl, pH 7.4) was pre-annealed by heating at 90°C and allowing to cool down to room temperature over 2h. DNA is then diluted to 5 µM final concentration in the presence of 1 equivalent of peptide. Melting and annealing temperatures were calculated from the first order derivative of a 10th order polynomial fitting function (custom program). Experiments where repeated after the addition of a 500 mM solution of LiCl to a final Li^+ concentration of 100 mM, in order to weaken the G4 structure.

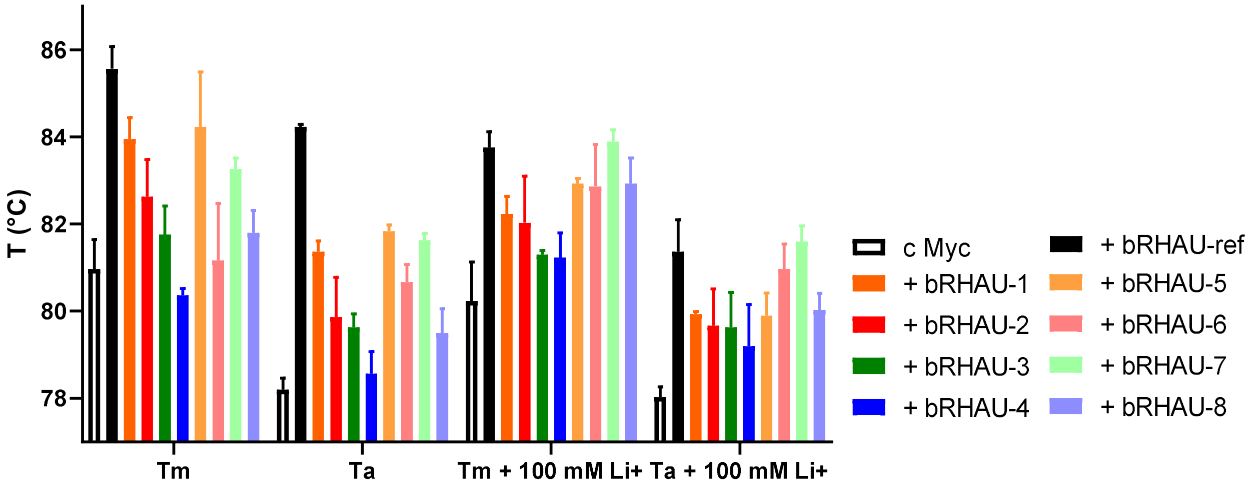

Fig. S31: melting and annealing temperature of cMYC DNA in presence of different peptides. Experiments were performed at 5 µM concentration of DNA and peptide in TRIS buffer pH 7.4.

8. Cyclization – RGD peptide

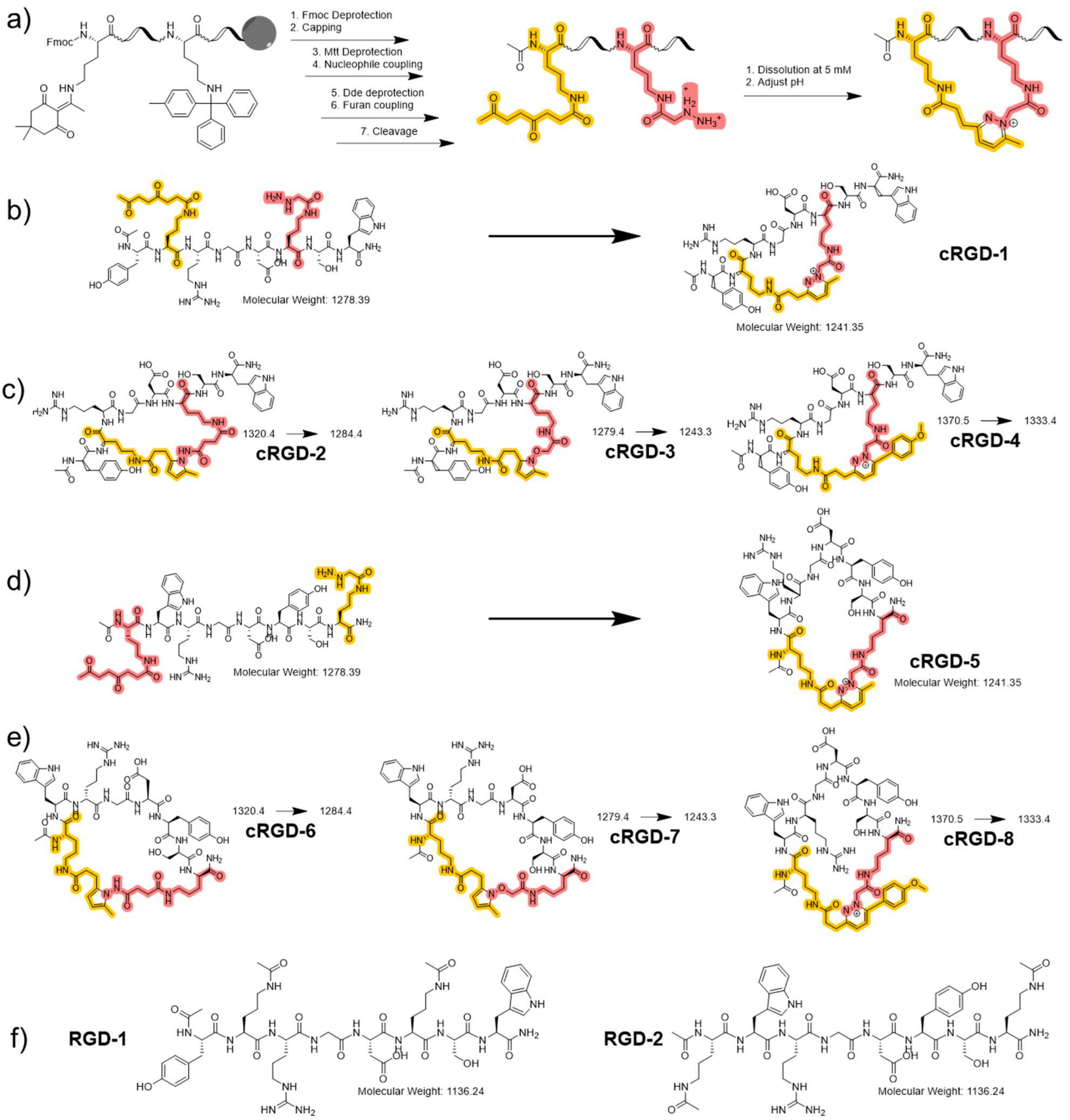

Fig. S32: a) general scheme of peptide functionalization and formation of cyclized products; b) structure of linear and cyclized (*i, i+4*) **cRGD-1**; (c) Structure of the other (*i,i+4*) macrocyclic peptides **cRGD-2**, **cRGD-3**, and **cRGD-4**; d) structure of linear and cyclized (*i, i+7*) **cRGD-5**; (e) Structure of the other (*i,i+7*) macrocyclic peptides **cRGD-6**, **cRGD-7**, and **cRGD-8;** f) structure of reference RGD peptides **RGD-1** and **RGD-2**.

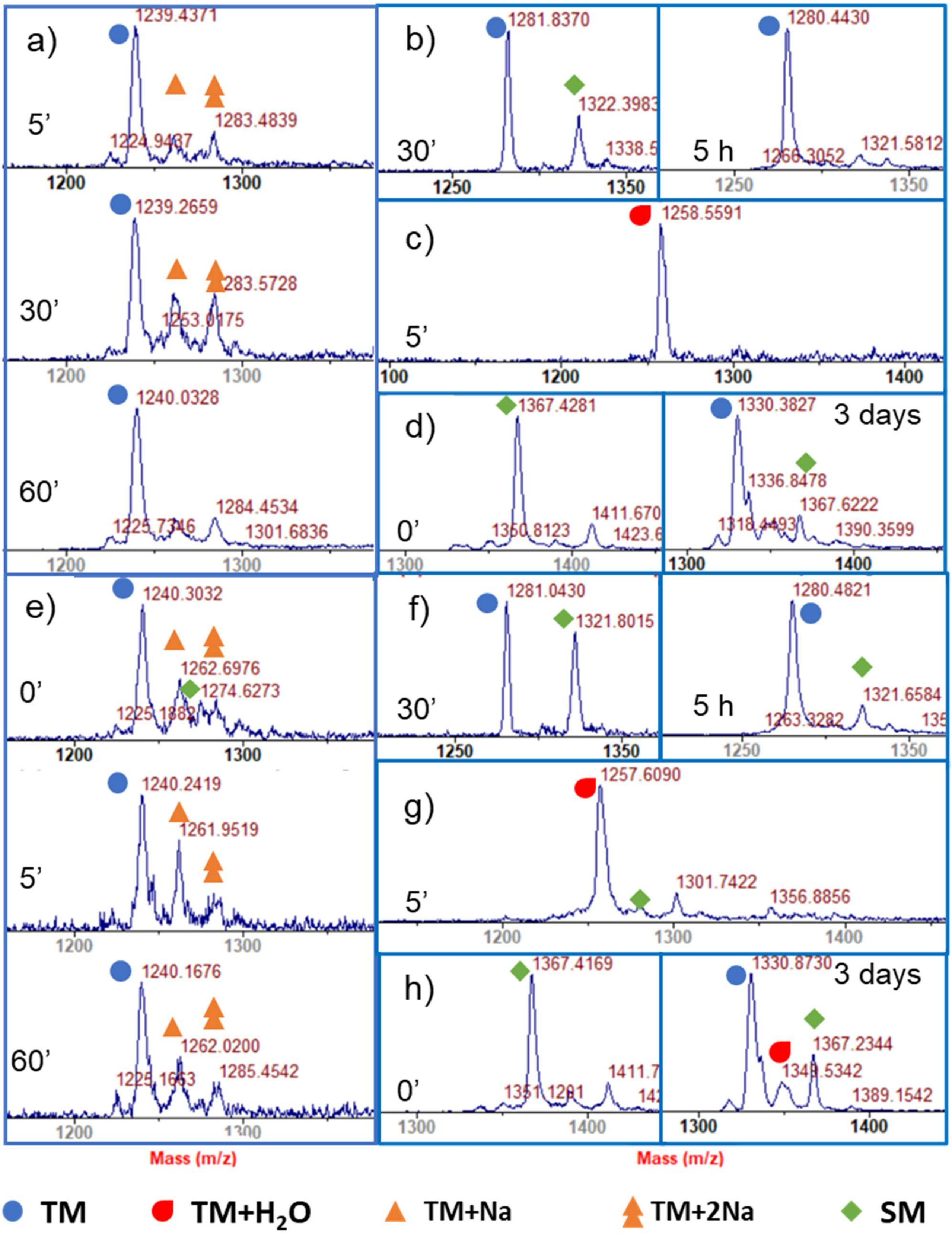

Fig. S33: MALDI analysis of the crude peptides after dissolution in mQ water at 5 mM concentration (0') and after specified time in pH adjusted solutions. a) **cRGD-1**, pH 7.5; b) **cRGD-2**, pH 2.5; c) **cRGD-3**, pH 2.5; d) **cRGD-4**, pH 7.5; e) **cRGD-5**, pH 7.5; f) **cRGD-6**, pH 2.5; g) **cRGD-7**, pH 2.5; g) **cRGD-8**, pH 7.5.

8.1 Trypsin stability

200 µL of buffered solution (PBS pH 7.4) containing peptides at 50 µM final concentration (from a 200 µM stock solution), were prepared in a 1.5 mL Eppendorf and allowed to equilibrate to 37°C. In a separate Eppendorf tube, Trypsin was equilibrated to 37°C at 10X concentration. After 5 minutes of equilibration, Trypsin was added to the peptides' solutions to 5.0 µg/mL (for *i,i+4* peptides) or 7.5 µg/mL (for *i,i+7* peptides) final concentration. At different time points, 20 µL of sample were mixed with 20 µL 1% TFA in mQ to inactivate the enzyme and analyzed via HPLC-UV.

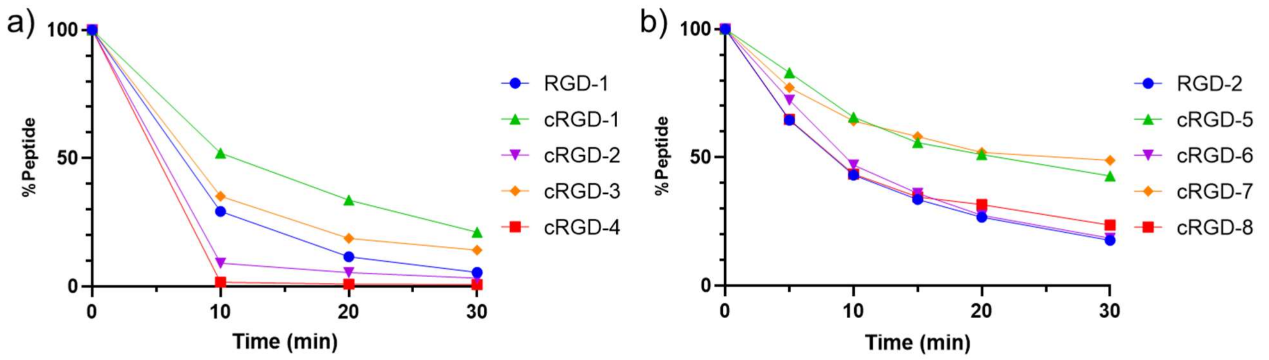

Fig. S34: degradation profile of the peptides in presence of trypsin. (a) relative degradation of (*i,i+4*) peptides compared to linear **RGD-1**; (b) relative degradation of (*i,i+7*) peptides compared to linear **RGD-2**. All experiments were conducted at 50 µM peptide concentration at 37°C.

8.2 Serum stability

200 µL of 20 µM peptides solutions (from a 200 µM stock) were prepared in 10% fetal bovine serum (FBS) in DMEM (*vide infra*, without phenol red) or PBS. Solutions were then incubated at 37°C for 24 h. At different time points, 30 µL of the sample were mixed with 70 µL quenching solution (1% TFA in MeCN containing 0.018 mM Fmoc-Val-OH as internal standard). Samples were immediately cooled down on ice bath for 5 minutes for protein precipitation, followed by centrifugation at 17,000 g for 10 min to remove the precipitated particles. Supernatants were then analyzed via HPLC-UV (Kinextex® C18 100 Å, 150 x 4.6 mm, 5 µm, at 35°C, flow rate 1.5 mL/min, 0-100% gradient of MeCN in water containing 0.1% TFA)

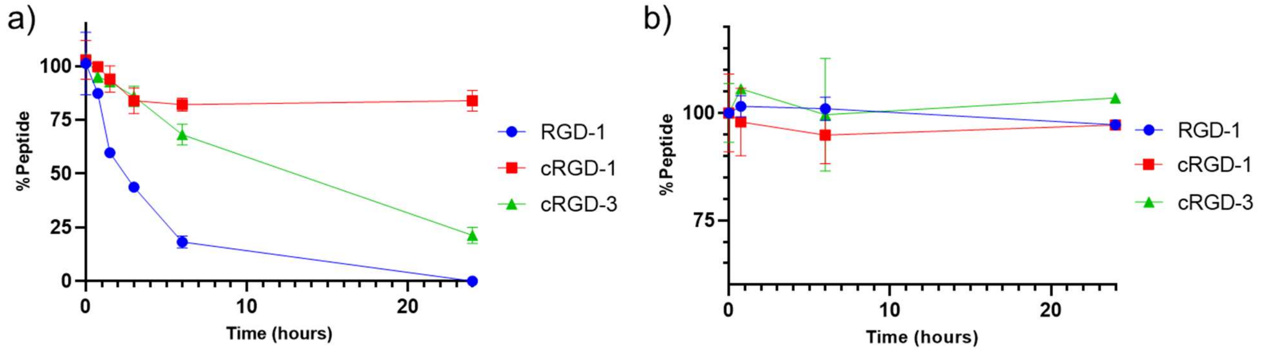

Fig. S35: degradation profile of the peptides in DMEM + 10% FBS (a) and PBS (b). All experiments were conducted at 20 µM peptide concentration at 37°C.

8.1 Cell adhesion assay

HeLa cells were grown at 37°C and 5% CO_2 in DMEM (containing pyruvate, glucose (4 g/L) and GlutaMax I from Invitrogen) that was additionally supplemented with 10% fetal bovine serum (FBS, TIBO Europe) and penicillin/streptomycin (Invitrogen).

The effect of the peptides on the cell adhesion in the absence or presence of FBS was determined by quantifying the residual adherent fraction using the XTT-proliferation assay (Roche, Sigma, kit II) in a 96-well plate. The optimal amount of cells to use per well for reliable quantification of adhesion (i.e. good S/N ratio, read

within dynamic range) was determined by prior testing to be 21000 cells/well. The cell adhesion assay[7] used flat-bottom 96-well plates coated with an RGD-containing extracellular matrix protein as follows: 50 µL of 30 µg/mL human fibronectin (Sigma) in PBS was added per well and removed after 60 minutes incubation at 37°C. HeLa cells, collected from a sub-confluent culture using 0.25% w/v trypsin/1mM EDTA were washed three times in FBS-free DMEM. The final HeLa cell suspension (either in FBS-free medium or in 10%-FBS medium) and the tested peptide (in water) were first pre-incubated in solution in a 1/2.33 v/v ratio (e.g., 140 µL cell suspension/60 µL peptide solution) for 10 minutes at 37°C 5% CO_2 with continuous slow rotation (100 rpm). The final peptide concentration was 60 µM. Subsequently, 200 µL of the preincubated cell/peptide mixture containing 21000 cells was added each fibronectin-coated well in the 96-well plate. 3-6 replicate wells were used per condition; blank samples contained no cells and no peptide and control samples containing cells but no peptide. The cells were allowed to adhere to the well during either 45 or 90 minutes at 37°C, 5% CO_2. Non-attached cells were subsequently removed and the residual adherent fraction was quantified by XTT-assay.[8,9] In brief, 50 µL XTT-solution (containing the electron coupling reagent and the labelling reagent in a 1:50 ratio) and 100 µL FBS-free medium were added per well. During the subsequent 3h reaction at 37° C the metabolically active cells of the residual adherent cell fraction converted XTT to the colored soluble formazan of which absorbance was measured at 450 nm using the iMark microplate reader. The obtained absorption was corrected for background measurement at 595 nm and for the mean blank value (from replicate wells without cells). The final data are presented as the mean % of cell adhesion normalized to that of the control sample (with cells, no peptide,). The data were compared using one-way ANOVA followed by Dunnett's multiple comparisons test in GraphPad Prism 9.5.0.

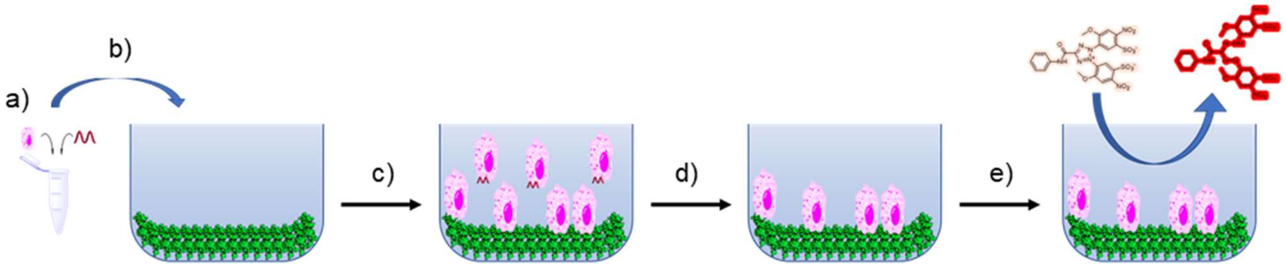

Fig. S36: general scheme of cellular adhesion assays. (a) pre-incubation of tested peptide with HeLa cells for 10 minutes; (b) transfer to fibronectin-coated wells; (c) incubation for 45 minutes; (d) wash of unadhered cells; (e) evaluation of adhered fraction based on XTT reduction.

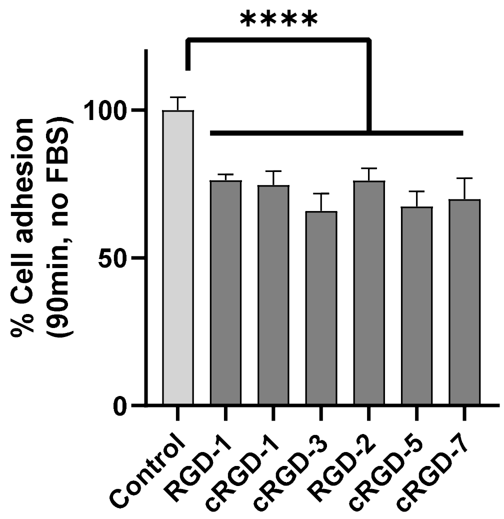

Fig. S37: Effect on cell adhesion level of 60 µM of RGD reference peptides and cRGD peptides of HeLa cells after 90 minutes in culture medium not containing FBS. The adhesion level is expressed as % (relative to control where no peptide is added to the cells). For peptide labels see Table S1 and Figure S31. ****: $p<0.0001$

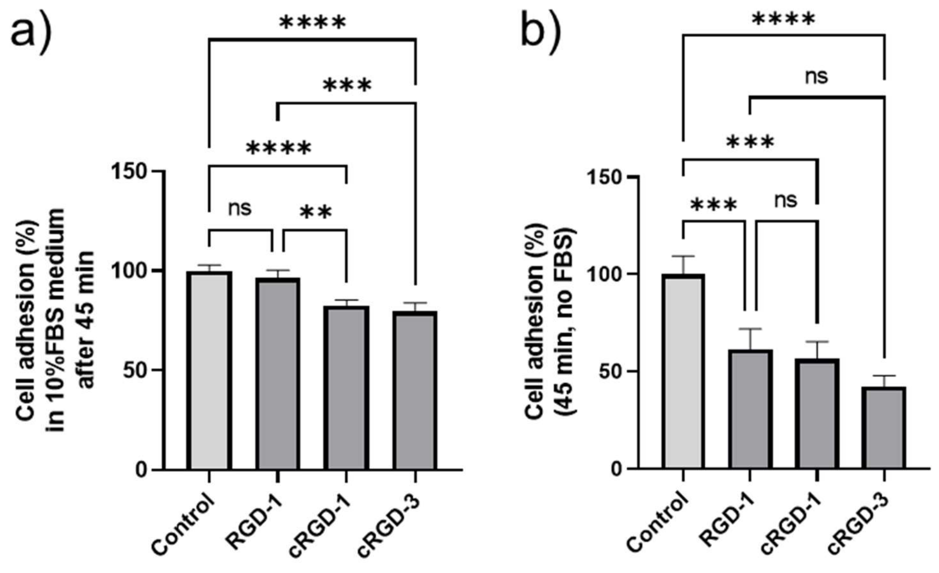

Fig. S38: Effect on cell adhesion level of 60 μM of RGD reference peptides and cRGD peptides of HeLa cells after 45 minutes in culture medium containing FBS (a) or not (b). The adhesion level is expressed as % (relative to control where no peptide is added to the cells). For peptide labels see Table S1 and Figure S31. ns: not significant, ** < 0.01, *** < 0.0001 **** p <0.0001.

9. References

(1) Carmody, W. R. An Easily Prepared Wide Range Buffer Series. *J. Chem. Educ.* **1963**, *40* (5), A386. https://doi.org/10.1021/ed040pA386.1.

(2) Manicardi, A.; Cadoni, E.; Madder, A. Visible-Light Triggered Templated Ligation on Surface Using Furan-Modified PNAs. *Chem. Sci.* **2020**, *11* (43), 11729–11739. https://doi.org/10.1039/D0SC04875E.

(3) Manicardi, A.; Cadoni, E.; Madder, A. Hydrolysis of 5-Methylfuran-2-Yl to 2,5-Dioxopentanyl Allows for Stable Bio-Orthogonal Proximity-Induced Ligation. *Commun. Chem.* **2021**, *4* (1), 146. https://doi.org/10.1038/s42004-021-00584-1.

(4) Manicardi, A.; Accetta, A.; Tedeschi, T.; Sforza, S.; Marchelli, R.; Corradini, R. PNA Bearing 5-Azidomethyluracil. *Artif. DNA PNA XNA* **2012**, *3* (2), 53–62. https://doi.org/10.4161/adna.20158.

(5) Chouikhi, D.; Ciobanu, M.; Zambaldo, C.; Duplan, V.; Barluenga, S.; Winssinger, N. Expanding the Scope of PNA-Encoded Synthesis (PES): Mtt-Protected PNA Fully Orthogonal to Fmoc Chemistry and a Broad Array of Robust Diversity-Generating Reactions. *Chem. - A Eur. J.* **2012**, *18* (40), 12698–12704. https://doi.org/10.1002/chem.201201337.

(6) Díaz-Mochón, J. J.; Bialy, L.; Bradley, M. Full Orthogonality between Dde and Fmoc: The Direct Synthesis of PNA-Peptide Conjugates. *Org. Lett.* **2004**, *6* (7), 1127–1129. https://doi.org/10.1021/ol049905y.

(7) Sheu, J. R.; Lin, C. H.; Peng, H. C.; Huang, T. F. Triflavin, an Arg-Gly-Asp-Containing Peptide, Inhibits Human Cervical Carcinoma (HeLa) Cell-Substratum Adhesion through an RGD-Dependent Mechanism. *Peptides* **1994**, *15* (8), 1391–1398. https://doi.org/10.1016/0196-9781(94)90114-7.

(8) Scudiero, D. A.; Shoemaker, R. H.; Paull, K. D.; Monks, A.; Tierney, S.; Nofziger, T. H.; Currens, M. J.; Seniff, D.; Boyd, M. R. Evaluation of a Soluble Tetrazolium/Formazan Assay for Cell Growth and Drug Sensitivity in Culture Using Human and Other Tumor Cell Lines. *Cancer Res.* **1988**, *48* (17), 4827–4833.

(9) Huyck, L.; Ampe, C.; Van Troys, M. The XTT Cell Proliferation Assay Applied to Cell Layers Embedded in Three-Dimensional Matrix. *Assay Drug Dev. Technol.* **2012**, *10* (4), 382–392. https://doi.org/10.1089/adt.2011.391.

10. HPLC-MS chromatograms of purified peptides

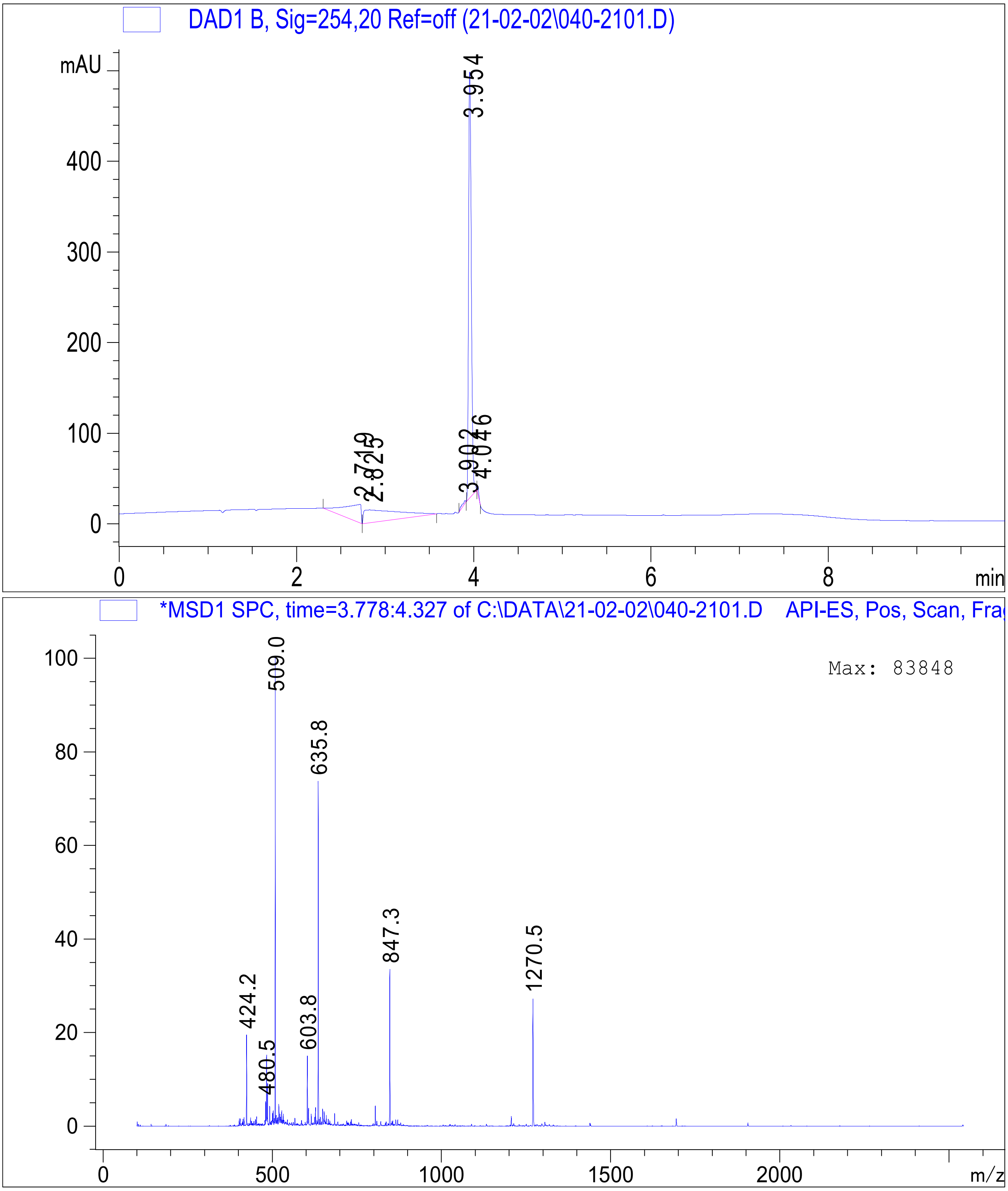

Fig. S39: HPLC-MS chromatogram of purified **Coil-Nu2**. HPLC-UV trace at 254 nm (top) and MS spectrum of the corresponding peak (bottom). Calcd MW: 2540.0. For chromatographic conditions, please refer to the general information section.

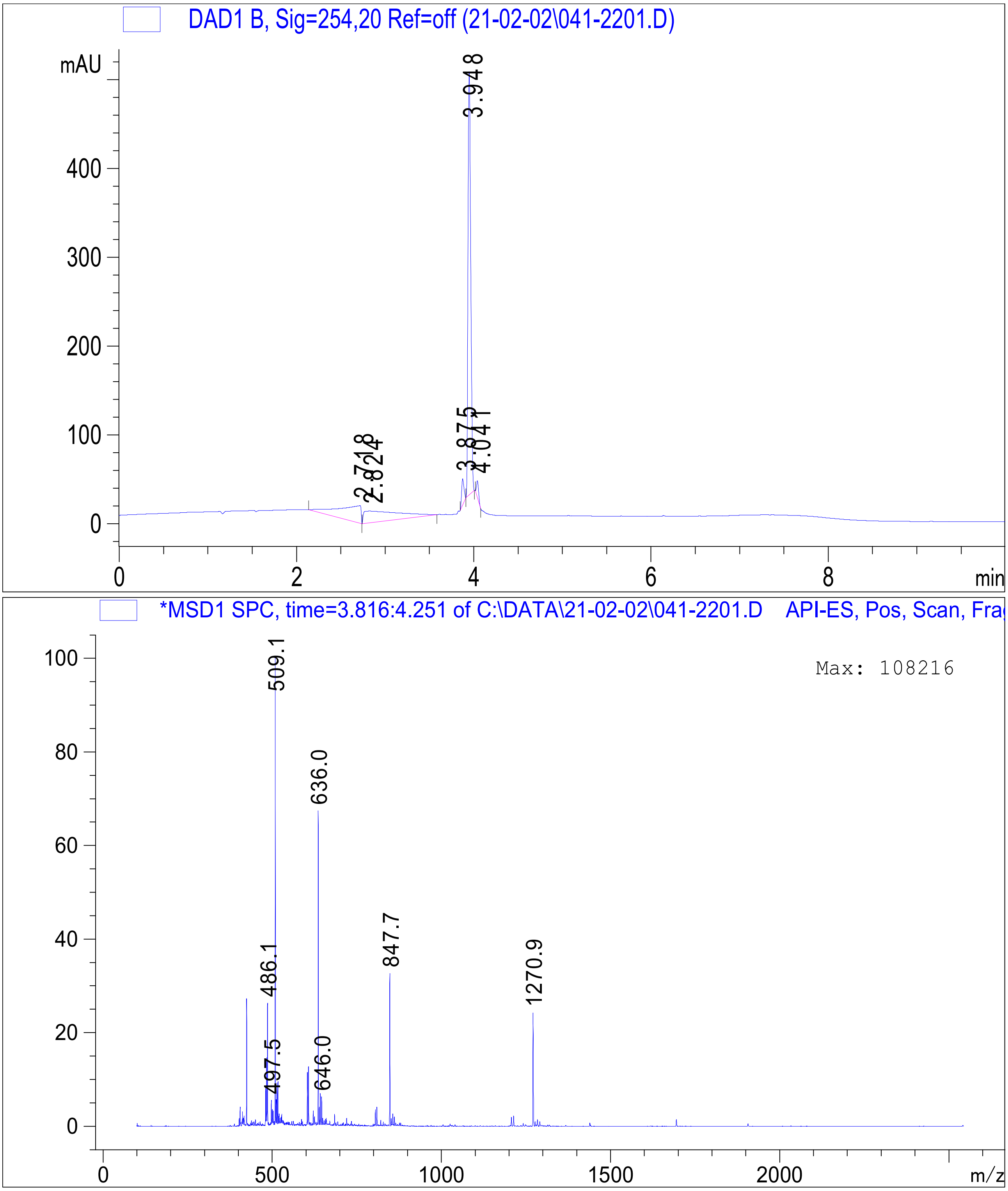

Fig. S40: HPLC-MS chromatogram of purified **Coil-Nu3**. HPLC-UV trace at 254 nm (top) and MS spectrum of the corresponding peak (bottom). Calcd MW: 2541.0. For chromatographic conditions, please refer to the general information section.

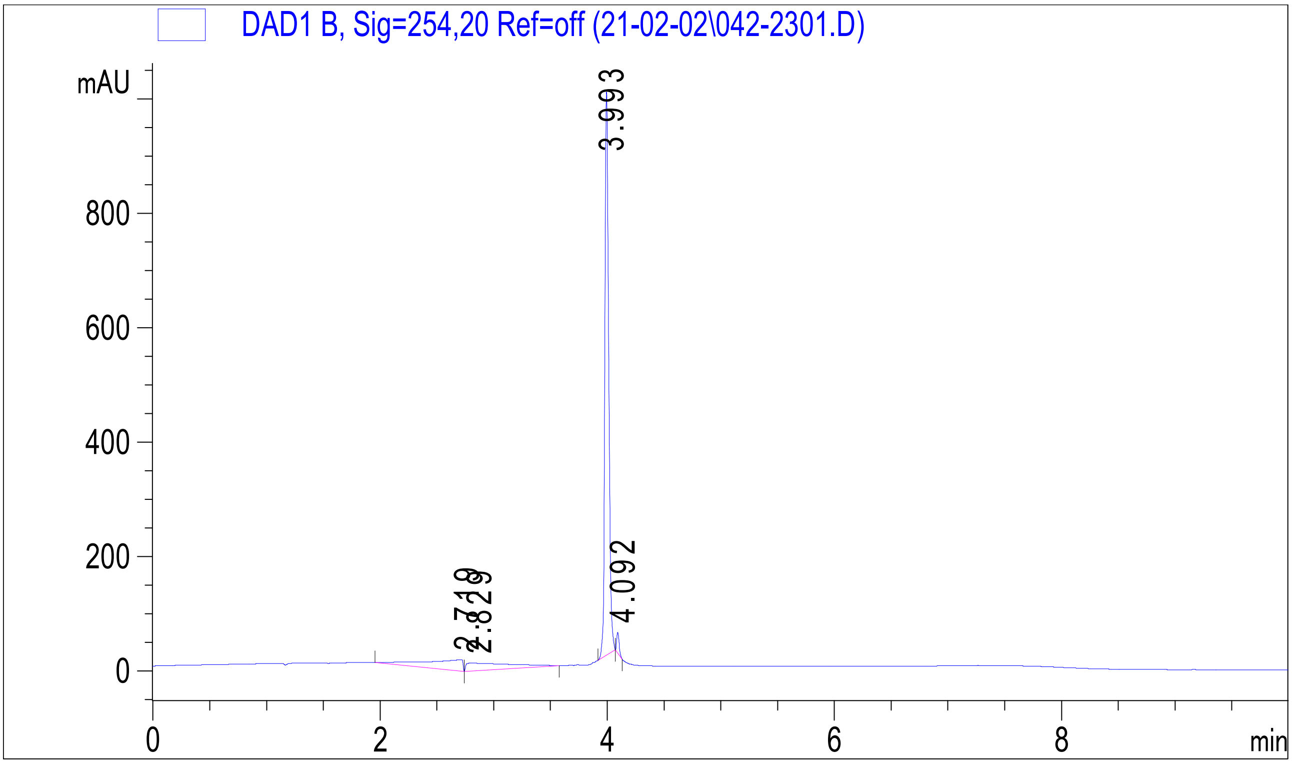

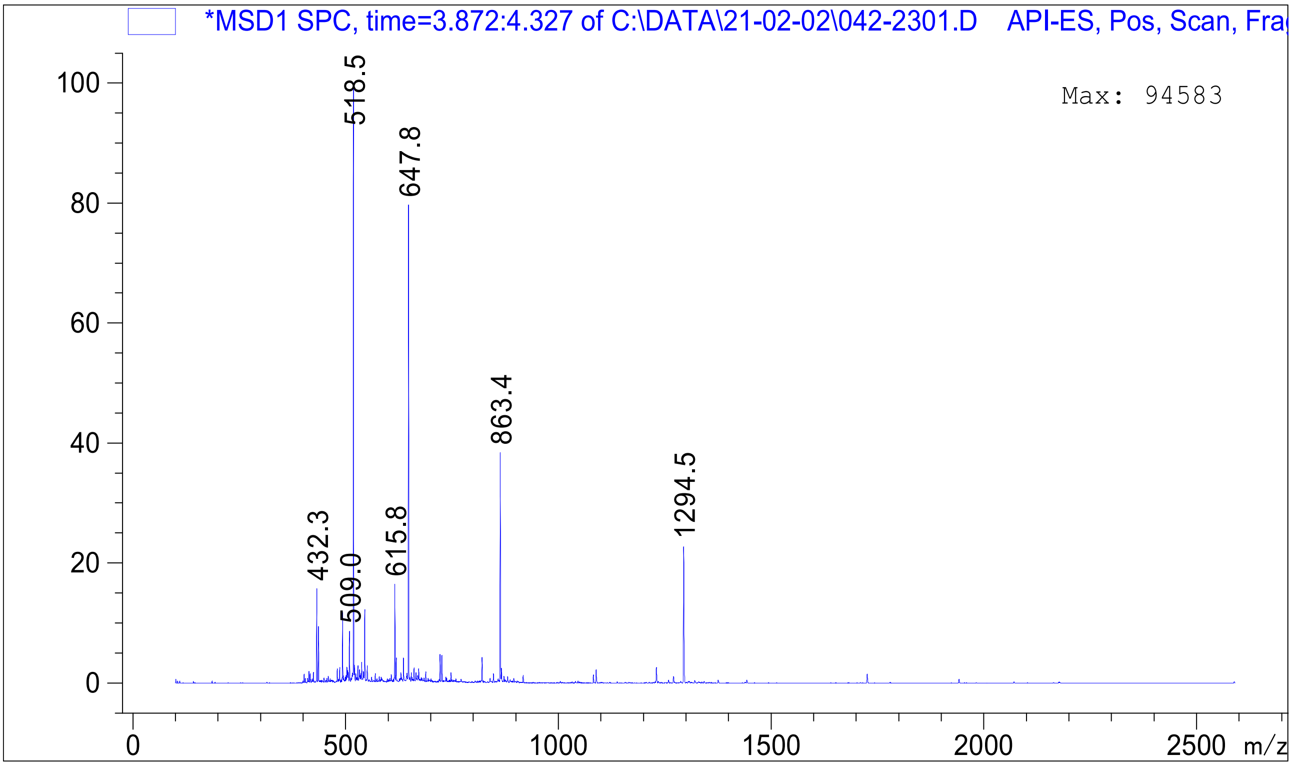

Fig. S41: HPLC-MS chromatogram of purified **Coil-Nu4**. HPLC-UV trace at 254 nm (top) and MS spectrum of the corresponding peak (bottom). Calcd MW: 2588.1. For chromatographic conditions, please refer to the general information section.

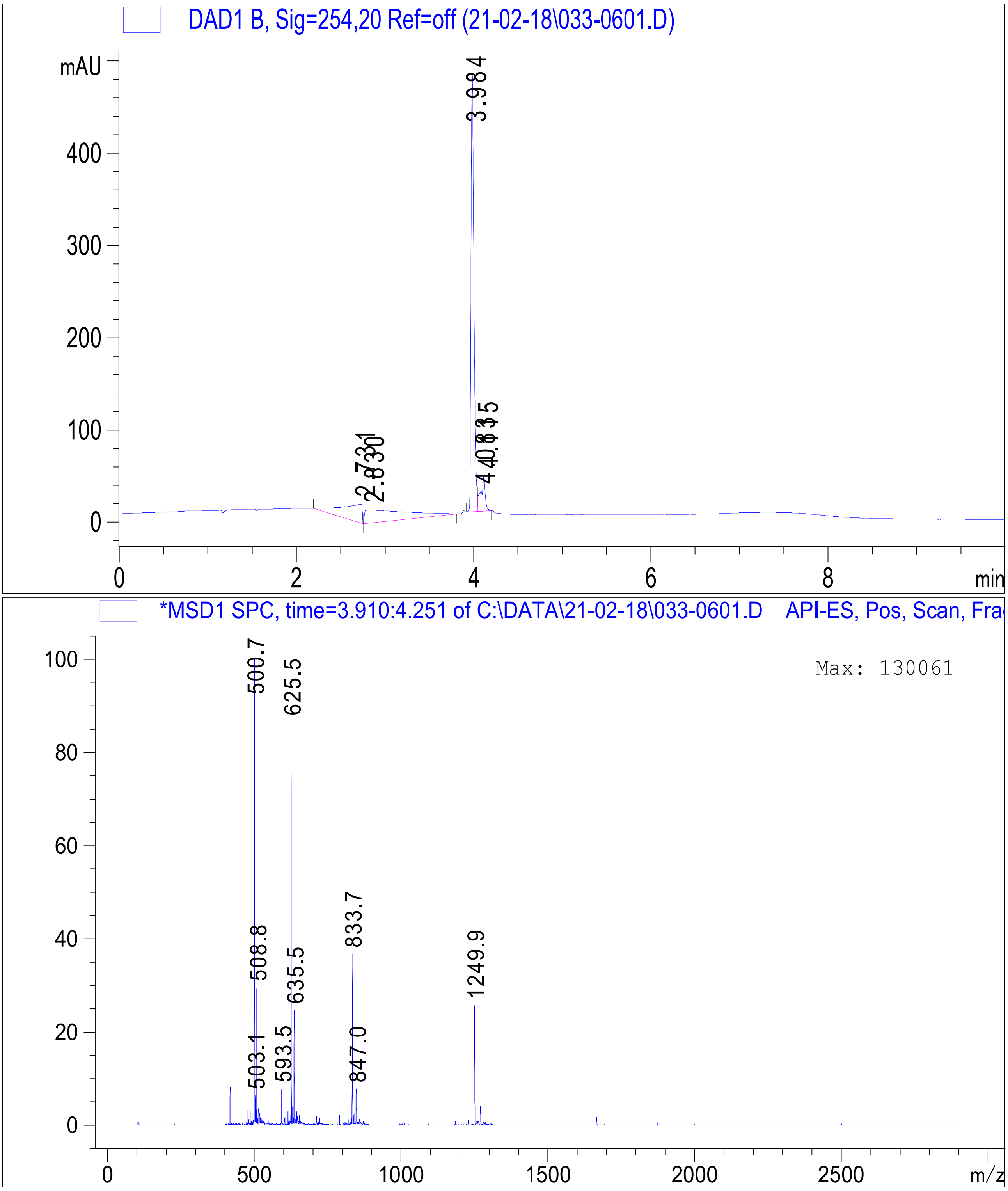

Fig. S42: HPLC-MS chromatogram of purified **Coil-Nu5**. HPLC-UV trace at 254 nm (top) and MS spectrum of the corresponding peak (bottom). Calcd MW: 2499.0. For chromatographic conditions, please refer to the general information section.

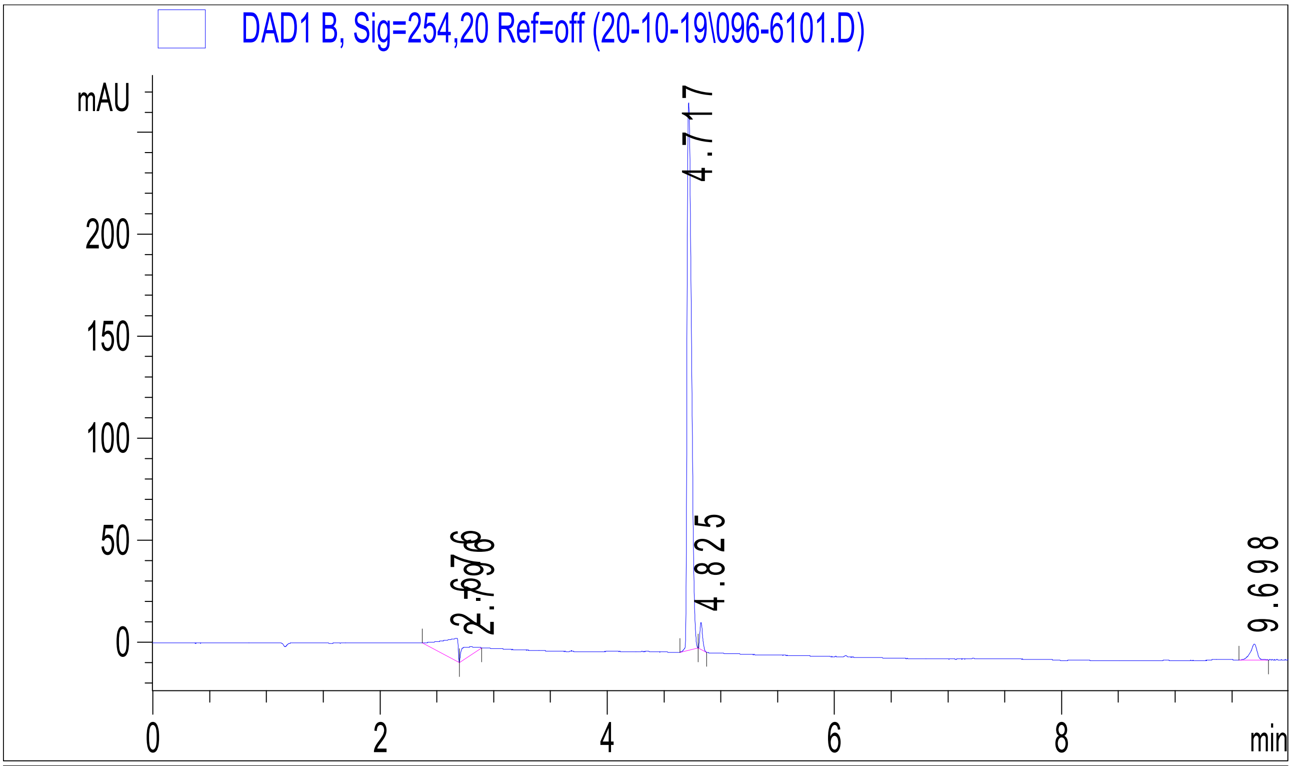

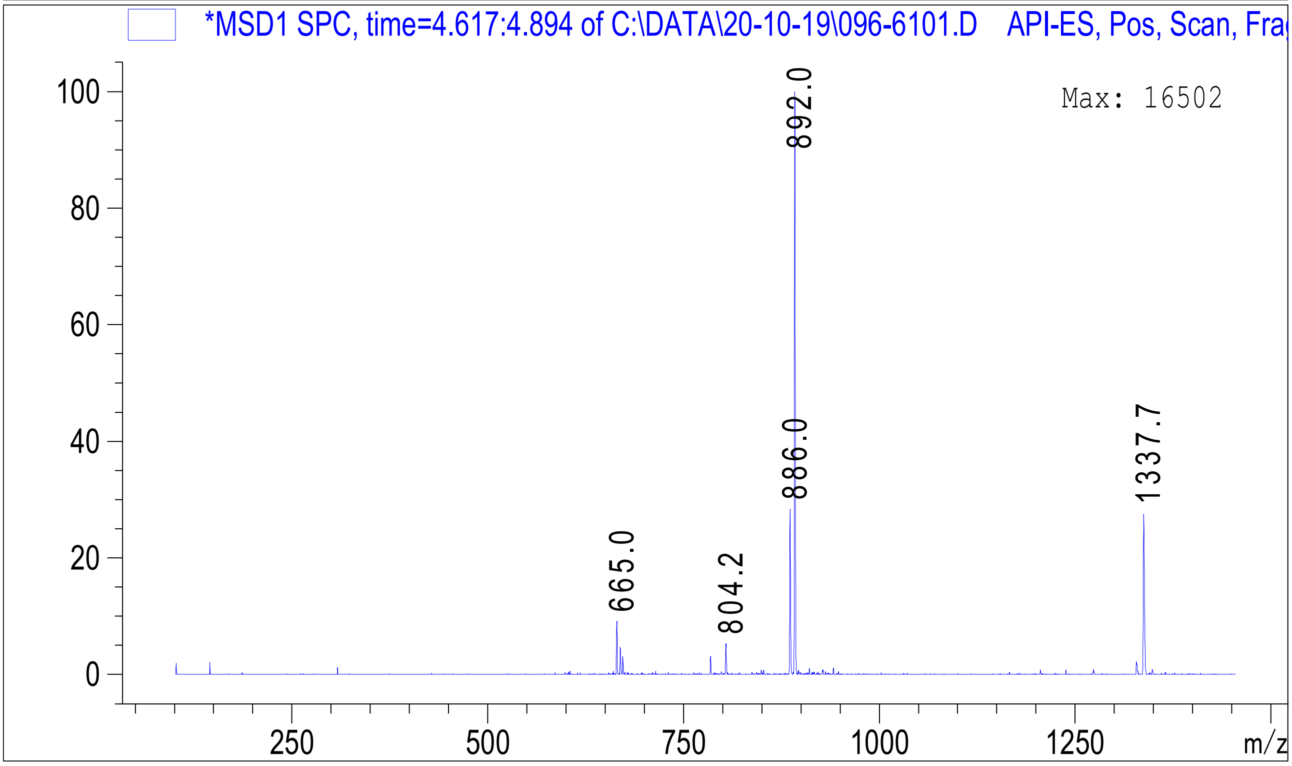

Fig. S43: HPLC-MS chromatogram of purified **Coil-DOP2**. HPLC-UV trace at 254 nm (top) and MS spectrum of the corresponding peak (bottom). Calcd MW: 2674.1 For chromatographic conditions, please refer to the general information section.

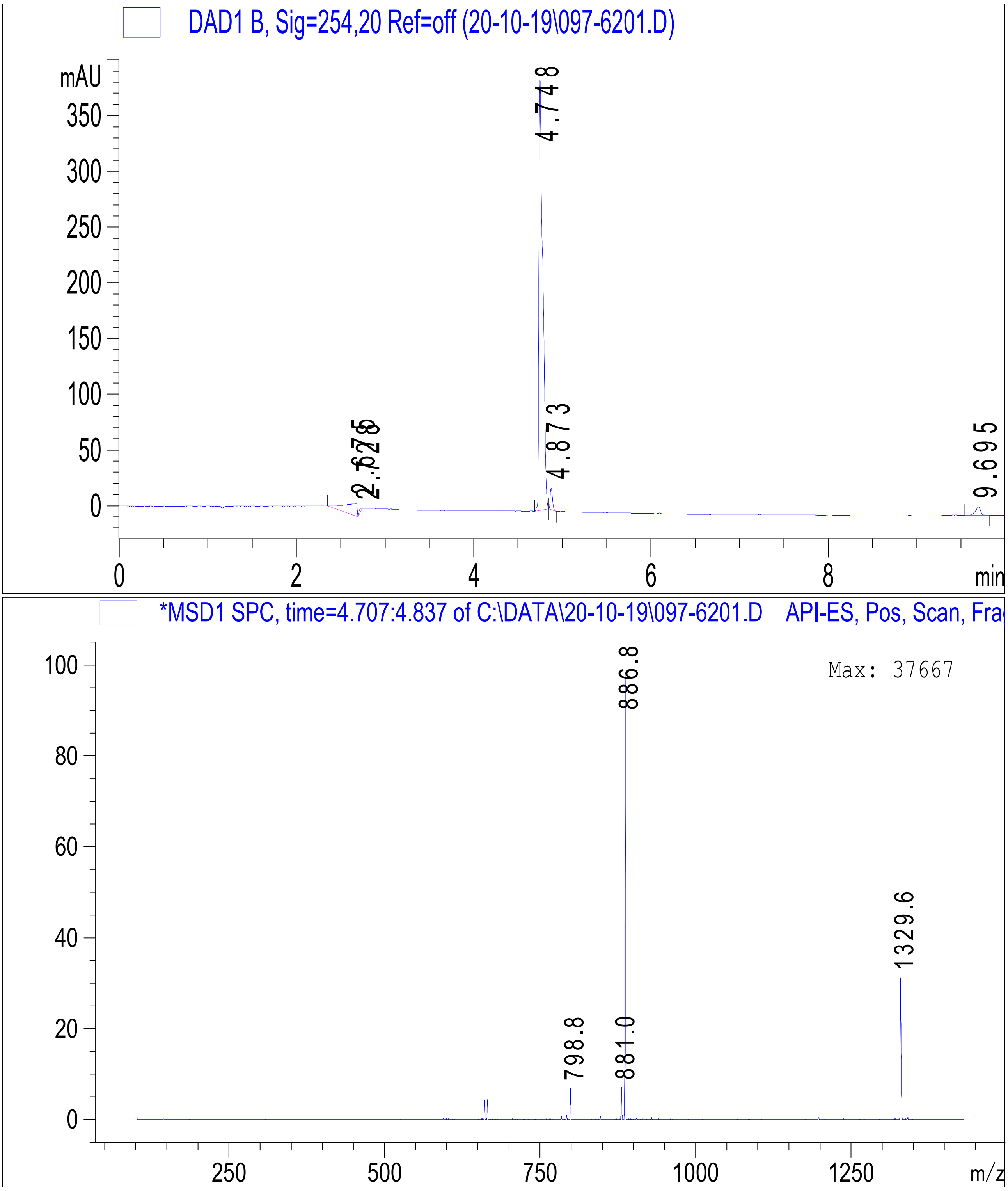

Fig. S44: HPLC-MS chromatogram of purified **Coil-DOP3**. HPLC-UV trace at 254 nm (top) and MS spectrum of the corresponding peak (bottom). Calcd MW: 2658.1. For chromatographic conditions, please refer to the general information section.

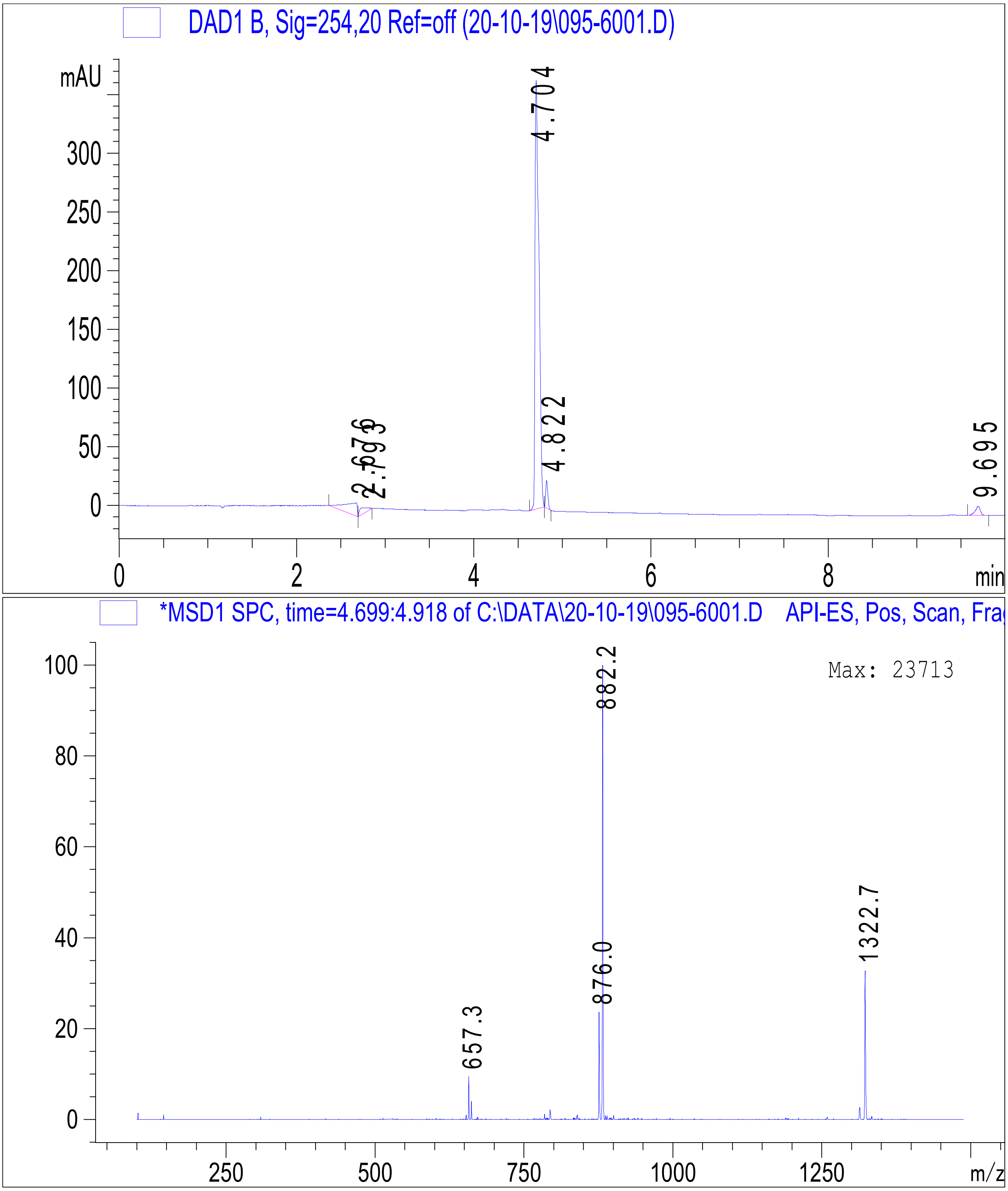

Fig. S45: HPLC-MS chromatogram of purified **Coil-DOP4**. HPLC-UV trace at 254 nm (top) and MS spectrum of the corresponding peak (bottom). Calcd MW: 2644.0. For chromatographic conditions, please refer to the general information section.

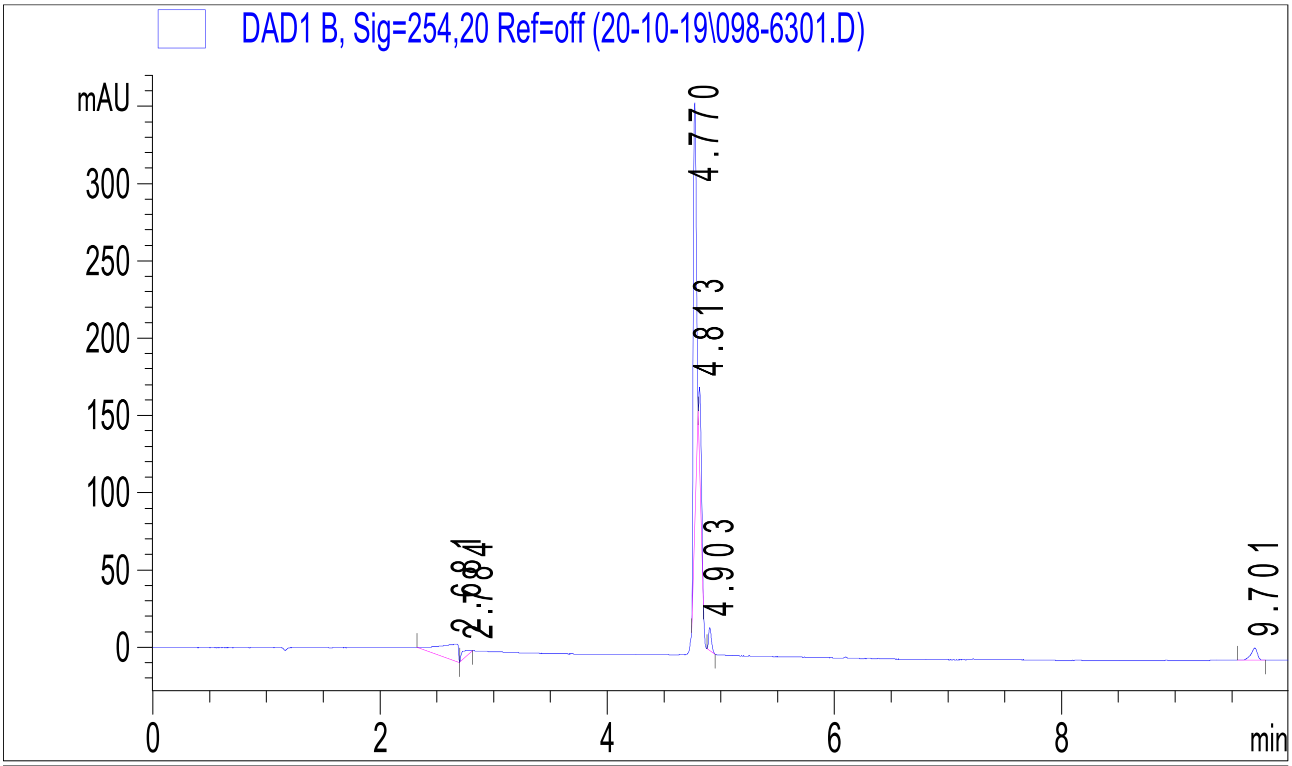

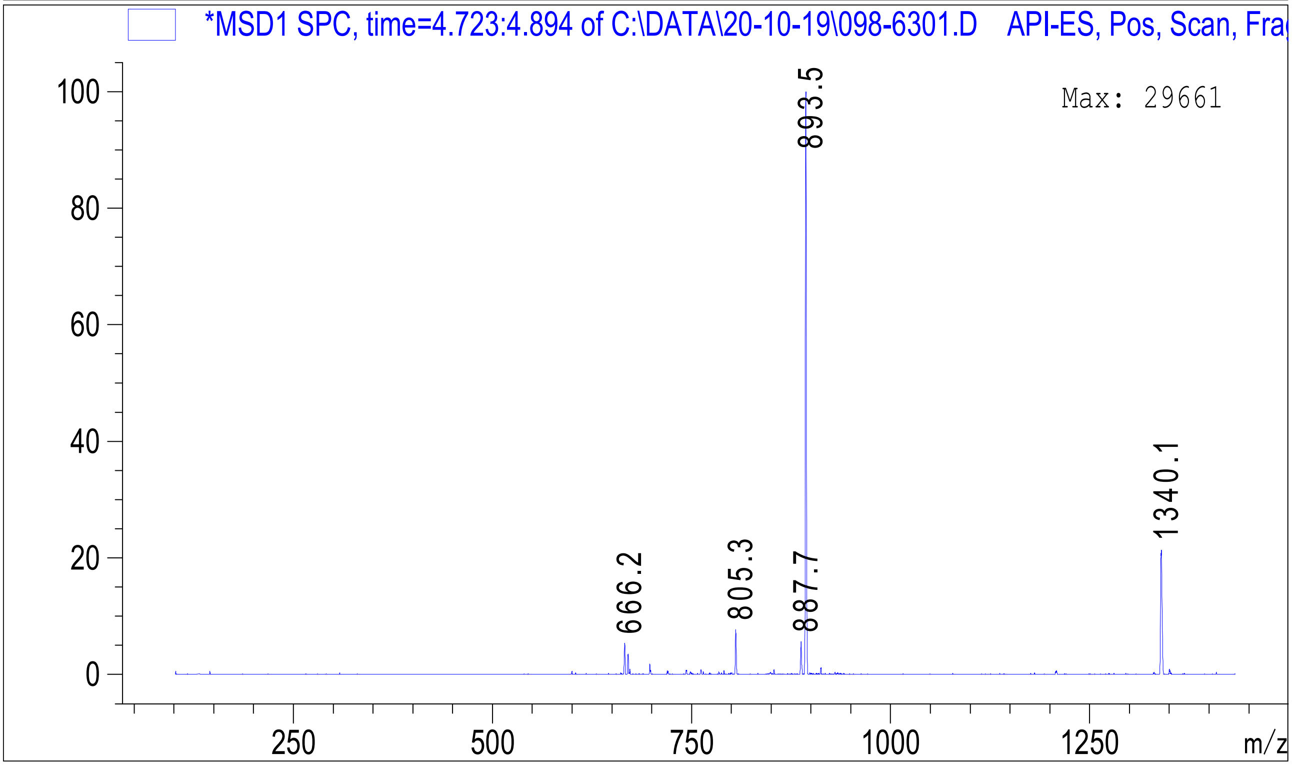

Fig. S46: HPLC-MS chromatogram of purified **Coil-DOP5**. HPLC-UV trace at 254 nm (top) and MS spectrum of the corresponding peak (bottom). Calcd MW: 2678.5. For chromatographic conditions, please refer to the general information section.

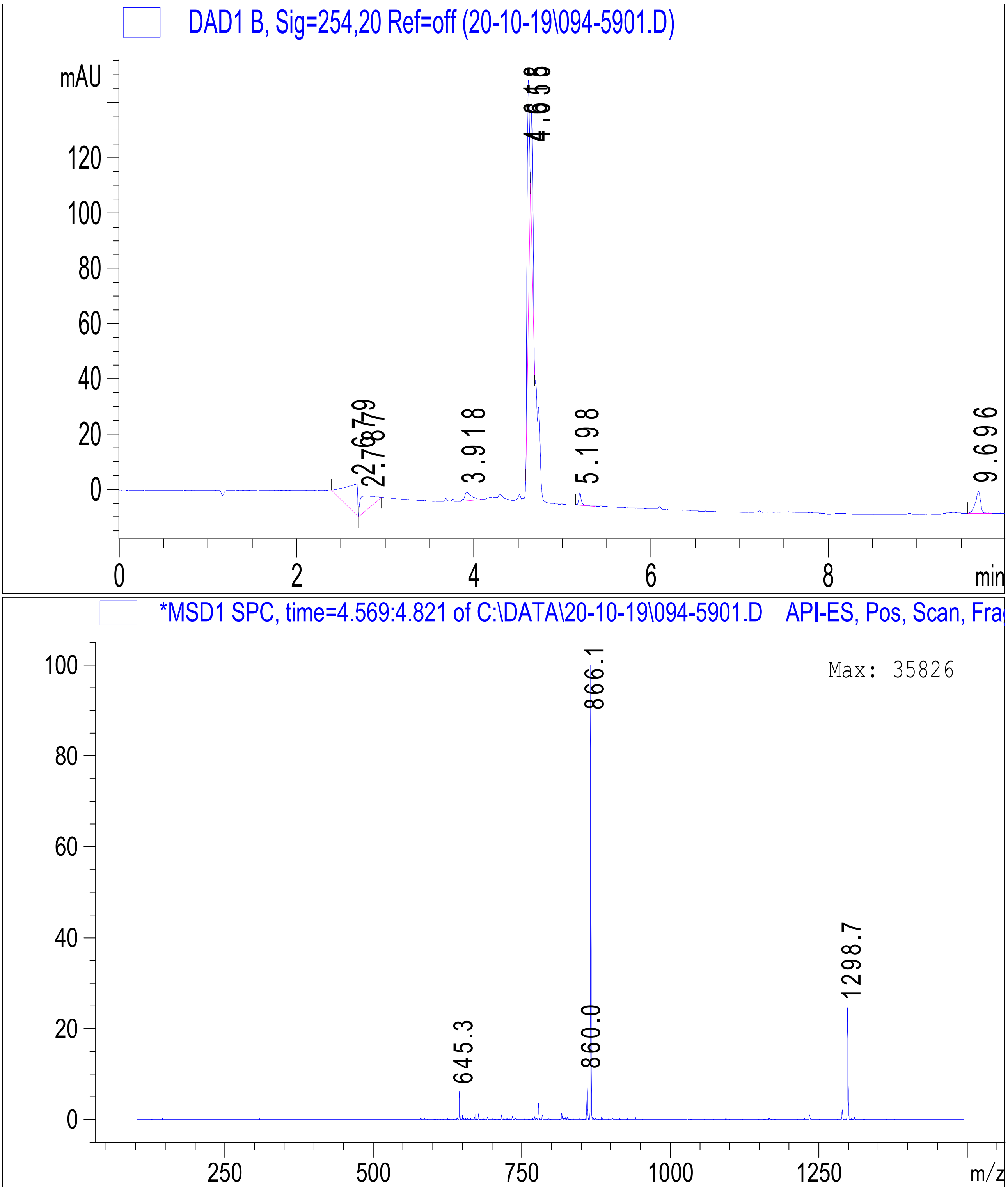

Fig. S47: HPLC-MS chromatogram of purified **Coil-DOP6**. HPLC-UV trace at 254 nm (top) and MS spectrum of the corresponding peak (bottom). Calcd MW: 2596.0. For chromatographic conditions, please refer to the general information section.

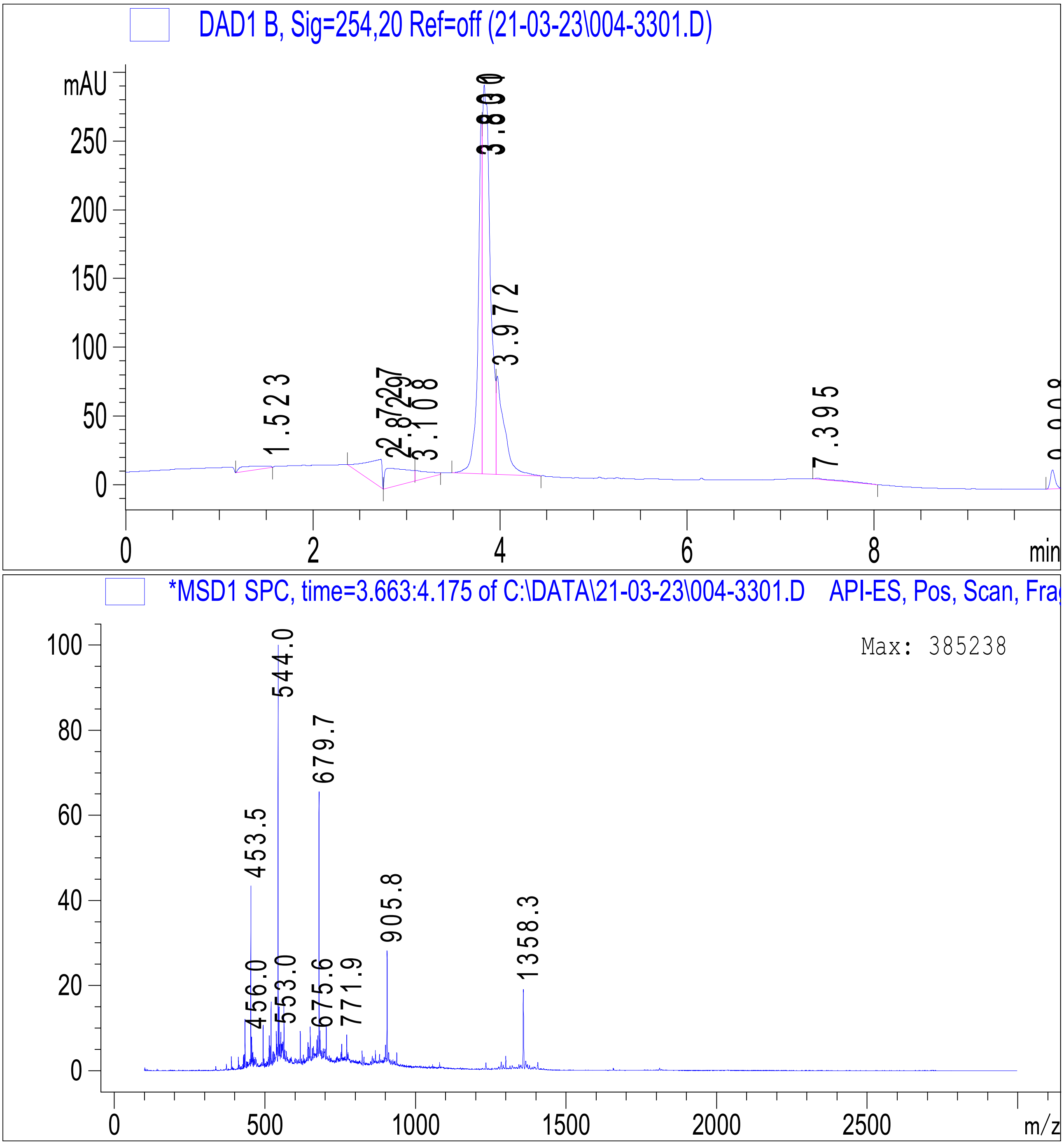

Fig. S48: HPLC-MS chromatogram of purified **Coil-A**. HPLC-UV trace at 254 nm (top) and MS spectrum of the corresponding peak (bottom). Calcd MW: 2715.4. For chromatographic conditions, please refer to the general information section.

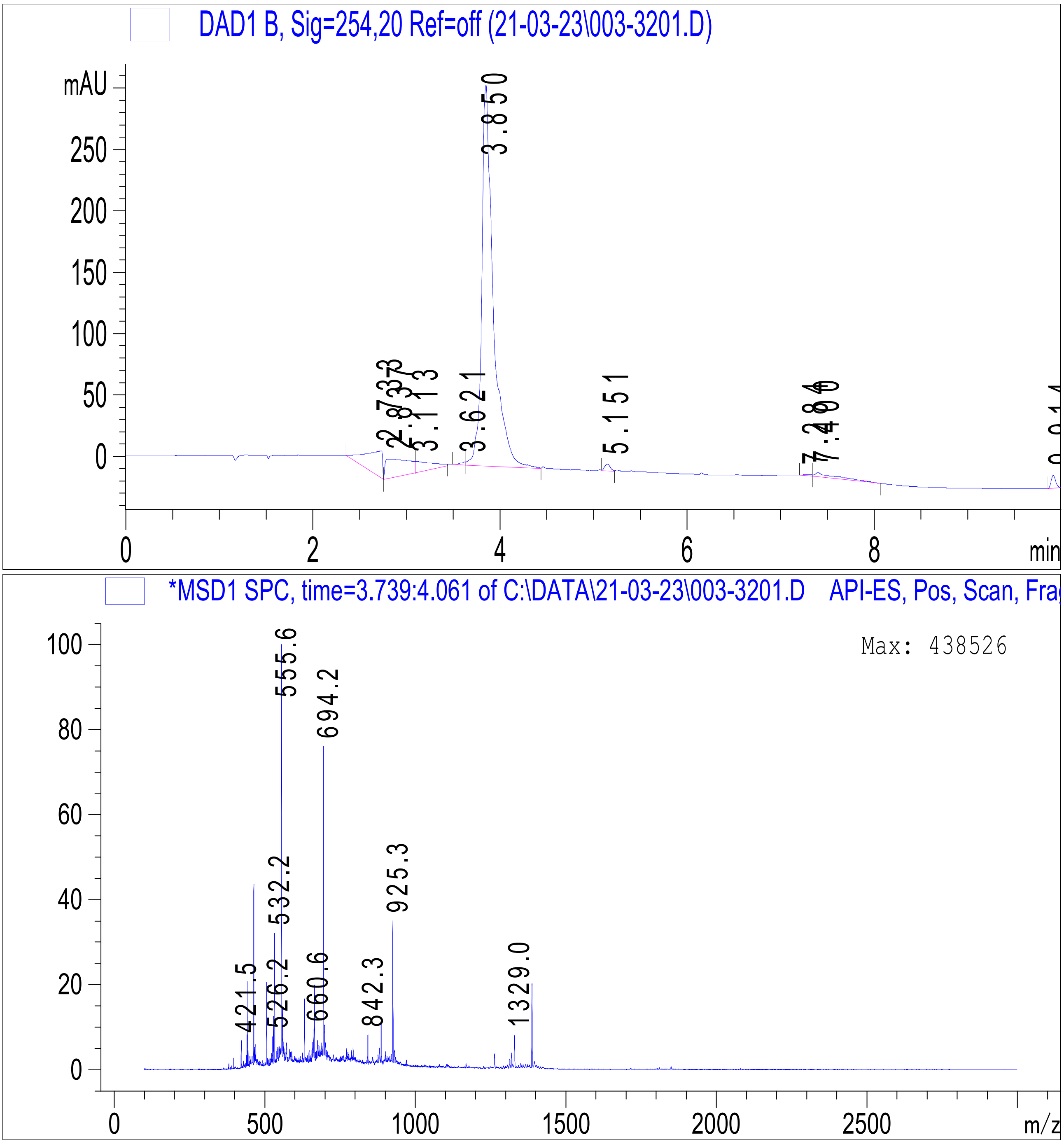

Fig. S49: HPLC-MS chromatogram of purified **Coil-B**. HPLC-UV trace at 254 nm (top) and MS spectrum of the corresponding peak (bottom). Calcd MW: 2773.4. For chromatographic conditions, please refer to the general information section.

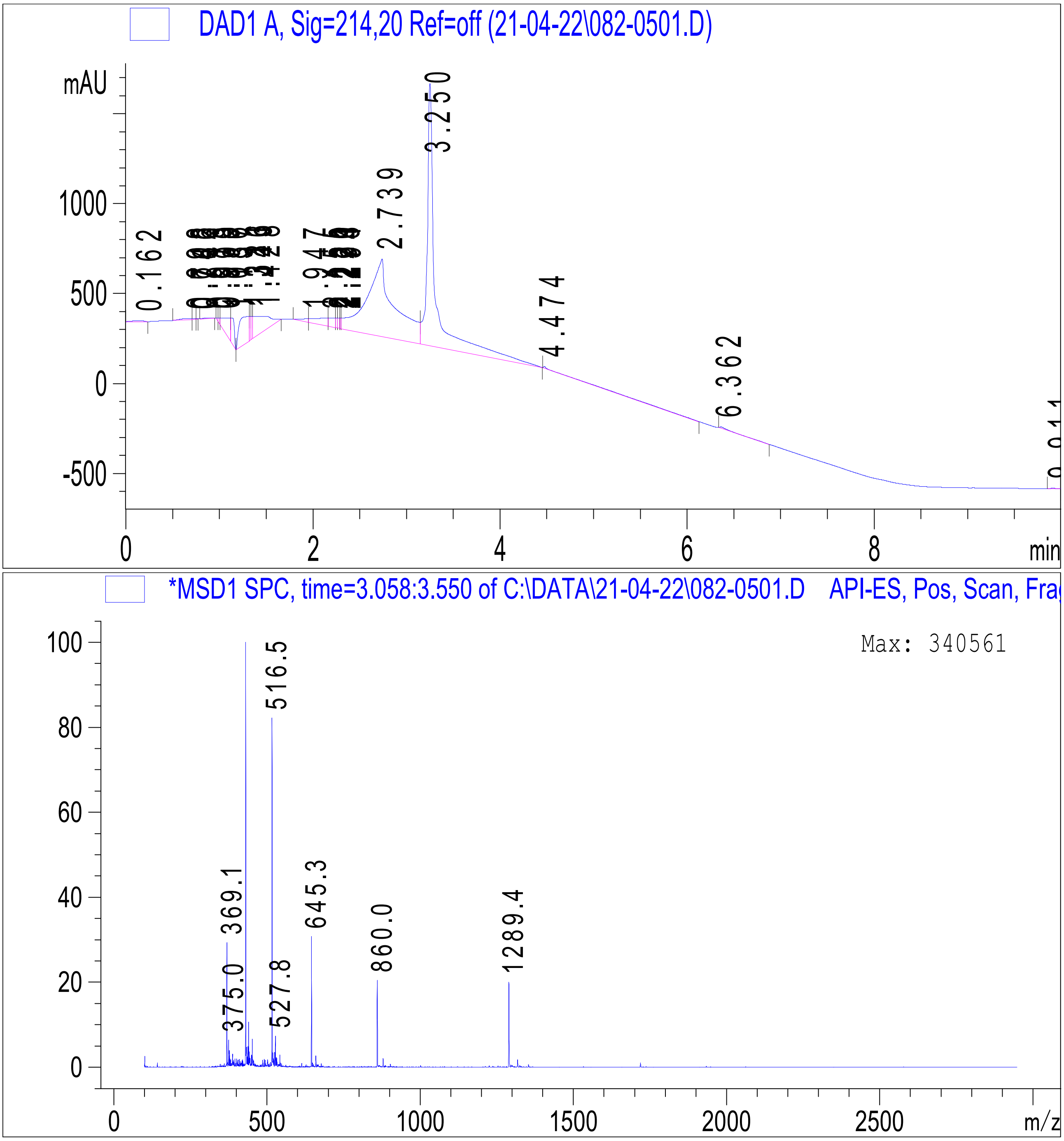

Fig. S50: HPLC-MS chromatogram of purified **bRHAU.** HPLC-UV trace at 214 nm (top) and MS spectrum of the corresponding peak (bottom). Calcd MW: 2578.0. For chromatographic conditions, please refer to the general information section.

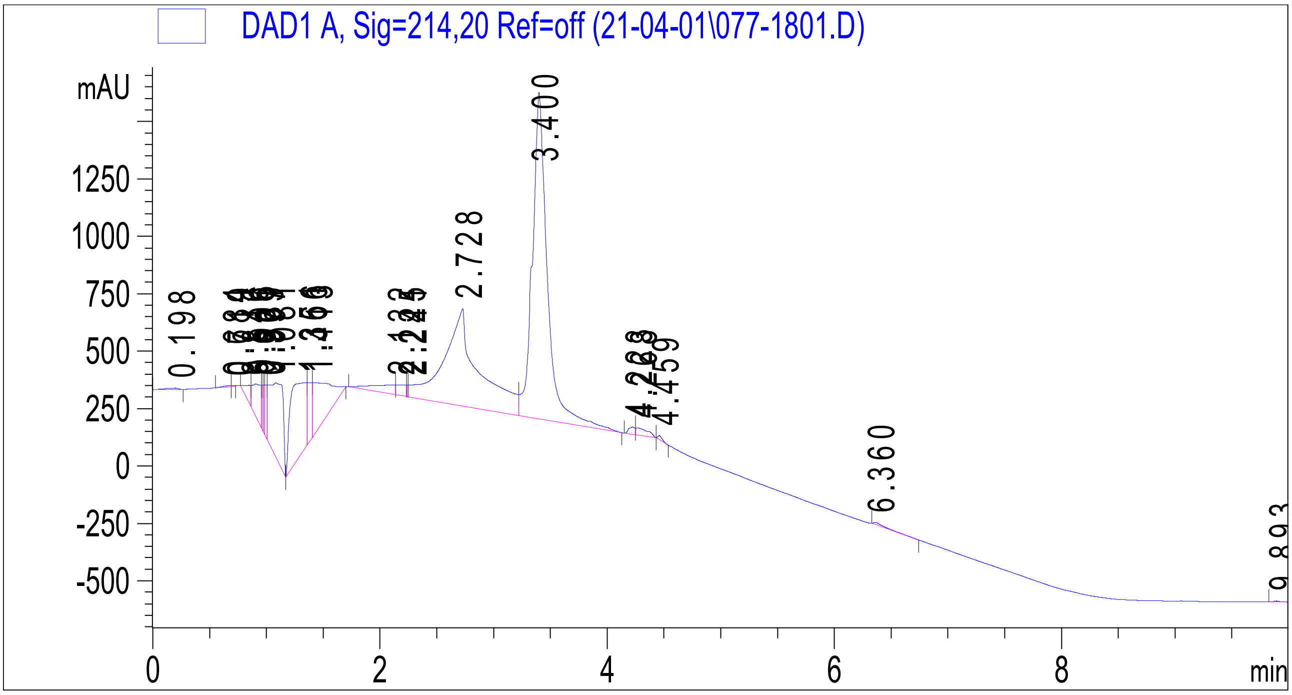

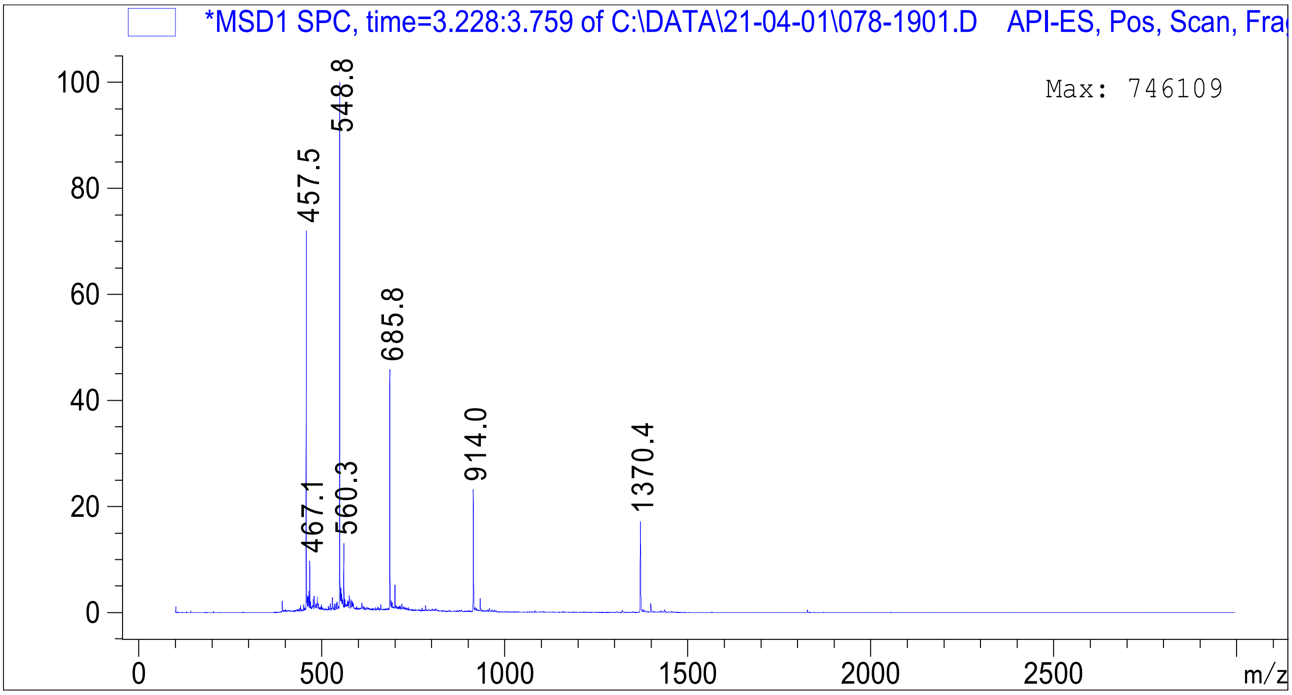

Fig. S51: HPLC-MS chromatogram of purified **bRHAU-1**. HPLC-UV trace at 214 nm (top) and MS spectrum of the corresponding peak (bottom). Calcd MW: 2739.3. For chromatographic conditions, please refer to the general information section.

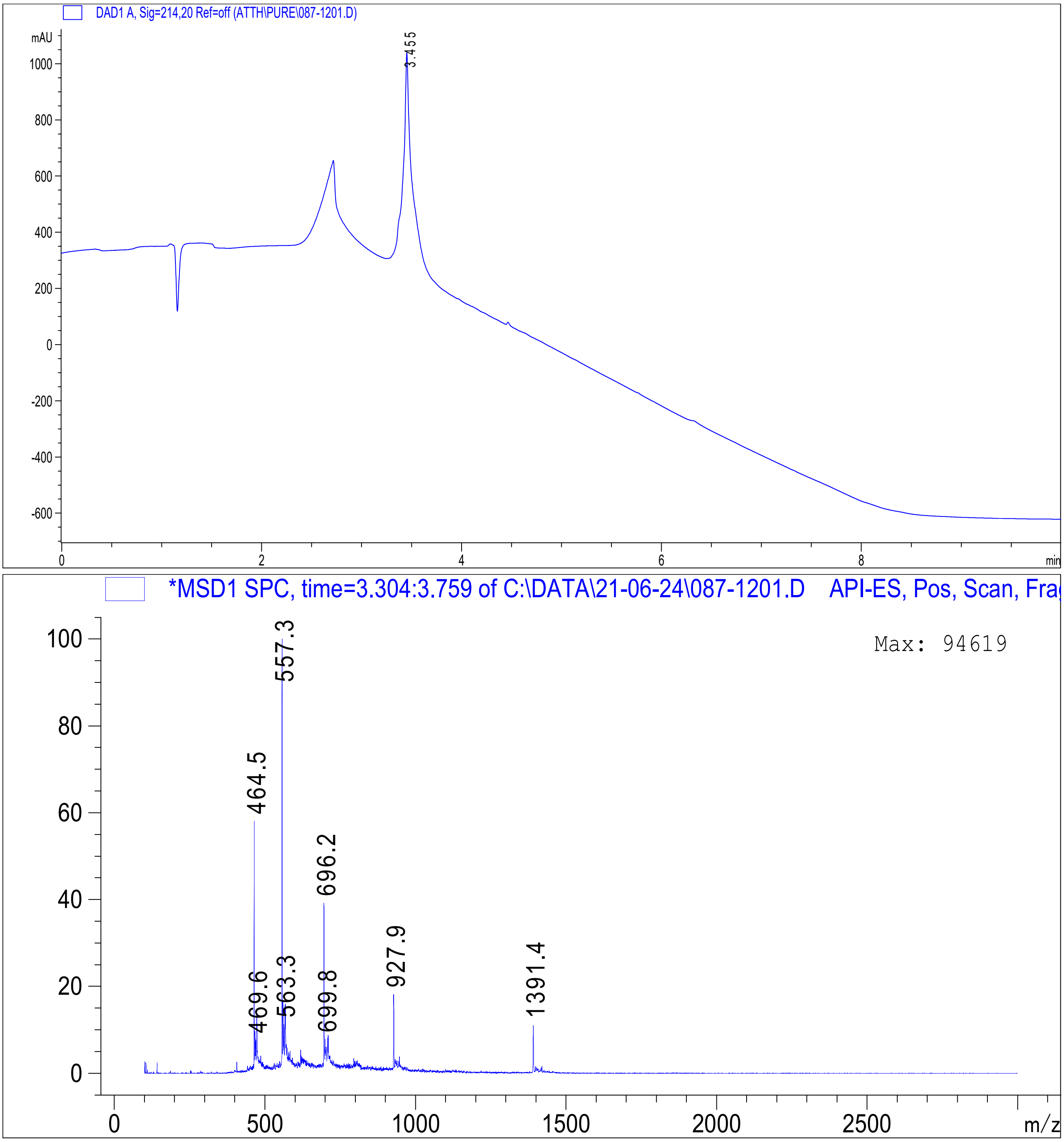

Fig. S52: HPLC-MS chromatogram of purified **bRHAU-2**. HPLC-UV trace at 214 nm (top) and MS spectrum of the corresponding peak (bottom). Calcd MW: 2782.3. For chromatographic conditions, please refer to the general information section.

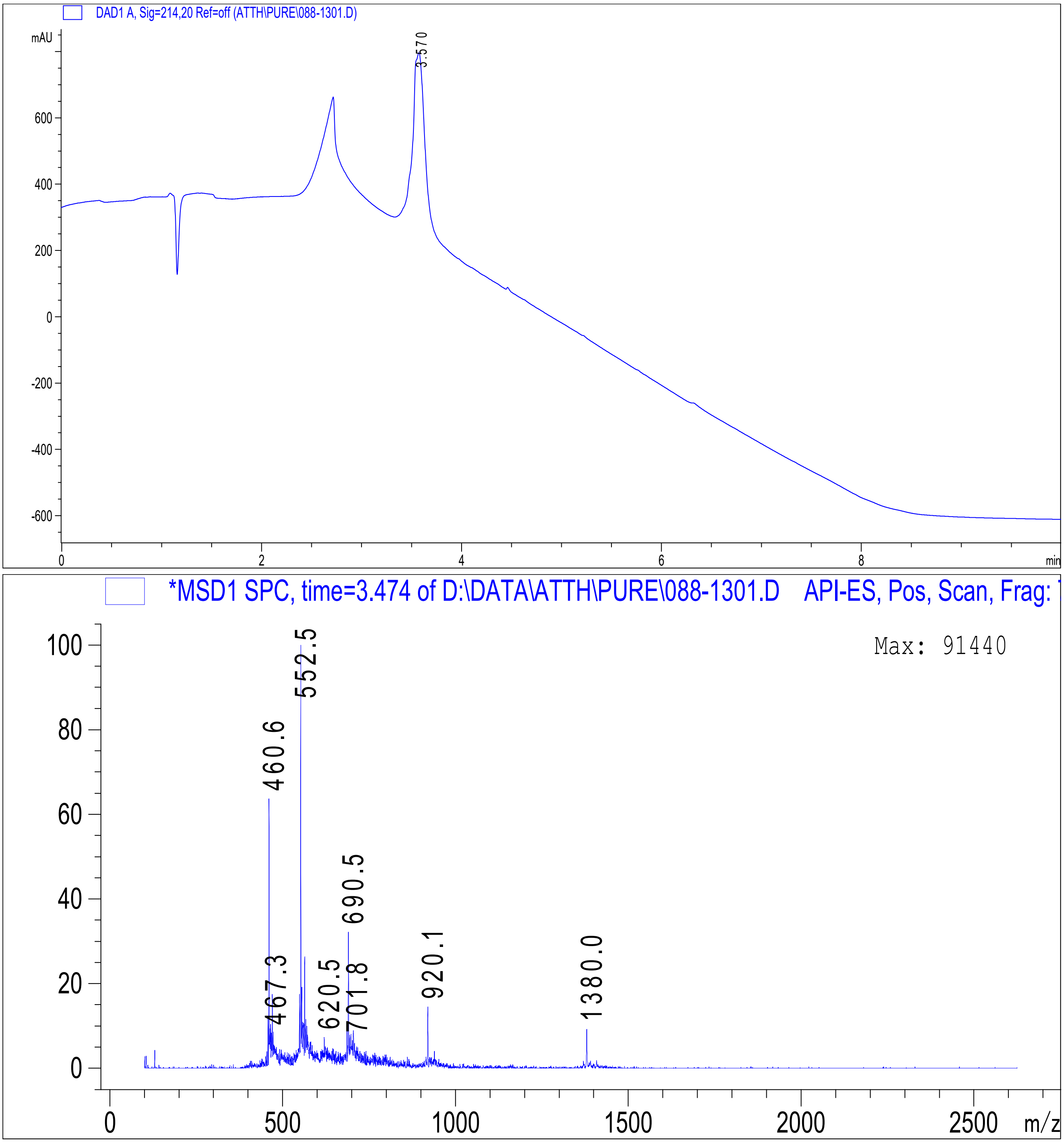

Fig. S53: HPLC-MS chromatogram of purified **bRHAU-3**. HPLC-UV trace at 214 nm (top) and MS spectrum of the corresponding peak (bottom). Calcd MW: 2741.2. Signals relative to TM+18 are also visible in the ESI-MS spectra. For chromatographic conditions, please refer to the general information section.

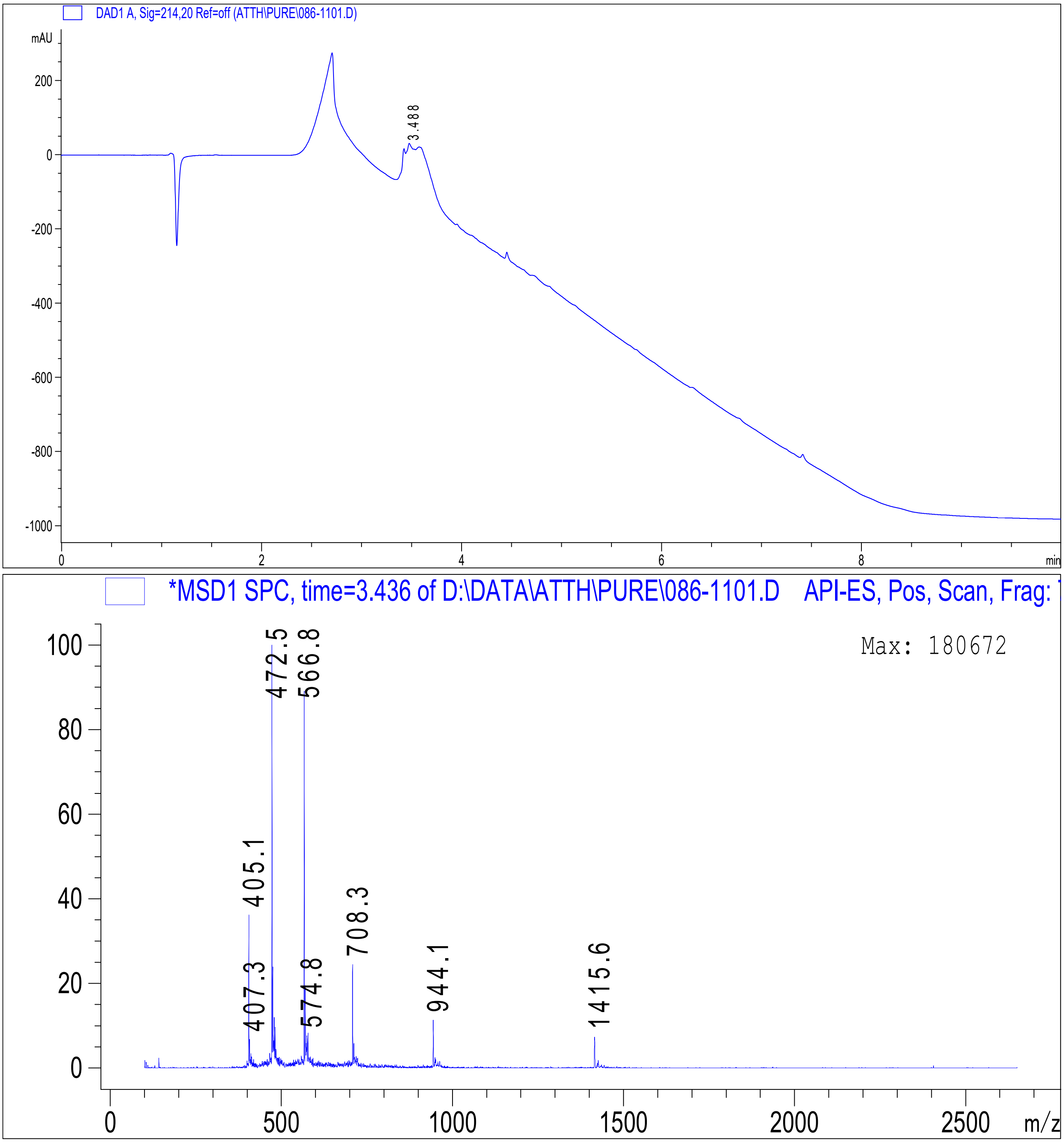

Fig. S54: HPLC-MS chromatogram of purified **bRHAU-4**. HPLC-UV trace at 254 nm (top) and MS spectrum of the corresponding peak (bottom). Calcd MW: 2830.3. For chromatographic conditions, please refer to the general information section.

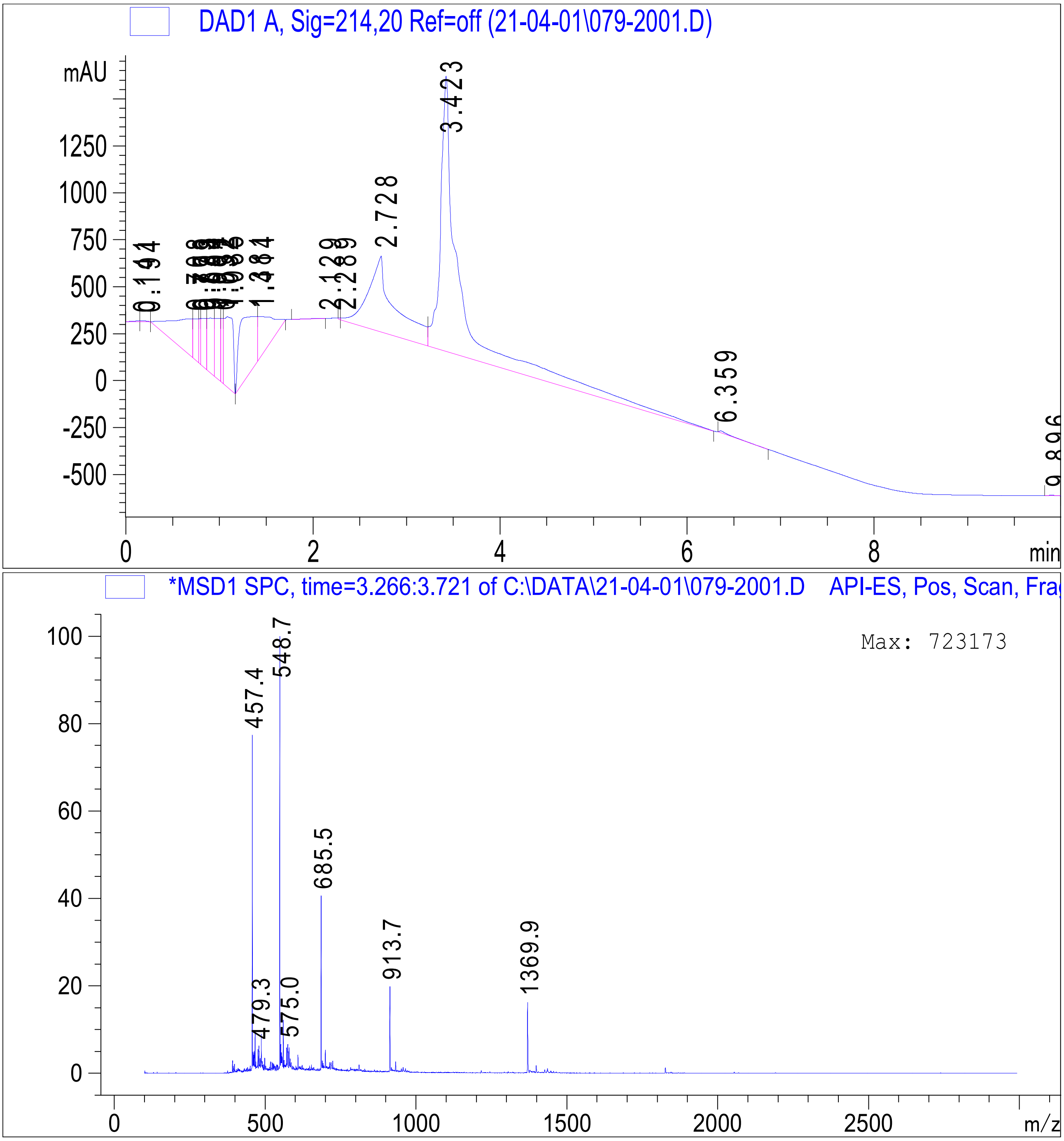

Fig. S55: HPLC-MS chromatogram of purified **bRHAU-5**. HPLC-UV trace at 214 nm (top) and MS spectrum of the corresponding peak (bottom). Calcd MW: 2738.2. For chromatographic conditions, please refer to the general information section.

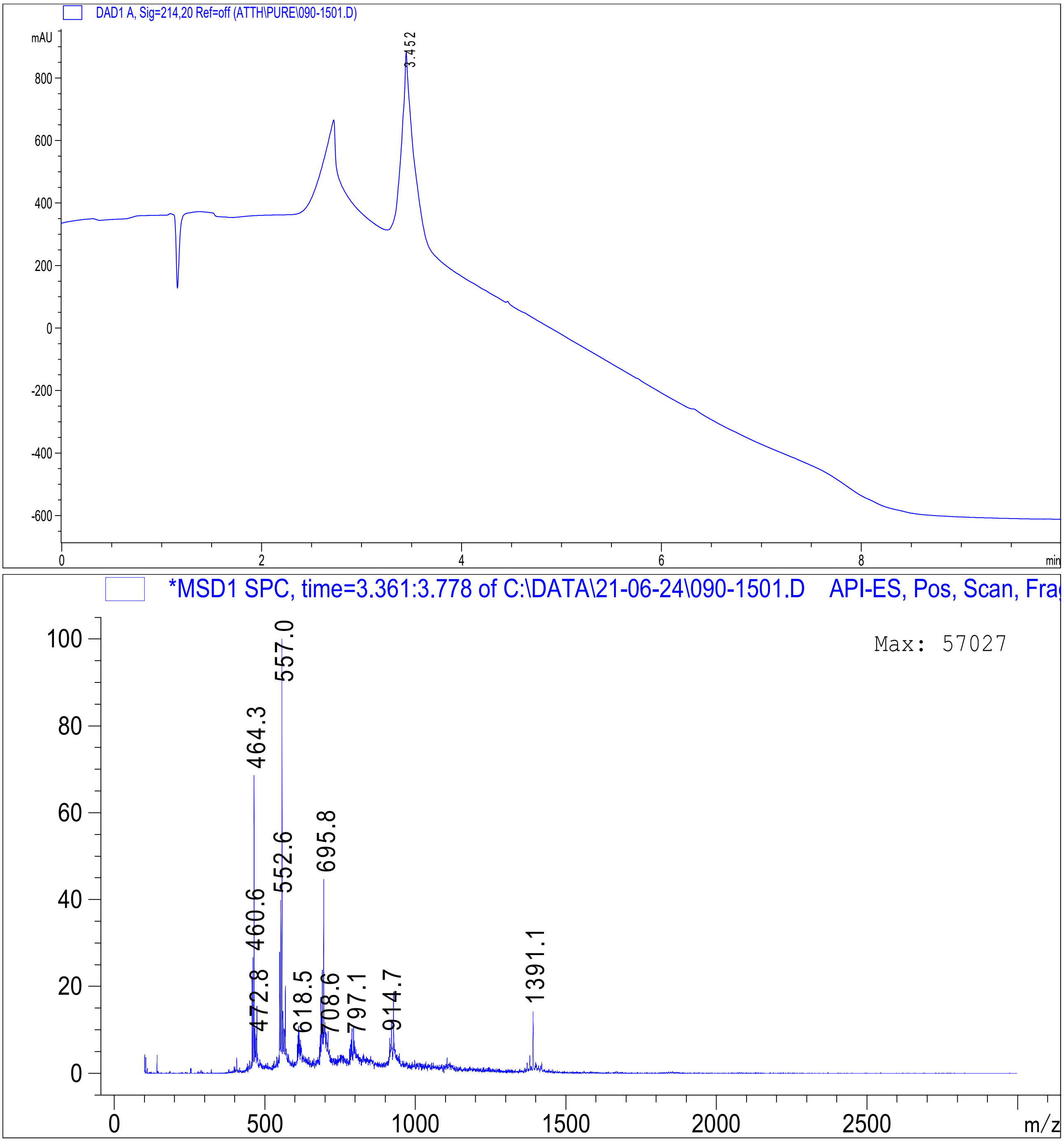

Fig. S56: HPLC-MS chromatogram of purified **bRHAU-6**. HPLC-UV trace at 214 nm (top) and MS spectrum of the corresponding peak (bottom). Calcd MW: 2781.3. For chromatographic conditions, please refer to the general information section.

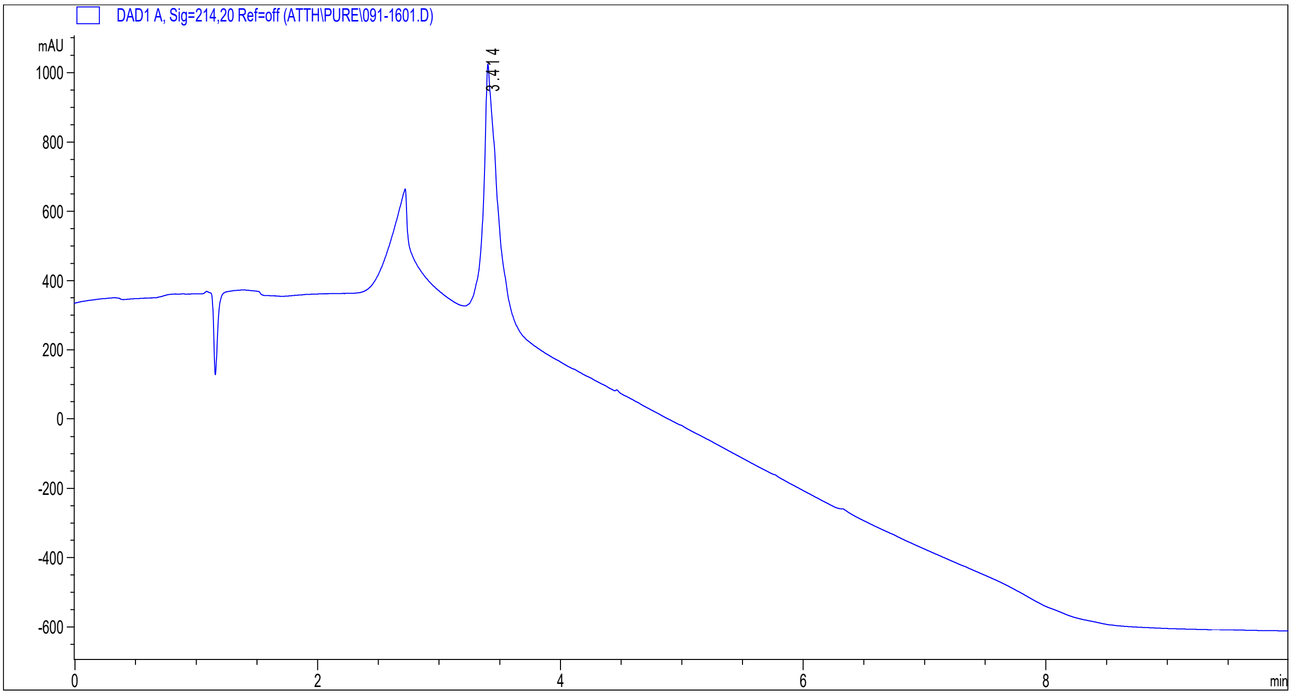

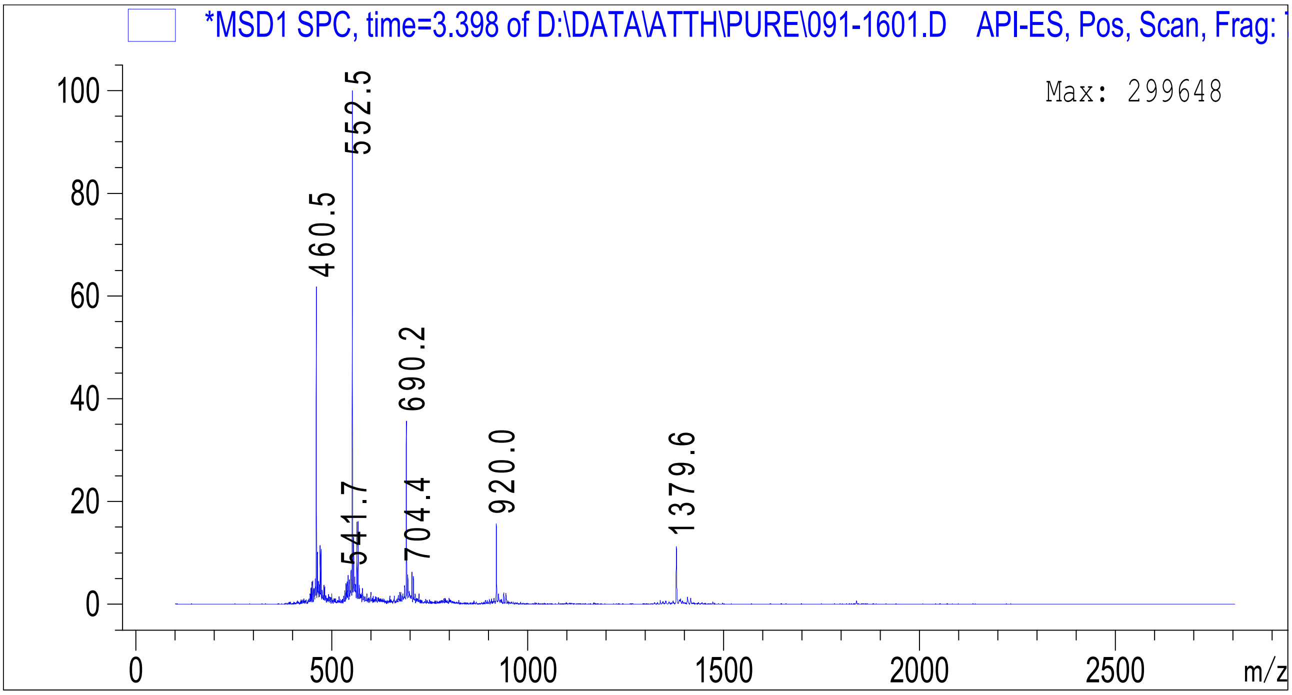

Fig. S57: HPLC-MS chromatogram of purified **bRHAU-7**. HPLC-UV trace at 214 nm (top) and MS spectrum of the corresponding peak (bottom). Calcd MW: 2740.2. Signals relative to TM+18 are also visible in the ESI-MS spectra. For chromatographic conditions, please refer to the general information section.

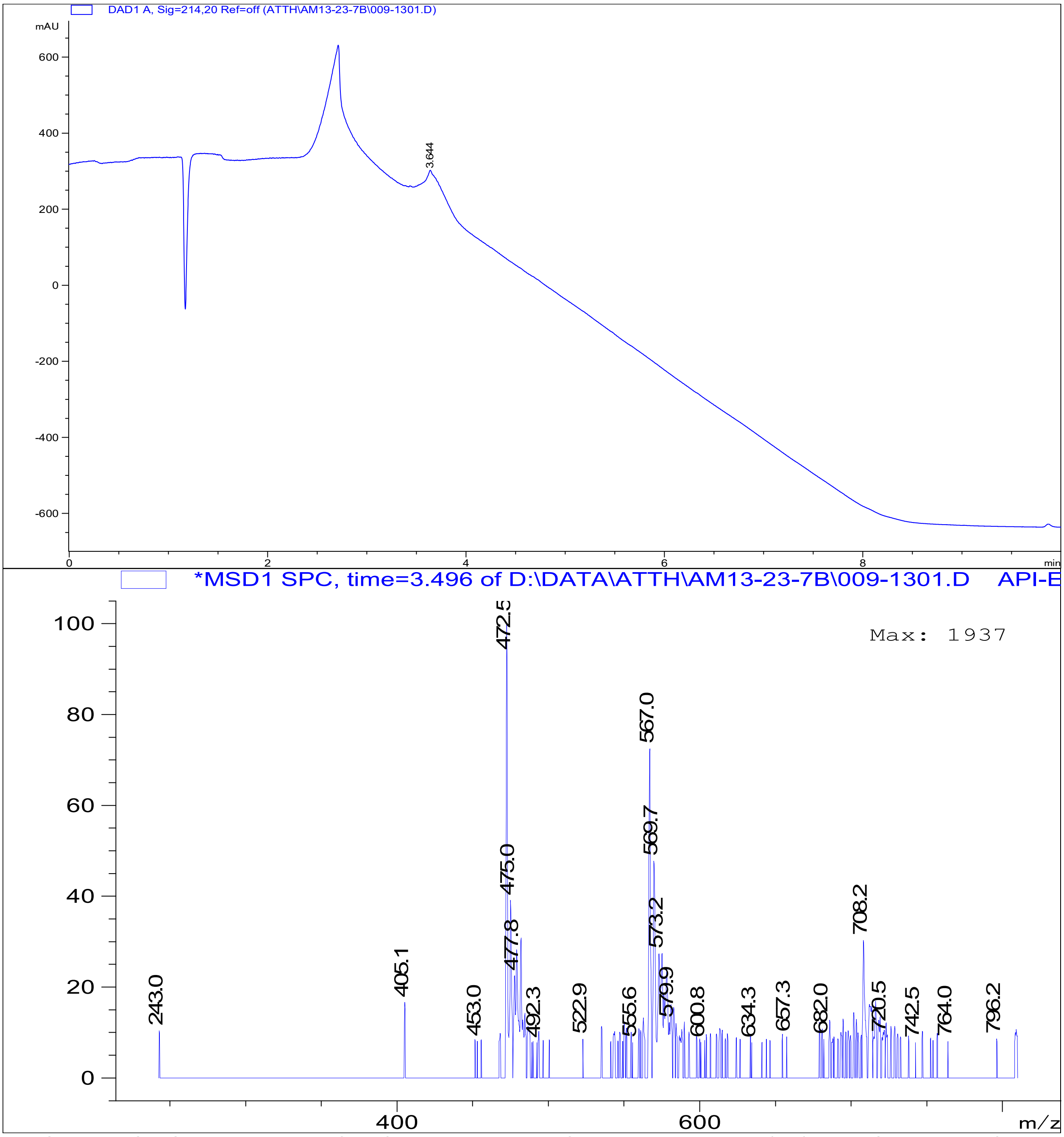

Fig. S58: HPLC-MS chromatogram of purified **bRHAU-8**. HPLC-UV trace at 214 nm (top) and MS spectrum of the corresponding peak (bottom). Calcd MW: 2829.4. For chromatographic conditions, please refer to the general information section.

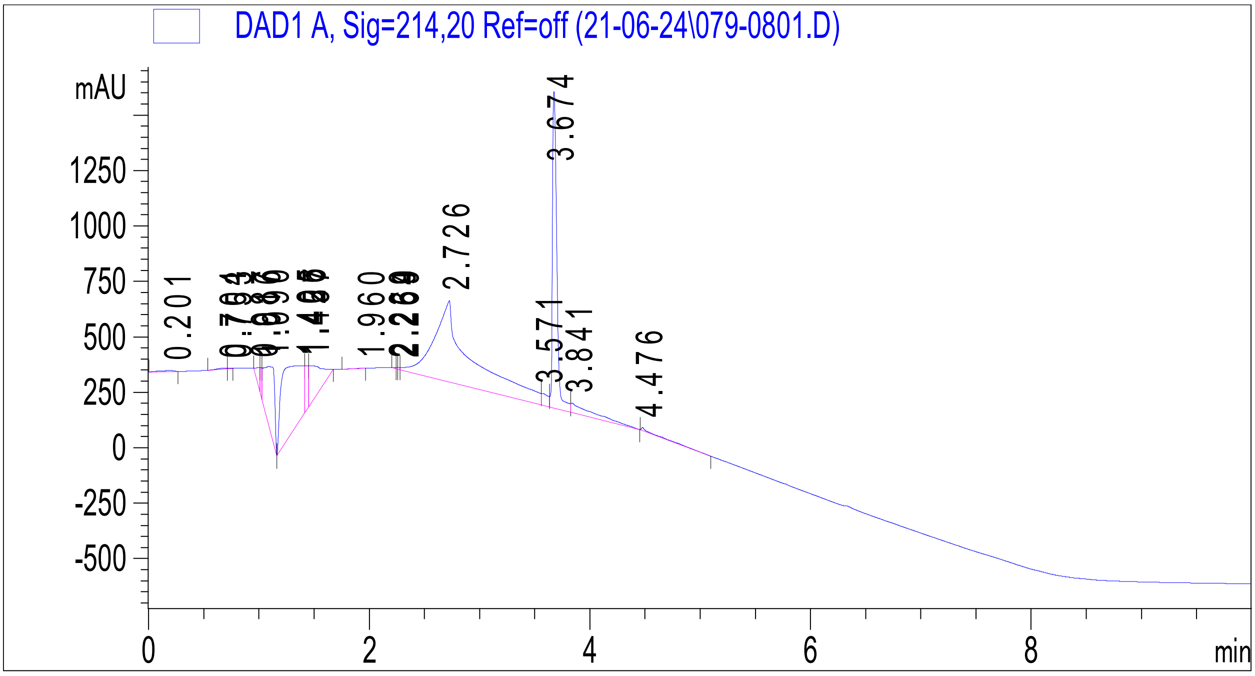

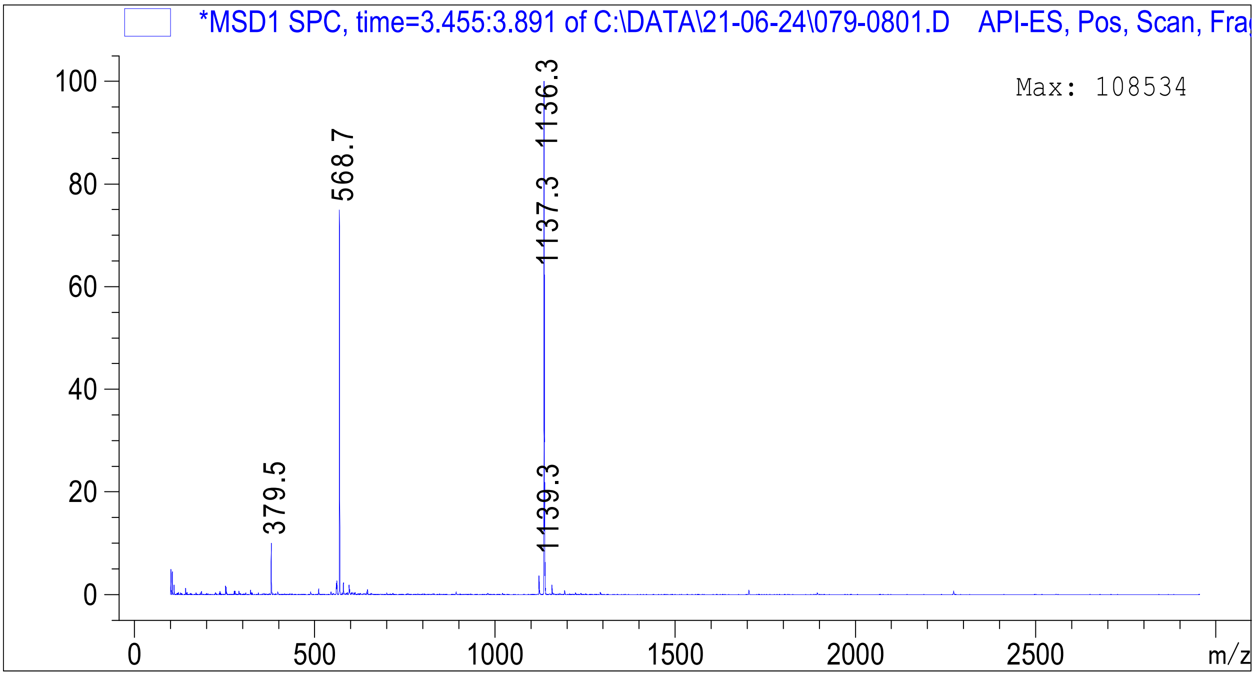

Fig. S59: HPLC-MS chromatogram of purified **RGD-1**. HPLC-mUV trace at 214 nm (top) and MS spectrum of the corresponding peak (bottom). Calcd MW: 1135.5. For chromatographic conditions, please refer to the general information section.

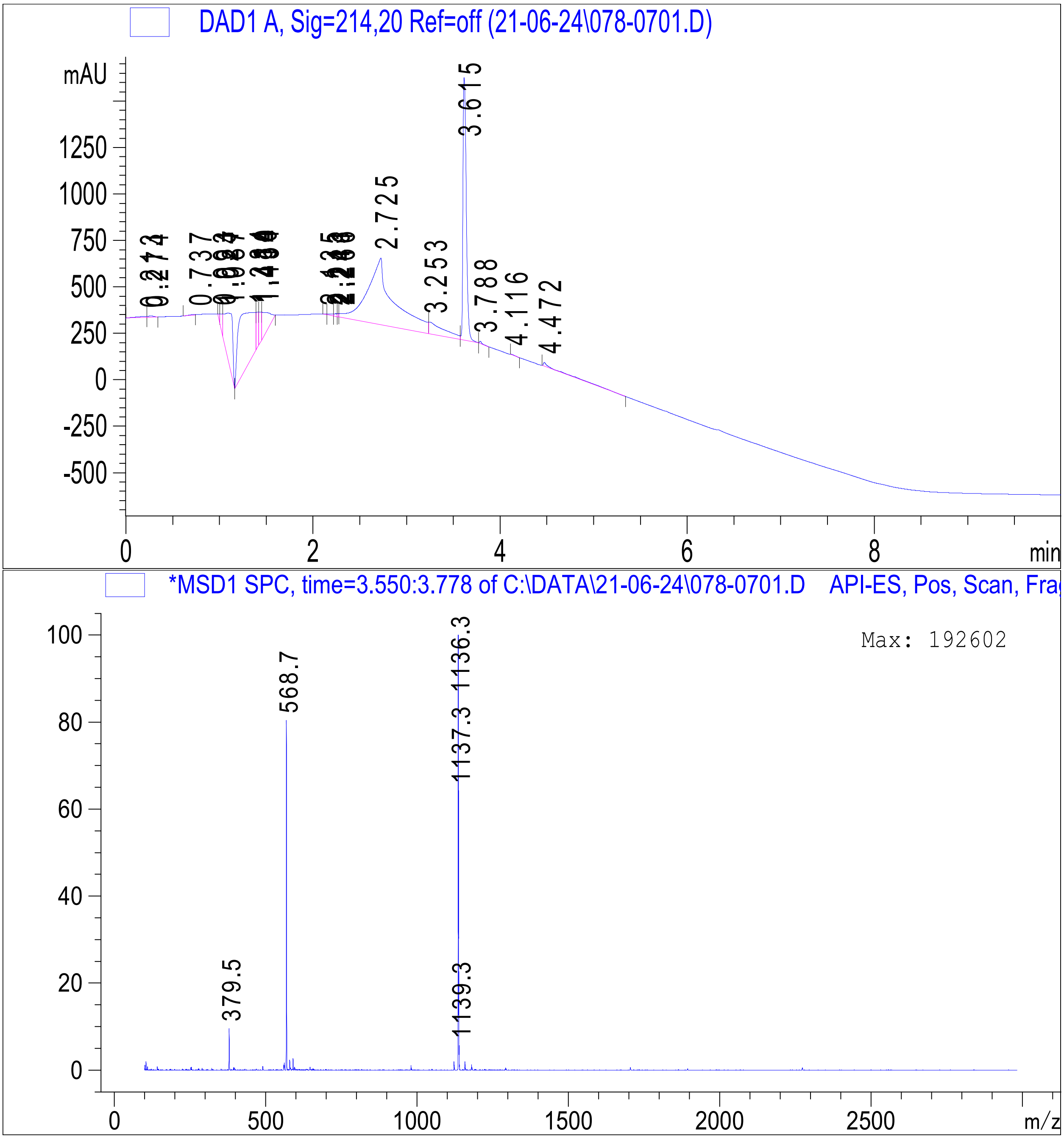

Fig. S60: HPLC-MS chromatogram of purified **RGD-2**. HPLC-UV trace at 214 nm (top) and MS spectrum of the corresponding peak (bottom). Calcd MW: 1135.5. For chromatographic conditions, please refer to the general information section.

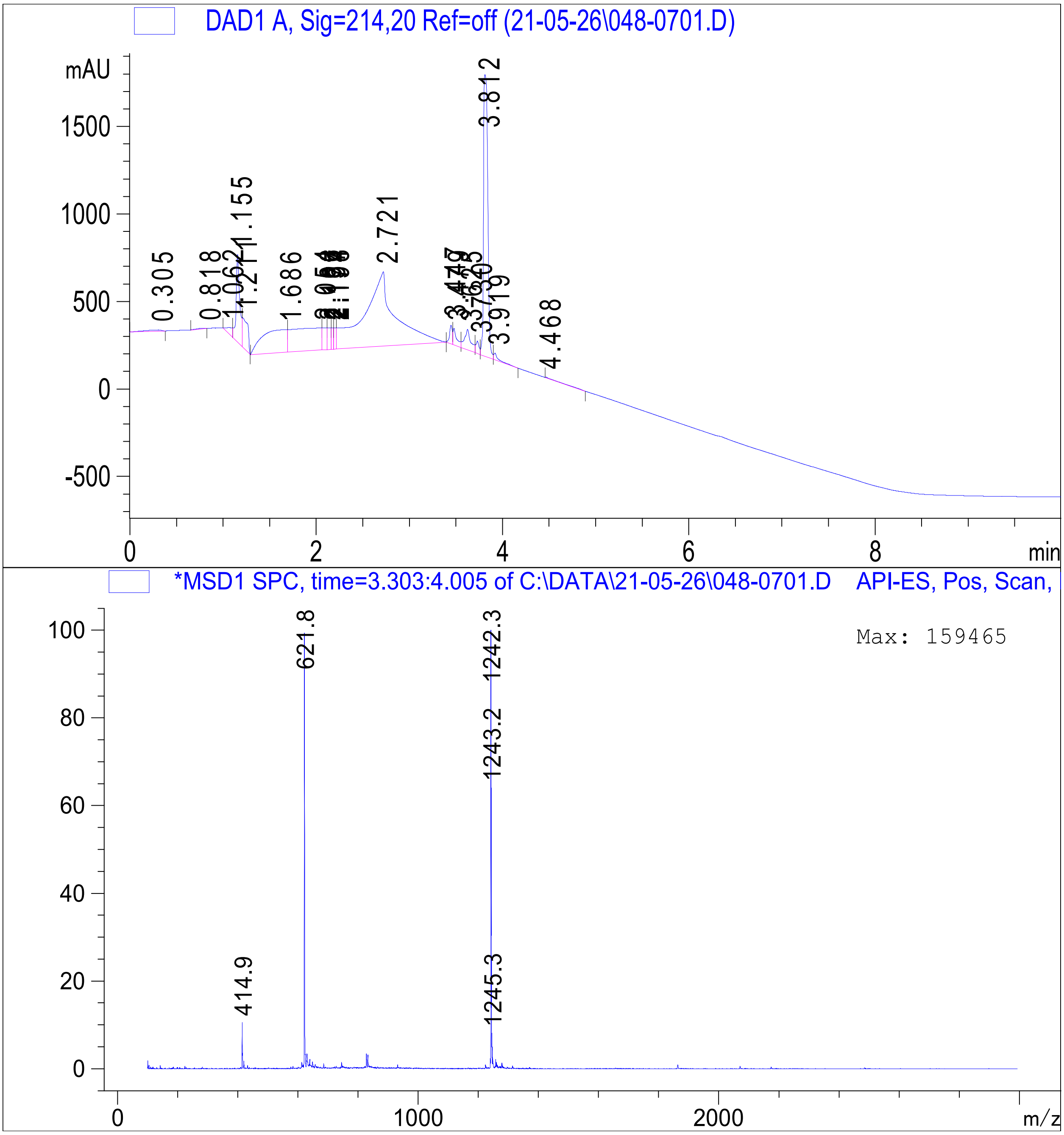

Fig. S61: HPLC-MS chromatogram of purified **cRGD-1**. HPLC-UV trace at 214 nm (top) and MS spectrum of the corresponding peak (bottom). Calcd MW: 1241.3. For chromatographic conditions, please refer to the general information section.

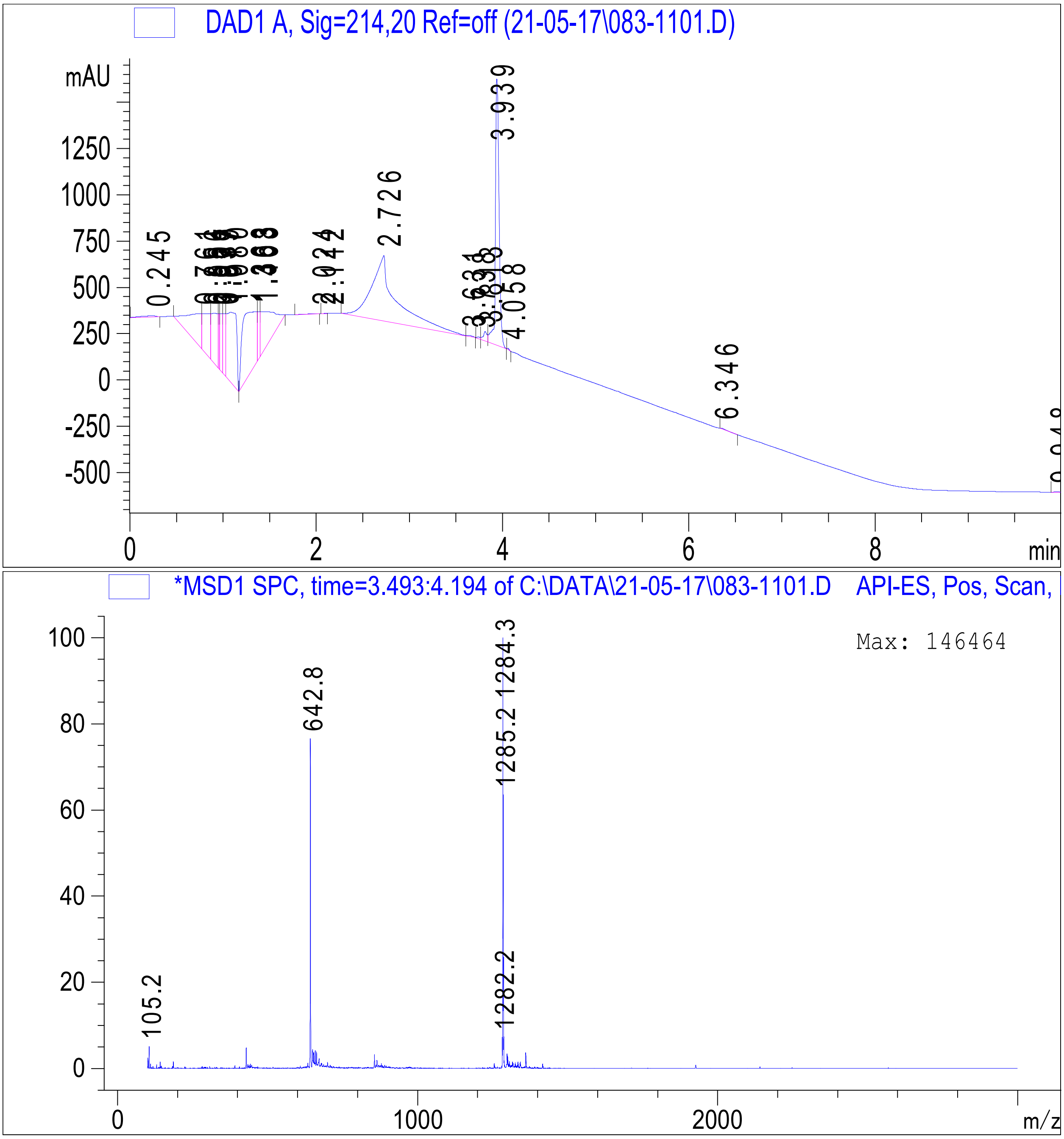

Fig. S62: HPLC-MS chromatogram of purified **cRGD-2**. HPLC-UV trace at 214 nm (top) and MS spectrum of the corresponding peak (bottom). Calcd MW: 1284.4. For chromatographic conditions, please refer to the general information section.

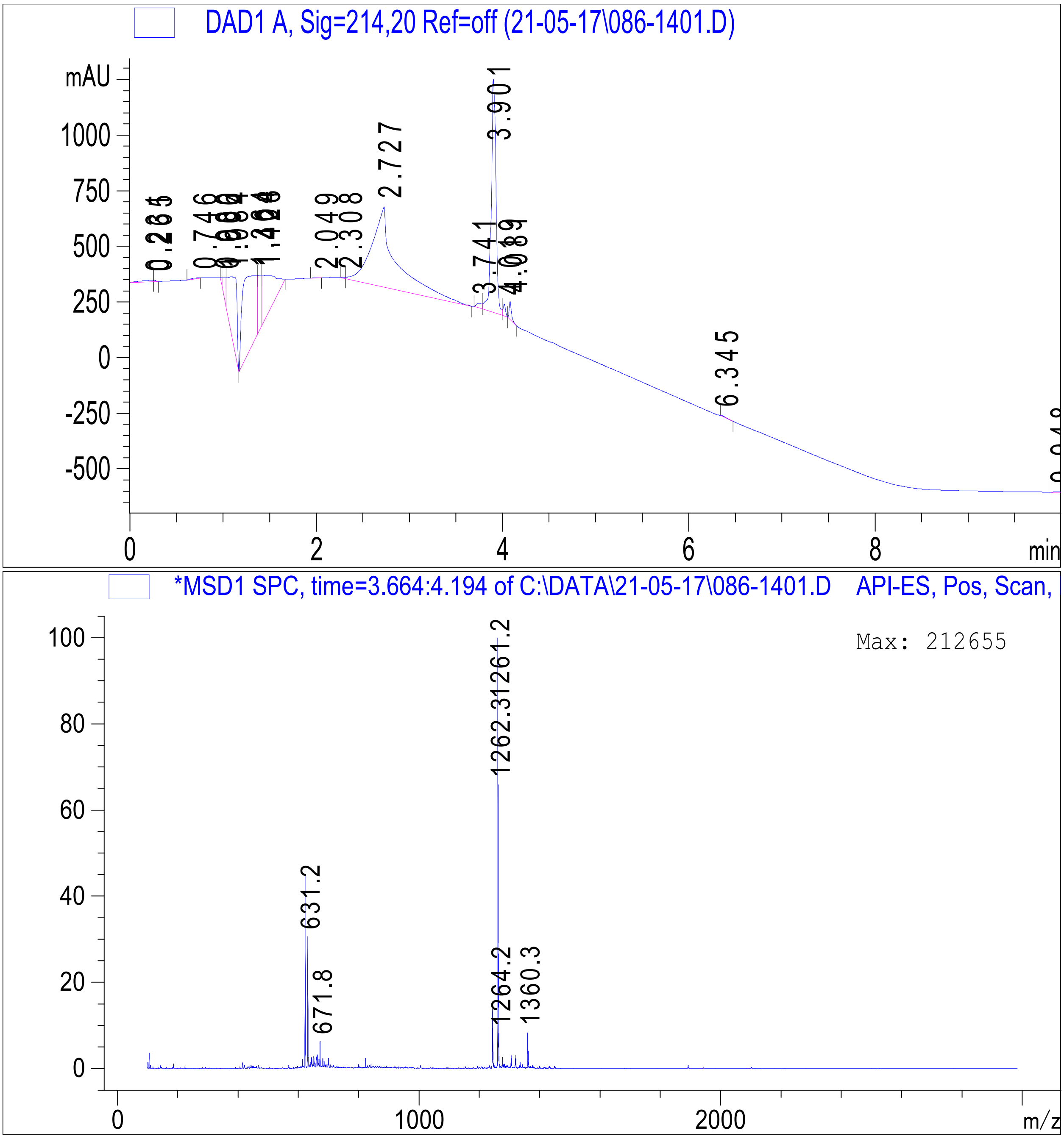

Fig. S63: HPLC-MS chromatogram of purified **cRGD-3**. HPLC-UV trace at 214 nm (top) and MS spectrum of the corresponding peak (bottom). Calcd MW: 1243.3. TM+18 signals are also visible in the ESI-MS spectra. For chromatographic conditions, please refer to the general information section.

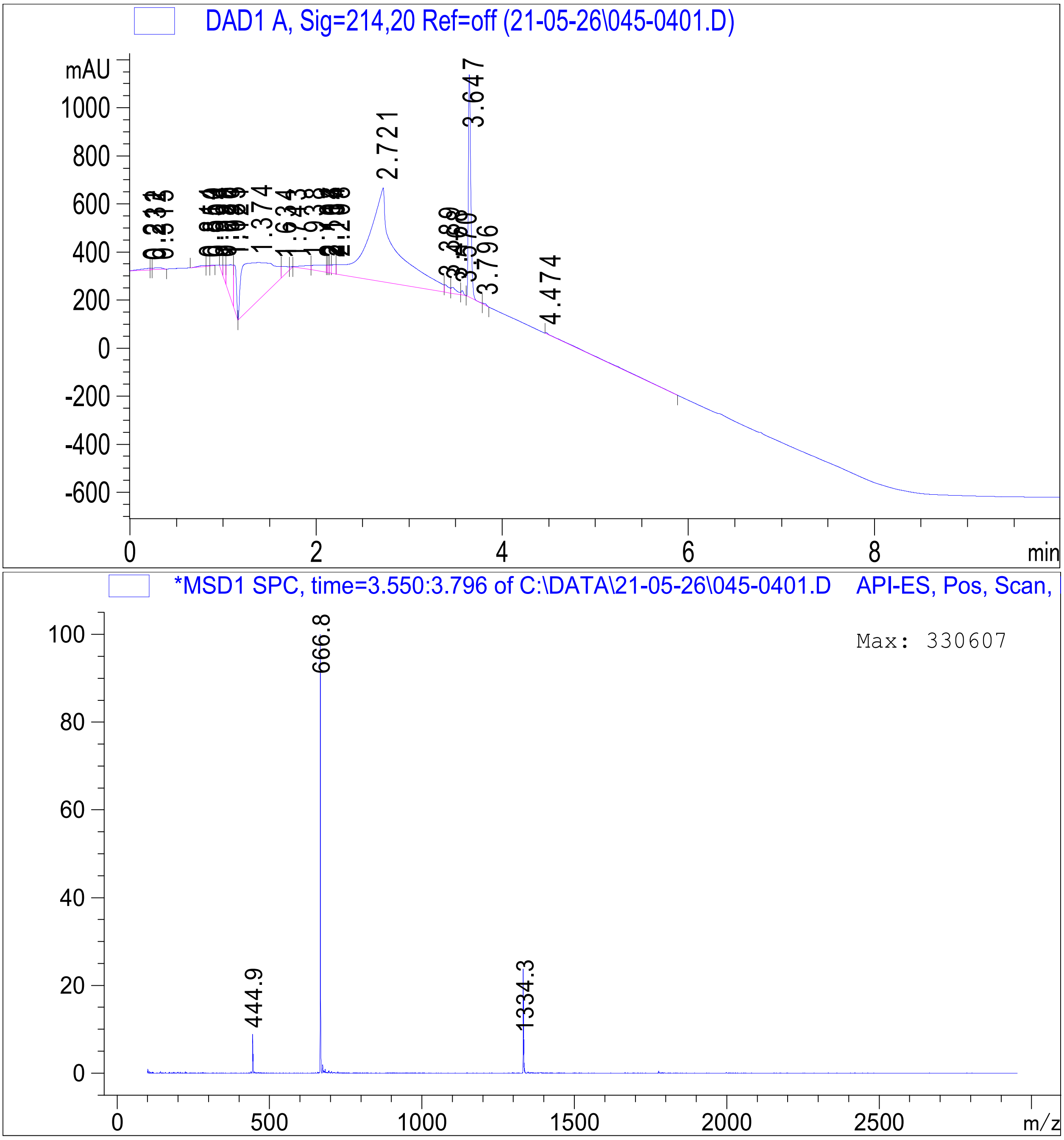

Fig. S64: HPLC-MS chromatogram of purified **cRGD-4**. HPLC-UV trace at 214 nm (top) and MS spectrum of the corresponding peak (bottom). Calcd MW: 1333.4. For chromatographic conditions, please refer to the general information section.

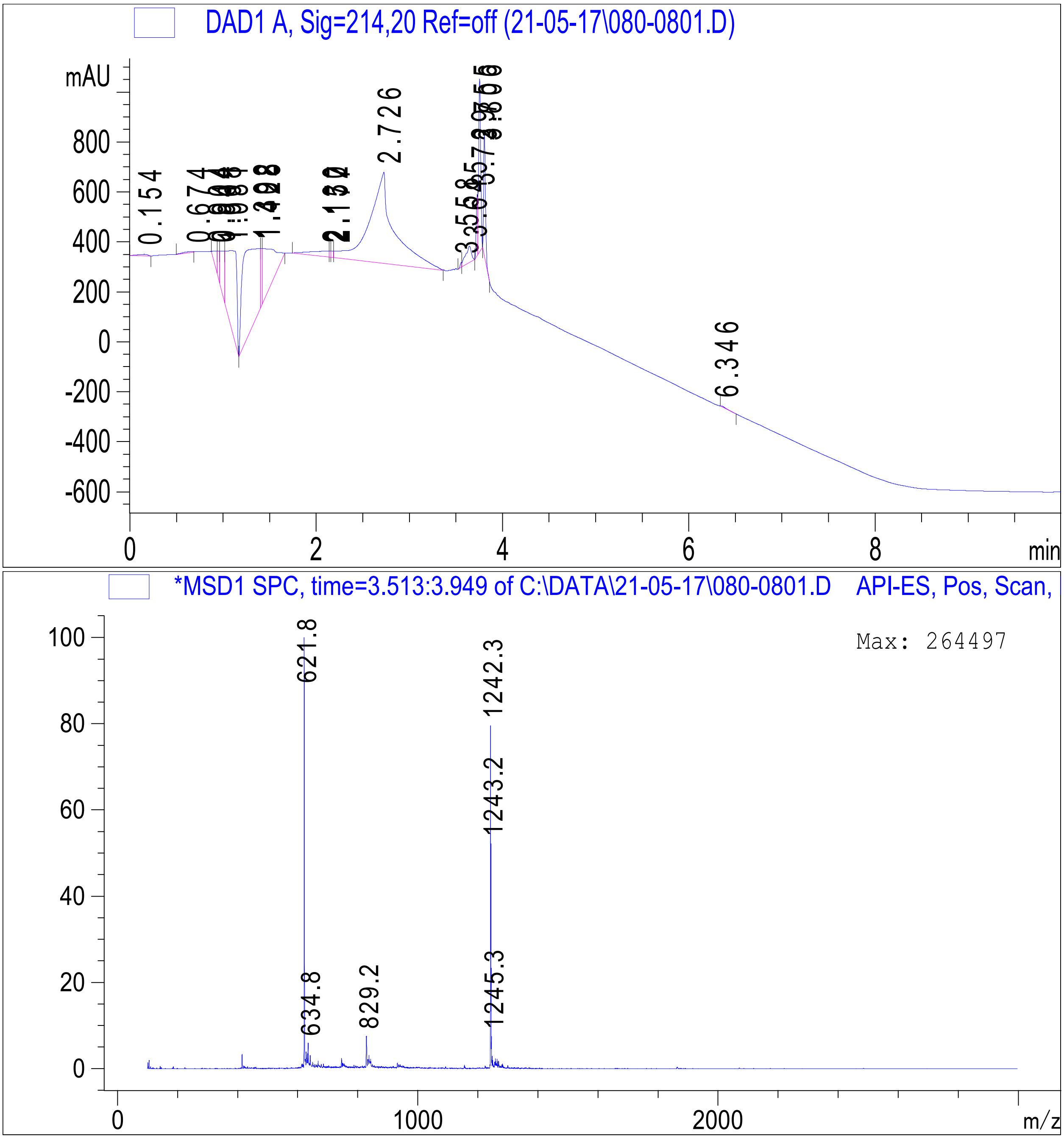

Fig. S65: HPLC-MS chromatogram of purified **cRGD-5**. HPLC-UV trace at 214 nm (top) and MS spectrum of the corresponding peak (bottom). Calcd MW: 1241.3. For chromatographic conditions, please refer to the general information section.

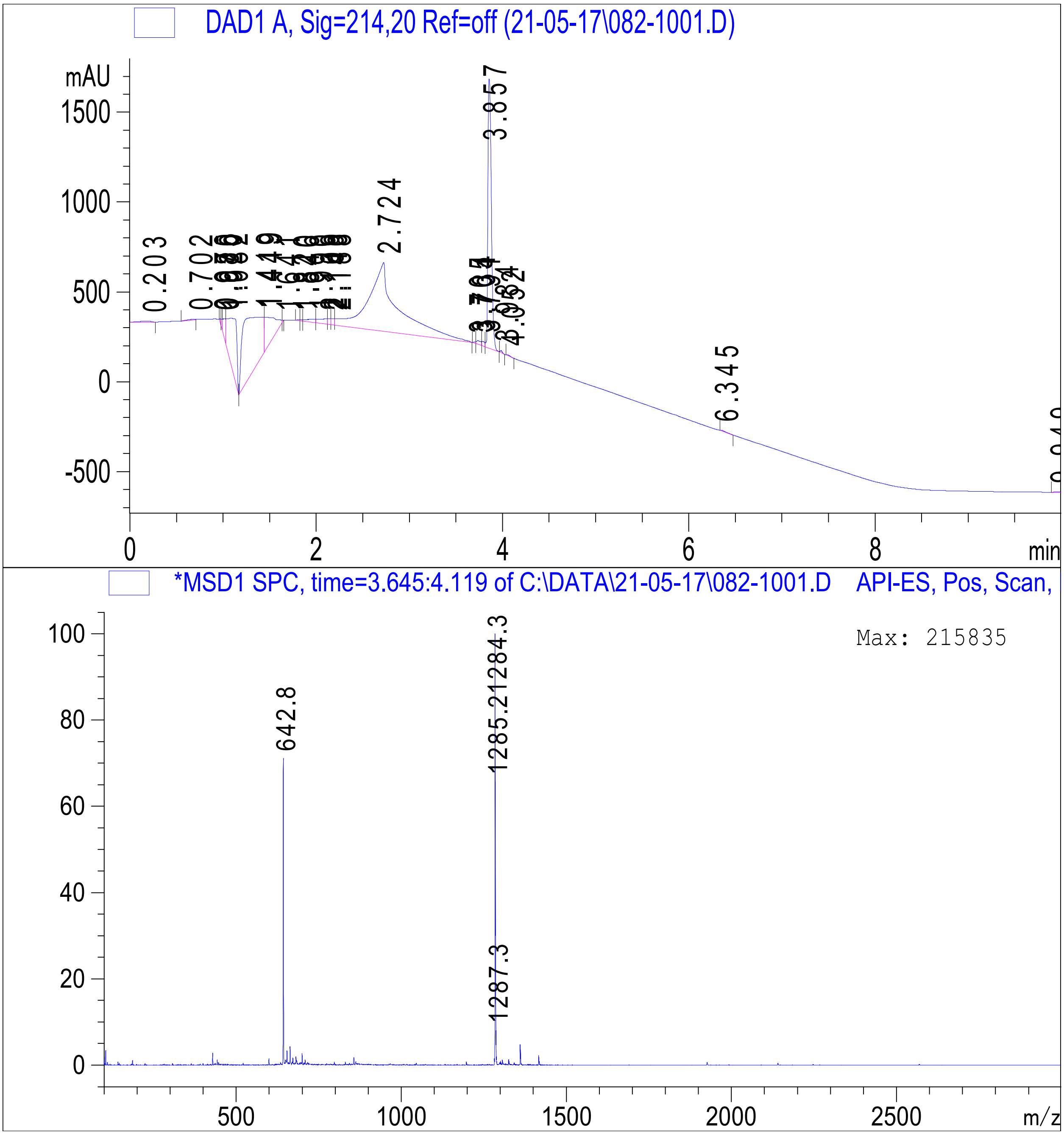

Fig. S66: HPLC-MS chromatogram of purified **cRGD-6.** HPLC-UV trace at 214 nm (top) and MS spectrum of the corresponding peak (bottom). Calcd MW: 1284.4. For chromatographic conditions, please refer to the general information section.

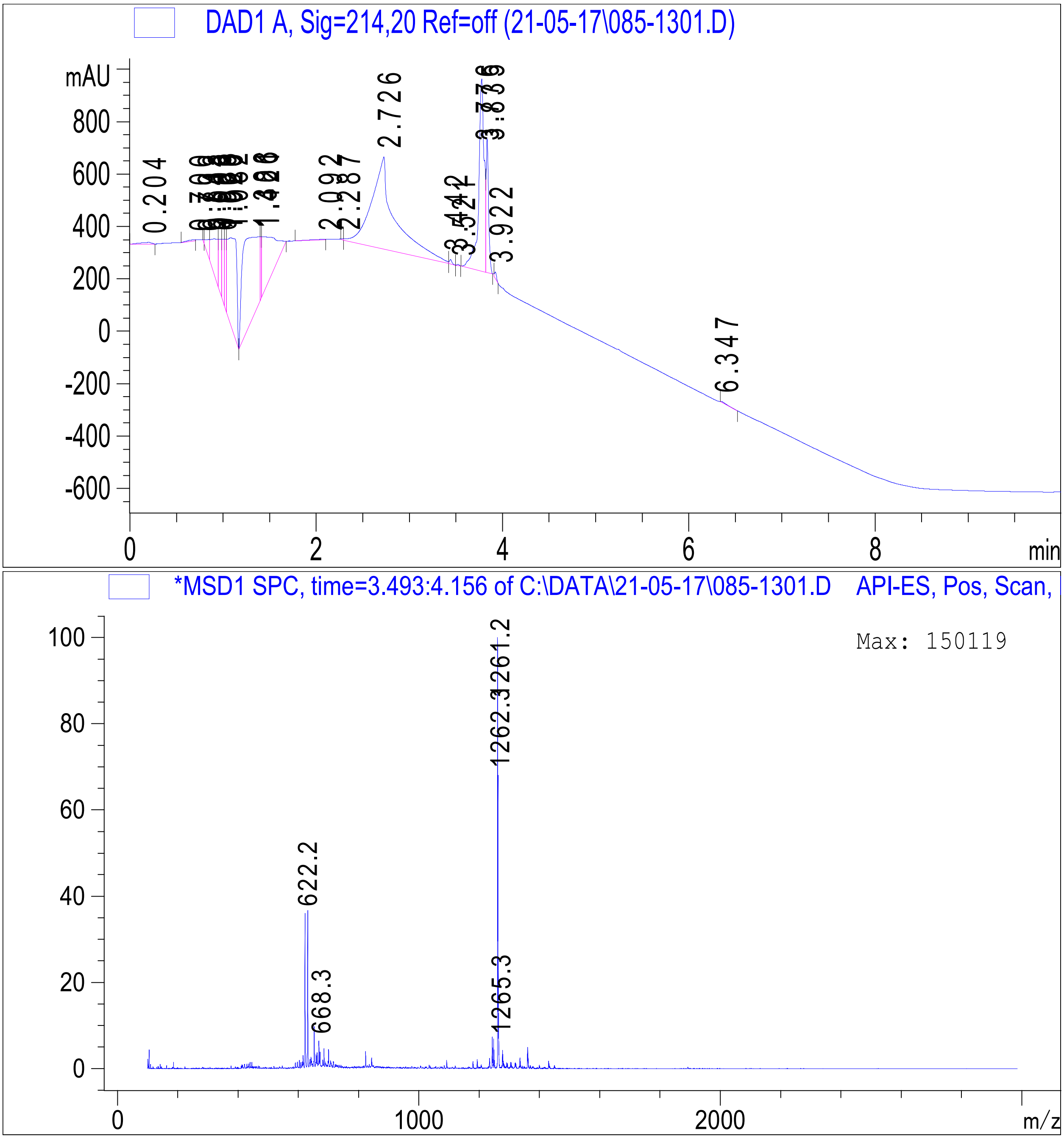

Fig. S67: HPLC-MS chromatogram of purified **cRGD-7**. HPLC-UV trace at 214 nm (top) and MS spectrum of the corresponding peak (bottom). Calcd MW: 1243.3. For chromatographic conditions, please refer to the general information section.

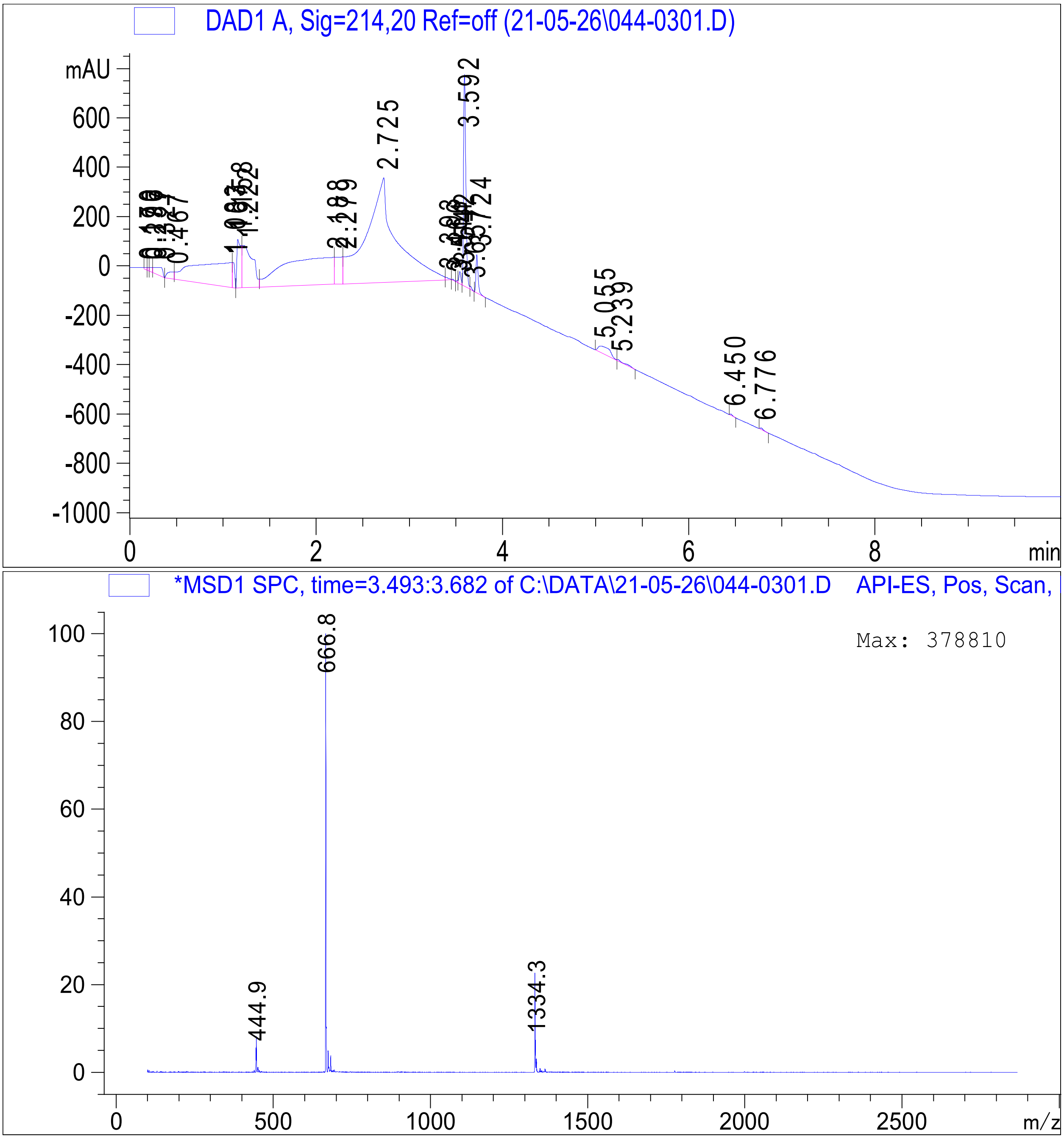

Fig. S68: HPLC-MS chromatogram of purified **cRGD-8**. HPLC-UV trace at 214 nm (top) and MS spectrum of the corresponding peak (bottom). Calcd MW: 1333.4. For chromatographic conditions, please refer to the general information section.
